# Supplementary material for: Examining non-linearity in the association between age and reported opioid use in different socioeconomic strata: cohort study using Health Survey for England waves from 1997 to 2014
Source: BMJ Open. 2023 Mar 1;13(3):e057428. doi: 10.1136/bmjopen-2021-057428 (PMC9980331; doi:10.1136/bmjopen-2021-057428)

The nonlinear association between age and socioeconomic status on reported opioid use: cohort study using health survey for England waves from 1997 to 2014

Supplementary Materials: Sensitivity Analysis

Table SM1: Semi-parametric generalised additive models for the odds of reported prescription opioid use with smooth function for the effect of age, varying by SES measure in Females only

	Opioid use (Model including income	Opioid use (Model including employment status)	Opioid use (Model including education)
**Parametric coefficients**	**OR (95% CI)**	**OR (95% CI)**	**OR (95% CI)**
Intercept	0.01 (0.01:0.01) ***	0.004 (0.003:0.006) ***	0.012 (0.009:0.159) ***
White (ref: Not white)	2.11 (1.66:2.67) ***	1.91 (1.51:2.43)***	1.90 (1.50:2.41)***
Urban (ref: Rural)	1.30 (1.14:1.48) ***	1.23 (1.08:1.40)**	1.31 (1.14:1.49)***
Income Quantile (ref: 1-Lowest)			
Income 2	0.98 (0.81:1.19)		
Income 3	0.71 (0.57:0.87) ***		
Income 4	0.47 (0.38:0.59) ***		
Income 5 - Highest	0.41 (0.32:0.52) ***		
Income Missing	0.75 (0.60:0.93) **		
Economic Status (ref: Employed)			
Domestic		1.59 (1.29:1.97) ***	
Ill or disabled		17.52 ***(12.97:23.66)	
Other		3.04 (1.70:5.43)***	
Retired		1.75 (1.70:5.43)*	
Student		0.75 (0.19:2.80)	
Unemployed		1.78 (0.83:3.81)	
Education (ref: No qualification)			
Higher education			0.51 (0.41:0.62)***
A-levels			0.53 (0.41:0.69) ***
O-levels/GSE			0.71 (0.58:0.85)***
Foreign/Other			0.61 (0.39:0.94) *
Student			0.48 (0.31:0.75)**
**Non Parametric**	**EDF**	**EDF**	**EDF**
**Income Quantile**			
Income 1 - lowest	4.91 ***		
Income 2	3.52 ***		
Income 3	2.56 ***		
Income 4	1.12 ***		
Income 5 - highest	1.91 ***		
Income Missing	3.01 ***		

Employment status			
Domestic		2.669***	
Ill or disabled		2.626 *	
Other		1.428	
Retired		4.013 ***	
Student		1.000	
Unemployed		1.002	
Employed		1.003 ***	
Education			
Higher education			2.108***
A-levels			2.646***
O-levels/GSE			2.302***
No qualifications			3.631***
Foreign/Other			1.049*
FT Student			2.062*

Table SM2: Semi-parametric generalised additive models for the odds of reported prescription opioid use with smooth function for the effect of age, varying by SES measure in Males only

	**Opioid use (Model including income**	**Opioid use (Model including employment status)**	**Opioid use (Model including education)**
**Parametric coefficients**	**OR (95% CI)**	**OR (95% CI)**	**OR (95% CI)**
Intercept	0.01 (0.01:0.01) ***	0.003 (0.002:0.004)***	0.009 (0.006:0.13)***
White (ref: Not white)	2.48 (1.84:3.35) ***	2.17 (1.60:2.93)***	2.02***
Urban (ref: Rural)	1.24 (1.05:1.46) **	1.13 (0.96:1.34)	1.26**
Income Quintile (ref: 1-Lowest)	ref		
Income 2	0.85 (0.66:1.09)		
Income 3	0.34 (0.25:0.47) ***		
Income 4	0.24 (0.17:0.34) ***		
Income 5 - highest	0.30 (0.22:0.40) ***		
Income Missing	0.54 (0.41:0.71) ***		
Economic Status (ref: Employed))			
Domestic		2.82 (1.40:5.66)**	
Ill or disabled		15.51 (11.40:21.10)***	
Other		3.57 (1.89:6.74)***	
Retired		3.80 (2.66:5.43)****	
Student		0.12 (0.0002:89.32)	
Unemployed		0.33 (0.02:4.08)	
Education (ref: No qualification)			
Higher education			0.44 (0.34:0.57)***
A-levels			0.56 (0.40:0.79)***
O-levels/GSE			0.70 (0.54:0.91)**
Foreign/Other			0.65 (0.31:1.39)
Student			0.63 (0.39:1.06)
**Non Parametric**	**EDF**	**EDF**	**EDF**
**Income Quantile**			
Income 1 - lowest	4.73 ***		
Income 2	3.89 ***		
Income 3	2.69 ***		
Income 4	2.79 ***		
Income 5 - highest	1.13 ***		
Income Missing	2.89 ***		

Employment status			
Employed		1.300***	
Domestic		1.001*	
Ill or disabled		2.900**	
Other		1.002*	
Retired		1.002	
Student		2.742	
Unemployed		3.232	
Education			
Higher education			1.002***
A-levels			2.878***
O-levels/GSE			3.929***
No qualifications			3.695***
Foreign/Other			1.002
Student			2.741***

Figure SM1: Non-parametric estimation of the effect of age on likelihood of reported opioid use by income quantile in females

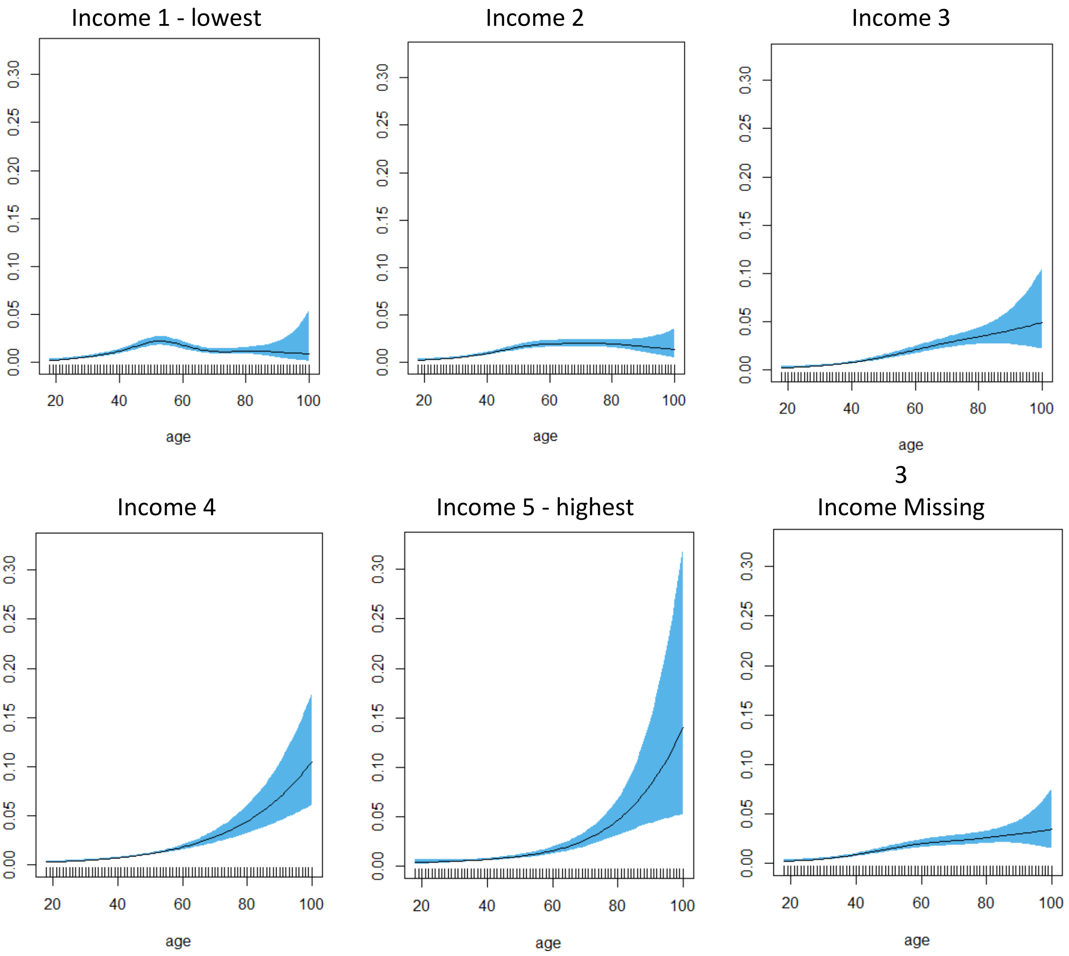

Figure SM2: Non-parametric estimation of the effect of age on likelihood of reported opioid use by employment status in females

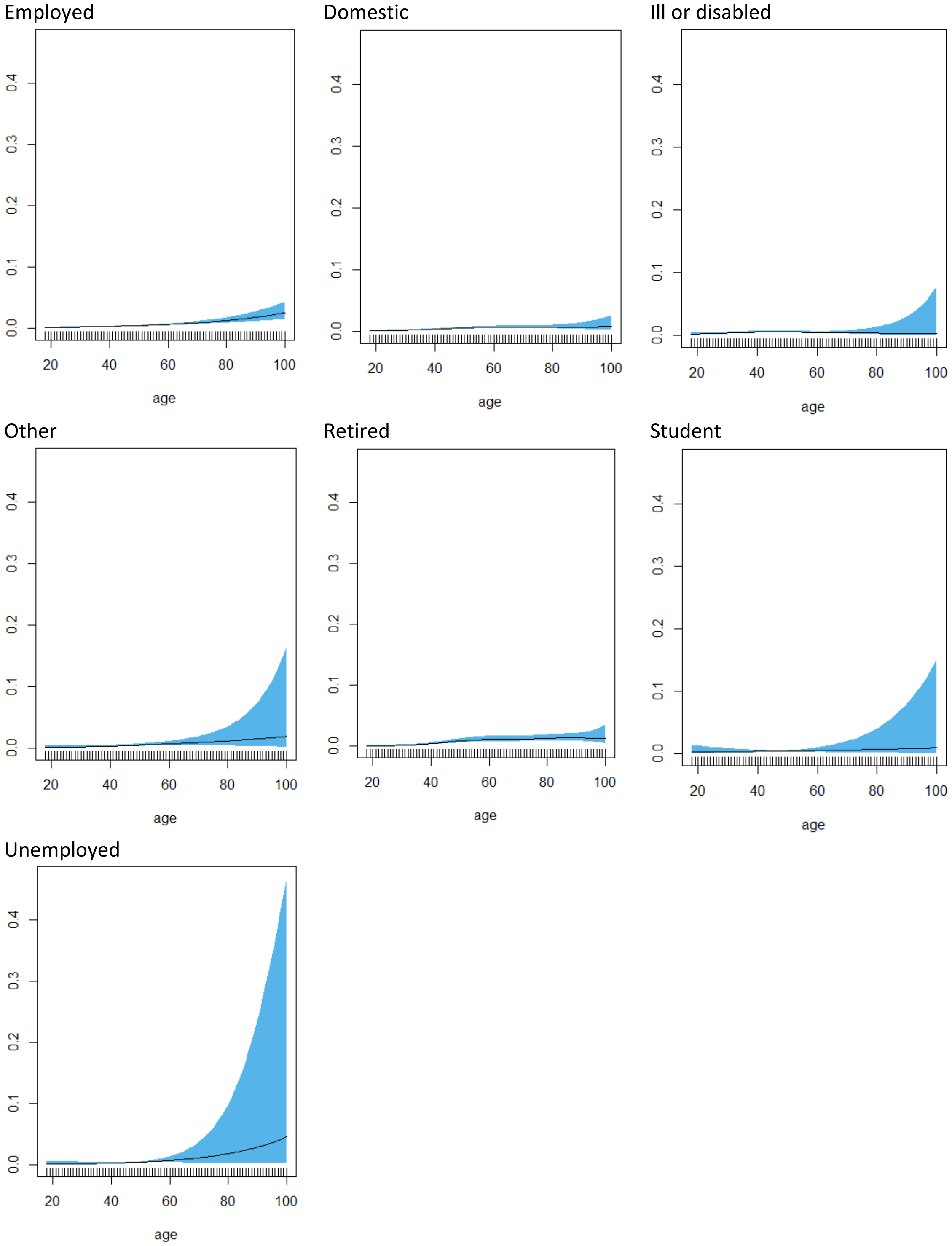

Figure SM3: Non-parametric estimation of the effect of age on likelihood of reported opioid use by income highest qualification in females

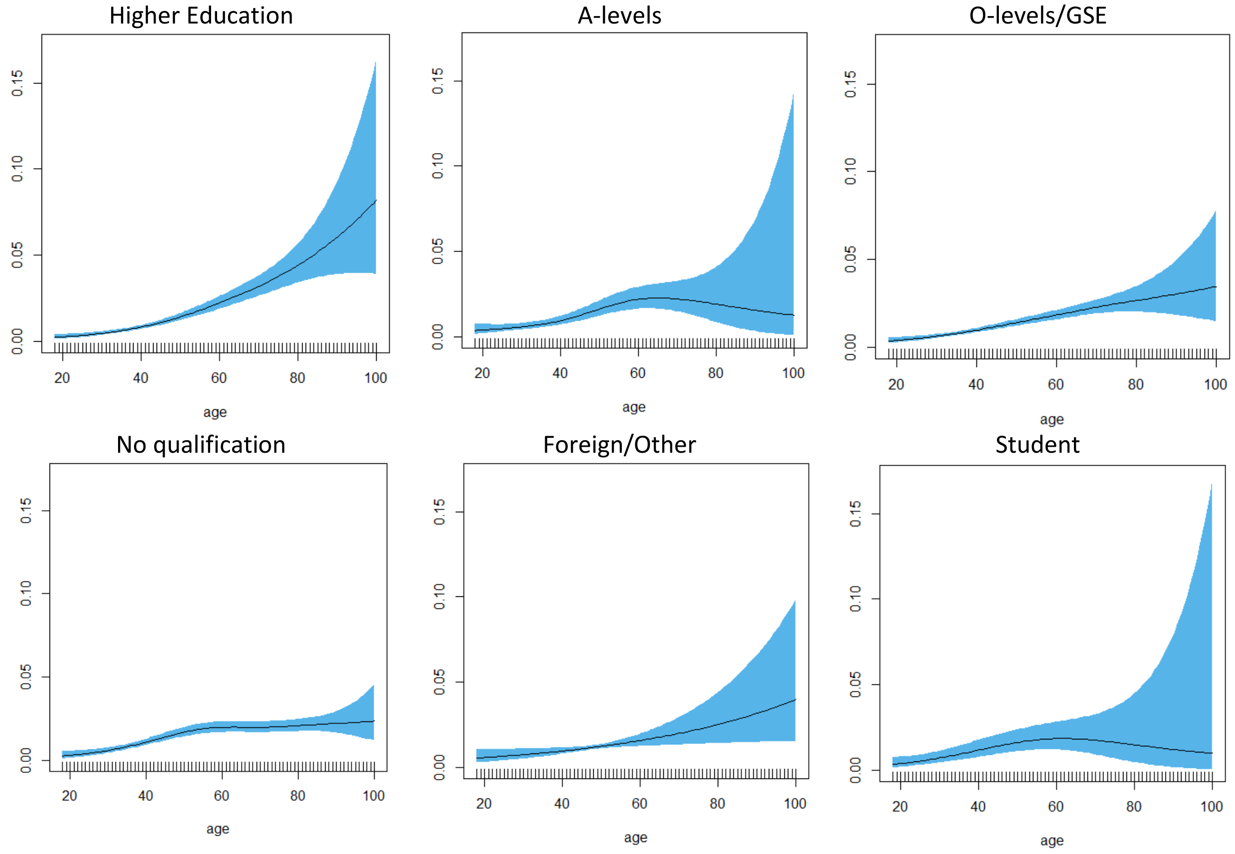

Figure SM4: Non-parametric estimation of the effect of age on likelihood of reported opioid use by income quantile in males

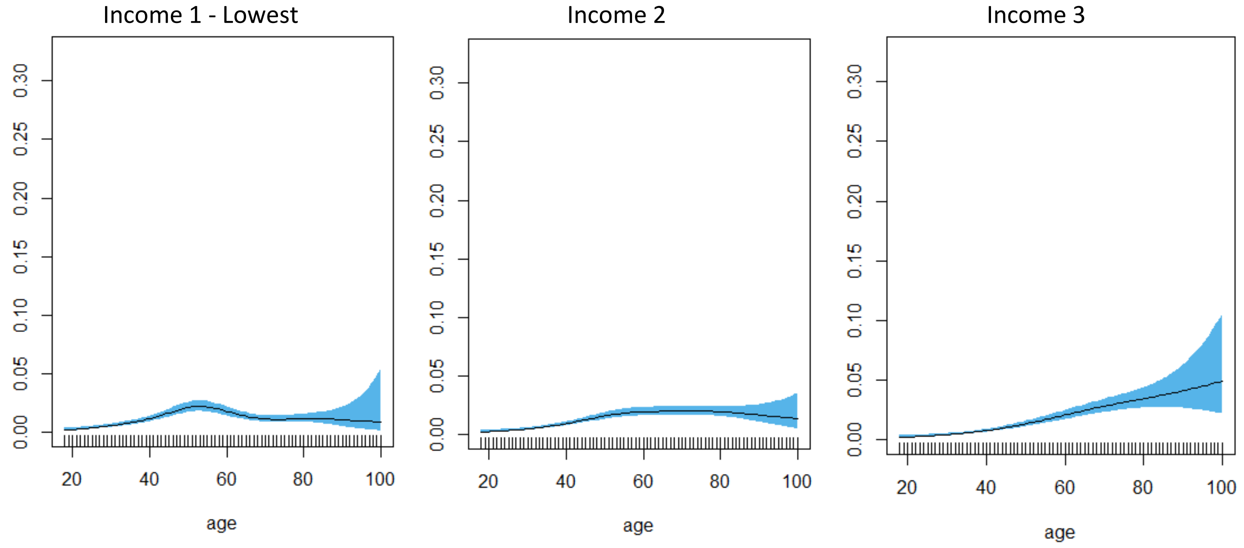

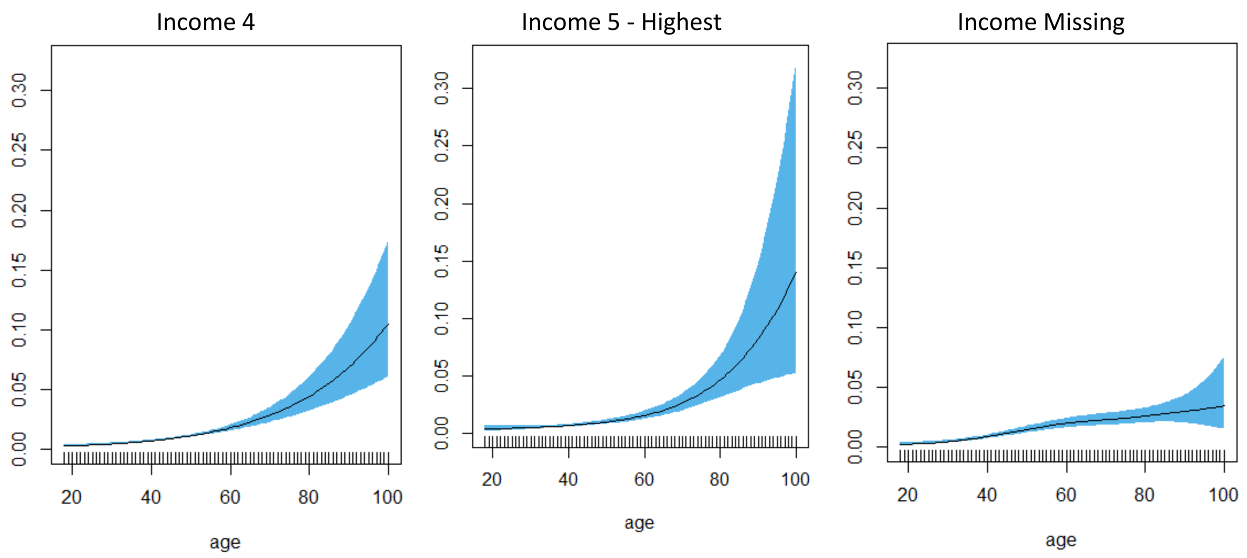
Income 4
Income 5 - Highest
Income Missing
0.00
0.05
0.10
0.15
0.20
0.25
0.30
20
40
60
80
100
age

Figure SM5: Non-parametric estimation of the effect of age on likelihood of reported opioid use by employment status in males

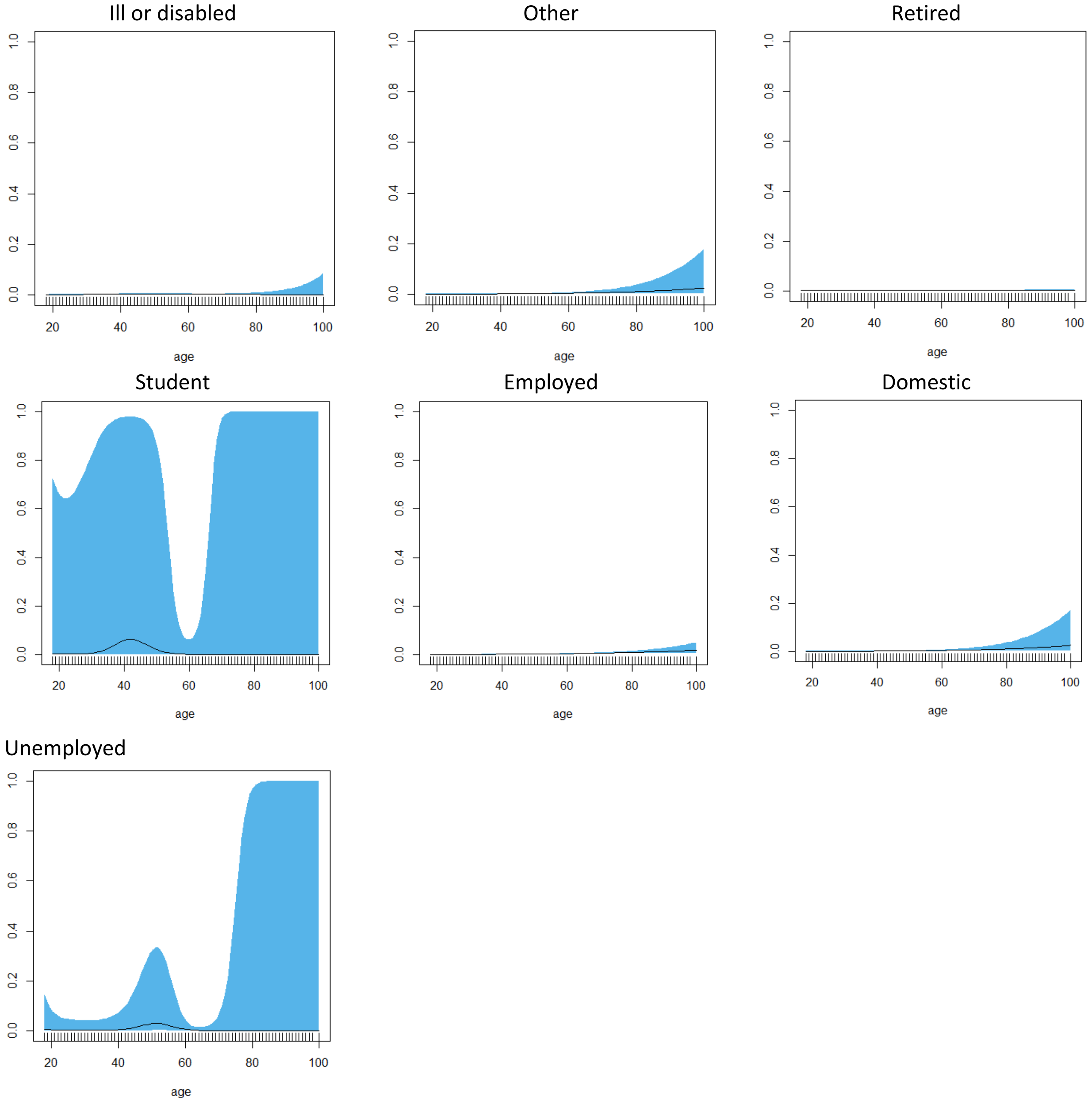

Figure SM6: Non-parametric estimation of the effect of age on likelihood of reported opioid use by income highest qualification in males

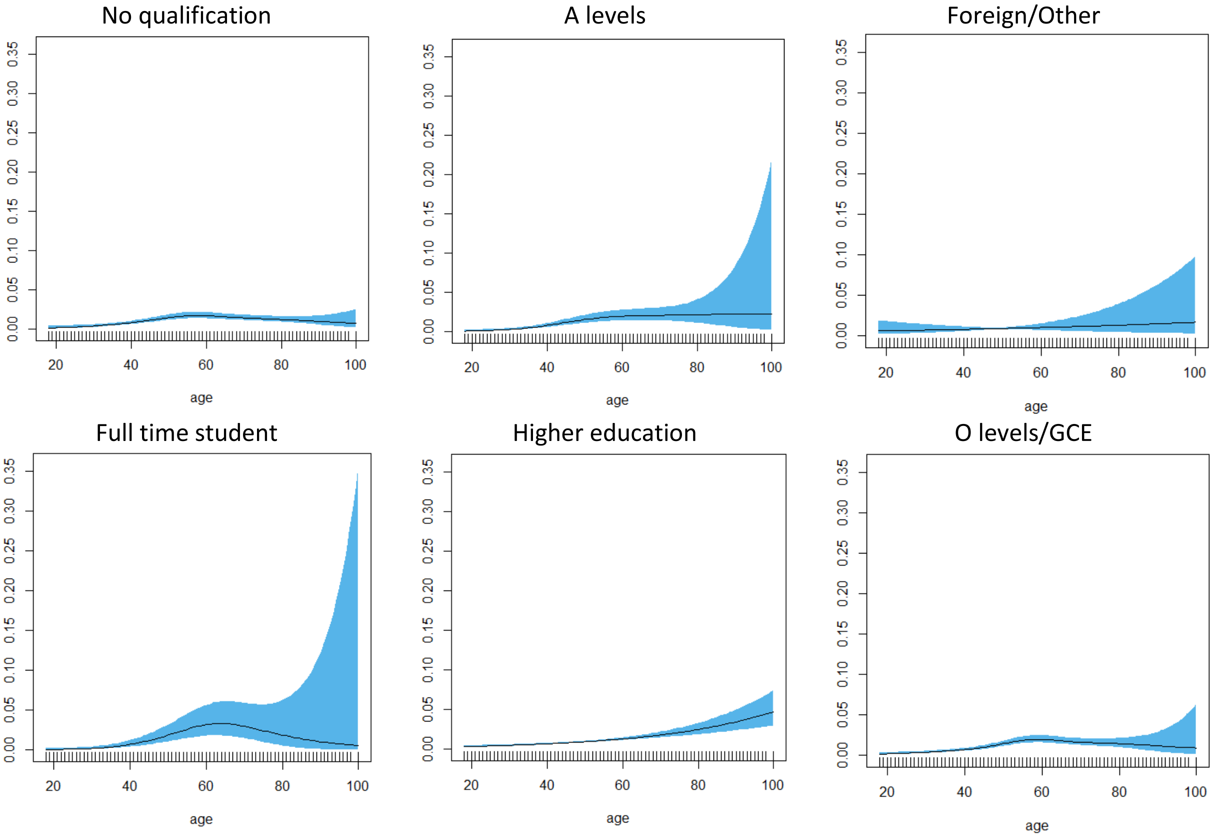

Supplement: Supplementary data [file bmjopen-2021-057428supp002.pdf]
