# Supplementary material for: Examining non-linearity in the association between age and reported opioid use in different socioeconomic strata: cohort study using Health Survey for England waves from 1997 to 2014
Source: BMJ Open. 2023 Mar 1;13(3):e057428. doi: 10.1136/bmjopen-2021-057428 (PMC9980331; doi:10.1136/bmjopen-2021-057428)
Supplement: Author's manuscript [file bmjopen-2021-057428.draft_revisions.pdf]

BMJ Open is committed to open peer review. As part of this commitment we make the peer review history of every article we publish publicly available.

When an article is published we post the peer reviewers' comments and the authors' responses online. We also post the versions of the paper that were used during peer review. These are the versions that the peer review comments apply to.

The versions of the paper that follow are the versions that were submitted during the peer review process. They are not the versions of record or the final published versions. They should not be cited or distributed as the published version of this manuscript.

BMJ Open is an open access journal and the full, final, typeset and author-corrected version of record of the manuscript is available on our site with no access controls, subscription charges or pay-per-view fees (http://bmjopen.bmj.com).

If you have any questions on BMJ Open's open peer review process please email info.bmjopen@bmj.com

The Nonlinear Association between Age and Reported Opioid Use and the Mediating Effect of Socioeconomic Status: Cohort Study Using Health Survey for England Waves from 1997 to 2014

Journal:	*BMJ Open*
Manuscript ID	bmjopen-2021-057428
Article Type:	Original research
Date Submitted by the Author:	26-Oct-2021
Complete List of Authors:	Nowakowska, Magdalena; The University of Manchester, Centre for Primary Care and Health Services Research<br>Zghebi, Salwa; The University of Manchester, Centre for Primary Care and Health Services Research<br>Chen, Li-chia; The University of Manchester, Division of Population Health, Health Services Research and Primary Care; School of Health Sciences; Faculty of Biology, Medicine and Health<br>Kontopantelis, Evangelos; The University of Manchester, Division of Informatics, Imagine and Data Sciences
Keywords:	EPIDEMIOLOGY, PAIN MANAGEMENT, SOCIAL MEDICINE

I, the Submitting Author has the right to grant and does grant on behalf of all authors of the Work (as defined in the below author licence), an exclusive licence and/or a non-exclusive licence for contributions from authors who are: i) UK Crown employees; ii) where BMJ has agreed a CC-BY licence shall apply, and/or iii) in accordance with the terms applicable for US Federal Government officers or employees acting as part of their official duties; on a worldwide, perpetual, irrevocable, royalty-free basis to BMJ Publishing Group Ltd ("BMJ") its licensees and where the relevant Journal is co-owned by BMJ to the co-owners of the Journal, to publish the Work in this journal and any other BMJ products and to exploit all rights, as set out in our licence.

The Submitting Author accepts and understands that any supply made under these terms is made by BMJ to the Submitting Author unless you are acting as an employee on behalf of your employer or a postgraduate student of an affiliated institution which is paying any applicable article publishing charge ("APC") for Open Access articles. Where the Submitting Author wishes to make the Work available on an Open Access basis (and intends to pay the relevant APC), the terms of reuse of such Open Access shall be governed by a Creative Commons licence – details of these licences and which Creative Commons licence will apply to this Work are set out in our licence referred to above.

Other than as permitted in any relevant BMJ Author's Self Archiving Policies, I confirm this Work has not been accepted for publication elsewhere, is not being considered for publication elsewhere and does not duplicate material already published. I confirm all authors consent to publication of this Work and authorise the granting of this licence.

For peer review only

The Nonlinear Association between Age and Reported Opioid Use and the Mediating Effect of Socioeconomic Status: Cohort Study Using Health Survey for England Waves from 1997 to 2014

Magdalena Nowakowska,[1,2,3] Salwa S Zghebi,[1,4] Li-Chia Chen,[5] Evangelos Kontopantelis,[1,2]

1. NIHR School for Primary Care Research; Centre for Primary Care and Health Services Research; Manchester Academic Health Science Centre (MAHSC), University of Manchester, M13 9PL, Manchester, UK
2. Division of Data Informatics, Imaging and Data Sciences; School of Health Sciences; Faculty of Biology, Medicine and Health; Manchester Academic Health Science Centre (MAHSC), University of Manchester, M13 9PL, Manchester, UK
3. NIHR Greater Manchester Patient Safety Translational Research Centre; University of Manchester, M13 9PL, Manchester, UK
4. Division of Population Health, Health Services Research and Primary Care; School of Health Sciences; Faculty of Biology, Medicine and Health; Manchester Academic Health Science Centre (MAHSC), University of Manchester, M13 9PL, Manchester, UK
5. Division of Pharmacy and Optometry; School of Health Sciences; Faculty of Biology, Medicine and Health; Manchester Academic Health Science Centre (MAHSC), University of Manchester, M13 9PL, Manchester, UK

Corresponding Author
Magdalena Nowakowska
Suite 5, 5th floor, Williamson Building,
176 Oxford Road, M13 9PL Manchester
Magdalena.nowakowska@manchester.ac.uk

For peer review only - http://bmjopen.bmj.com/site/about/guidelines.xhtml

Word count: 2,142
Number of tables: 2
Number of figures: 3

For peer review only

For peer review only - http://bmjopen.bmj.com/site/about/guidelines.xhtml

ABSTRACT

Background: Age and socioeconomic status (SES) predict several health-related outcomes, including prescription opioid use. Contrasting findings from previous literature found higher prevalence of opioid use in both people over 65 years old and the working-age population of 35-55 years old. This study aimed to analyse if the association between age and opioid use is nonlinear and differs in adults with different SES levels.

Methods: This cohort study utilised the Health Survey for England waves 1997 to 2014 data to investigate the shape of the correlation between reported opioid use and income decile, employment status, and educational level. A semi-parametric generalised additive model (GAM) was employed, so that linearity of correlation was not assumed. The shape of the relationship was assessed using the effective degrees of freedom (EDF).

Results: Positive correlation between age and reported opioid use, more linear in people in the highest income decile (EDF: 1.01M, $p<0.001$) and higher education (EDF: 2.03, $p<0.001$) was observed. In people on lower income and with lower levels of education, the probability of reported opioid use peaks was at around 40 to 60 years old and slowly decreased after that. Higher income decile and higher levels of education were predictors of a lower probability of reported opioid use (OR: 0.27, 95%CI: 0.21 to 0.36 and OR: 0.48, 95%CI: 0.41 to 0.57 respectively). There was no statistically significant difference in opioid use between employed and unemployed people.

Conclusion: More research is needed into exploring patterns in opioid use in older people, particularly from disadvantaged socioeconomic backgrounds.

For peer review only

For peer review only - http://bmjopen.bmj.com/site/about/guidelines.xhtml

Strengths and Limitations of this Study:

- The use of Generalised Additive Models to investigate the relationship between age and reported opioid use within different socioeconomic strata allowed exploration of the correlation without the assumption of linearity
- Due to limitations in recorded data, the distinction between type, strengths and duration of opioids was not accounted for
- Survey data used in this study is sensitive to self-reporting bias
- The low prevalence of opioid users limited the number of covariates used in the statistical model

INTRODUCTION

Opioid painkillers are effective analgesics that can provide crucially pain relief for patients with acute or cancer pain. However, extensive research suggests a dose and potency dependent association between opioid use and several adverse severe outcomes in patients who persistently use opioids.[1,2] Opioid use, as well as the associated adverse effects, varies across individuals and communities.[3,4] A growing body of academic literature focuses on the biological, psychosocial, and institutional factors that influence the likelihood of prescription opioid use. Socioeconomic status (SES), which can be defined through several measures, including income, occupational status, and education, was found to be closely associated with opioid prescribing[4] as well as female gender,[5] ethnicities,[6] and presence of psychological comorbidities.[7] Age is one of the strongest predictors of health and health outcomes, and the factor most commonly included in predictive models. However, the association between age and prescription opioid use is not necessarily linear.

Older people are more likely to experience complex health problems and often in need of multiple prescribed medications.[8] Several studies found that older people are more likely to use opioid analgesia.[9] This could be partially explained by the opioids' recommended indications in palliative care and the higher prevalence of painful conditions in older people.[10] In contrast, some studies observed a higher prevalence of opioid use in 35-49 years old.[11] Working-age adults may be more likely to be exposed to trauma and experience occupation-related pain. In a UK-based study, the highest proportion of new users of weak and moderate opioids were aged 35 to 54 years old, whereas over 50% of new users of strong opioids were 75 years and older.[12]

The relationship between age and opioid analgesia need is complex and influenced by many factors, including race, underlying mental health conditions and socioeconomic circumstances. A nearly 300% difference in opioid prescription prevalence was observed across the race/ethnicity-income gradient in the US.[13] In Sweden, the highest risk of opioid use was observed in medium-income women aged 65 years or older, living alone and with psychological distress; whereas the lowest risk was observed in low-income men aged 18-44, living alone and without psychological distress.[14] Socioeconomic status, including income, employment status and education level, can determine persons living and working conditions, access and interactions with the healthcare system, and overall health status, changing personal health and opioid-related life trajectories. This study hypothesises that the association between age and reported opioid use varies by SES level. This hypothesis is tested using generalised additive models, a regression analysis method that allows exploration of the non-linearity of the relationship between age and opioid use within groups of different income, employment status, and education levels.

For peer review only - http://bmjopen.bmj.com/site/about/guidelines.xhtml

METHODS

Data Source

This cohort study used the Health Survey for England (HSE), a national annual survey designed to monitor the nation's health and care trends.[15] Participants were offered a nurse visit during which several questions about medication use and measurements such as height and weight are recorded. Data on opioid use was available for extraction from surveys conducted between 1997 and 2014, and therefore, these data were analysed in this study. The HSE surveys are reviewed yearly by the Research Ethics Committee, and informed consent was obtained from all participants. All data used in this analysis is freely available.[16]

Study Population

Participants in the HSE were chosen using a random selection from all private households' addresses in England. Participants aged 18 and older with no reported diagnosis of cancer who completed the nurse interview part of the survey were included in the study. At a number of annual waves (1997-2014), a boost sample of a population with specific characteristics was collected. To minimise bias, we excluded the boost sample of people living in care homes which were included in the 2000 and 2005 year waves. The ethnic minority boost sample included at waves 1999 and 2004 was included in the analysis.

Definition of Opioid Use

Participants were asked if they take any prescribed medications and, if yes, permission was sought for a nurse to see its container and to record the name of the medication. Up to 22 prescribed medications were recorded using the British National Formulary (BNF) Codes. Medications coded as opioid analgesics (BNF code 40702) were included in this study. If at least on opioid analgesic was recorded, participant was coded as opioid users, and otherwise, they were coded as not an opioid users.

Socioeconomic Status Measures

Socioeconomic status was measured using three commonly used indicators: income, employment status, and education level. Income was measured using equalised income variable deciles from lowest to highest. This measure considers different financial resource requirements of different households by adjusting total household income for the number of adults and children in the household. Employment was categorised using participants' reported activity in the week before the interview. Education was categorised using participants' reported highest qualification achieved at the time of the interview. People with missing employment or education data were excluded from the main analysis due to small numbers (n=45; 0.04% and n=105; 0.08%, respectively). A larger number of people had missing income information (n=19,007; 15.2%); therefore, a separate category of "missing income" was included to investigate if they represent a different population.

Statistical Analysis

Descriptive statistics for people with and without reported opioid use were calculated. To analyse the effect of age on the likelihood of reported opioid use in groups of different socioeconomic status, a semi-parametric generalised additive model (GAM) was implemented in the following form:

$$\log\left(\frac{P(Y=1 \mid X=x)}{P(Y=0 \mid X=x)}\right) = \alpha + \beta X + f(age)(z_i)$$

In the parametric portion of the model, α is the intercept and β is the vector of parameters associated with a set of explanatory variables X. The results are presented in odds ratios (ORs) and the corresponding 95% confidence intervals (95%CI).

For peer review only - http://bmjopen.bmj.com/site/about/guidelines.xhtml

The non-parametric portion of the model is formed by the smoothing function f for age which can vary for each category of SES measure zi. GAM is an extension of the commonly used Generalised Linear Model (GLM), which relaxes the hypothesis that the relationship between predictor and outcome variable is linear.[17] Instead, the relationship is modelled using smooth functions which can take any shape. Varying the shape of the smooth function by category of SES allows better interpretation of the relationship between age and opioid use in groups with different income, employment status, and education levels. This relationship can be interpreted using the effective degrees of freedom (EDF) which can indicate whether the relationship is linear (EDF=1) or non-linear (EDF>1). Furthermore, graphical visualisation of the relationship is provided to aid the interpretation.

As a sensitivity analysis, all analyses were performed separately for females and males to investigate if different patterns emerge. These analyses showed no noticeable difference and are presented in the Supplementary Material (Tables SM1 and SM2, figures SM1-SM6). All analyses were performed using R version 3.6.3, and GAM estimations were performed using the *mgcv* package.[18]

Patient and Public Involvement

No patients were involved in the design, implementation or writing of this systematic review and meta-analysis. The results will be disseminated to the appropriate audience.

RESULTS

In total, 124,740 people met the inclusion criteria, with 2,470 (2.23%) reporting opioid use across all waves (Table 1). The mean age of opioid users was higher than non-users (59.00 ± 15.38 vs. 48.47 ± 17.75). A higher proportion of females and white people were opioid users than males and people of any other ethnicity (2.23% vs 1.67% and 2.13% vs. 0.86%, respectively). After adjusting for all covariates, in all models with the three SES indicators, females, white people, and people living in urban areas were more likely to report opioid use (Table 2).

Table 1: Characteristics of all eligible participants from HSE 1997-2014

	Opioid non-users	Opioid users
Number (%)	122,270 (98.02)	2,470 (1.98)
Age, mean (± SD)	48.47 (±17.75)	59.00 (±15.38)
Gender		
Female, N (%)	67,584 (97.77)	1,544 (2.23)
Male, N (%)	54,686 (98.33)	926 (1.67)
Ethnicity		
White	107,797 (97.87)	2,343 (2.13)
Non white	14,390 (99.14)	125 (0.86)
Income decile		
1 – lowest	10,300 (97.41)	274 (2.59)
2	10,288 (97.30)	286 (2.71)
3	10,218 (96.63)	356 (3.37)
4	10,272 (97.15)	301 (2.85)
5	10,342 (98.34)	231 (2.18)
6	10,398 (98.74)	175 (1.66)
7	10,440 (98.74)	133 (1.26)
8	10,475 (99.07)	98 (0.93)
9	10,461 (98.94)	112 (1.06)

For peer review only - http://bmjopen.bmj.com/site/about/guidelines.xhtml

For peer review only

10 - Highest	10,487 (99.19)	86 (0.81)
Income missing	18,589 (97.80)	418 (2.20)
Employment Status		
Student	4,123 (99.66)	14 (0.34)
Employed	69,564 (99.33)	465 (0.66)
Unemployed	2,367 (99.25)	18 (0.75)
Ill or disabled	4,726 (86.87)	714 (13.13)
Retired	29,055 (96.46)	1,067 (3.54)
Domestic worker	11,321 (98.56)	165 (1.44)
Other	1,071 (97.72)	25 (2.28)
Employment Missing	43 (95.55)	2 (4.44)
Education		
Higher Education	36,026 (98.79)	441 (1.21)
A levels	13,896 (98.71)	181 (1.29)
O levels/GSE	31,358 (98.16)	586 (1.83)
Foreign/other	3,624 (97.84)	80 (2.16)
No qualifications	31,238 (96.51)	1,130 (3.49)
Full time student	6,026 (99.19)	49 (0.81)
Education Missing	102 (97.14)	3 (2.86)

Compared to people in the lowest income decile, people reporting income in the highest decile were significantly less likely to report opioid use (OR: 0.27, 95%CI: 0.21 to 0.36). Compared to people who were employed the week before the interview, unemployed people were not statistically different in the likelihood of reporting opioid use. However, as expected, those classified as ill or disabled were significantly more likely to report opioid use (OR: 16.50, 95%CI: 13.24 to 20.55). People performing domestic work were also more likely to report opioid use (OR: 1.60, 95%CI: 1.32 to 1.94). Those with higher education were less likely to report opioid use than those with no qualifications (OR: 0.48, 95%CI: 0.41 to 0.57).

Table 2: Semi-parametric generalised additive models for the odds of reported prescription opioid use with smooth function for the effect of age, varying by SES measure

	**Opioid use (Model including income**	**Opioid use (Model including employment status)**	**Opioid use (Model including education)**
**Parametric coefficients**	**OR (95% CI)**	**OR (95% CI)**	**OR (95% CI)**
Intercept	0.009 (0.007 to 0.011) ***	0.003 (0.002 to 0.003) ***	0.01 (0.007 to 0.011) ***
Female (ref: Male)	1.32 (1.21 to 1.43) ***	1.53 (1.40 to 1.67) ***	1.33 (1.22 to 1.44) ***
White (ref: Not white)	2.24 (1.86 to 2.70) ***	2.01 (1.67 to 2.42)***	1.94 (1.61 to 2.32) ***
Urban (ref: Rural)	1.27 (1.15 to 1.41) ***	1.20 (1.08 to 1.33) ***	1.29 (1.17 to 1.43) ***
Income Decile (ref: 1-lowest)			
2	0.93 (0.76 to 1.13)		
3	1.01 (0.82 to 1.24)		
4	0.80 (0.65 to 0.99) *		
5	0.55 (0.43 to 0.70) ***		
6	0.51 (0.40 to 0.64) ***		
7	0.38 (0.29 to 0.49) ***		
8	0.34 (0.26 to 0.44) ***		
9	0.40 (0.32 to 0.51) ***		
10 – Highest	0.27 (0.21 to 0.36) ***		

For peer review only - http://bmjopen.bmj.com/site/about/guidelines.xhtml

Income Missing	0.64 (0.53 to 0.77) ***		
Economic status (ref: Employed)			
Student		0.92 (0.36 to 2.35)	
Unemployed		1.61 (0.97 to 2.64)	
Ill or disabled		16.50 (13.24 to 20.55) ***	
Retired		1.60 (0.93 to 2.76)	
Domestic worker		1.60 (1.32 to 1.94) ***	
Other		3.14 (2.02 to 4.87) ***	
Education (ref: No qualifications)			
Higher Education			0.48 (0.41 to 0.57) ***
A-levels			0.57 (0.47 to 0.70) ***
O-levels/GSE			0.71 (0.61 to 0.83) ***
Foreign/other			0.63 (0.43 to 0.91) *
Student			0.57 (0.44 to 0.82) **
**Non Parametric**	**EDF**	**EDF**	**EDF**
Income Decile:			
1 – Lowest	3.97 ***		
2	6.08 ***		
3	4.17 ***		
4	3.31 ***		
5	2.64 ***		
6	2.51 ***		
7	2.06 ***		
8	1.52 ***		
9	1.04 ***		
10 – Highest	1.01 ***		
Income Missing	3.30 ***		
Economic status:			
Employed		1.01 ***	
Student		1.002	
Unemployed		1.002 *	
Ill or disabled		3.12 ***	
Retired		4.99 **	
Domestic worker		2.74 ***	
Other		1.76 *	
Education:			
Higher Education			2.03 ***
A-levels			2.93 ***
O-levels/GSE			3.30 ***
No qualifications			4.30 ***
Foreign/Other			1.01 **
Student			2.81 ***

OR – Odds Ratio; CI: Confidence Intervals; EDF - Estimated Degrees of Freedom
** p-value < 0.05*
*** p-value < 0.01*
**** p-value <0.001*

The effect of age on the likelihood of reporting opioid use was non-linear and significant in all income deciles as suggested by the statistically significant EDF. The higher the income decile, the relationship between age and probability of opioid use approached linear relationship with the EDF: 1.01, p<0.001

For peer review only - http://bmjopen.bmj.com/site/about/guidelines.xhtml

for highest income decile and EDF: 3.97, p<0.001 for lowest income decile. This suggests that in people with higher income, older people were more likely to report opioid use. However, in lower-income deciles, the relationship was non-linear. In particular, in people on the lowest income decile, the probability of opioid use increased with age until approximately 55 years old, after which it reduced (Figure 1).

Concerning employment status, age had a statistically significant association with reported opioid use in people who were employed (EDF: 1.01, p<0.001), ill or disabled (EDF: 3.12, p<0.001), domestic workers (EDF: 2.74, p<0.001), and with lower significance levels, retired (EDF: 4.99, p<0.01), unemployed (EDF: 1.002, p<0.05), and other economic statuses (EDF: 1.76, p<0.05). In people who were employed and unemployed, the relationship approached linearity (EDF: 1.01, p<0.001 and EDF: 1.002, p<0.05 respectively), whereas in people who were ill or disabled, the distribution was slightly skewed towards people between 40 and 60 years old, and in domestic workers, the likelihood peaked around 60 years old (Figure 2).

For all levels of education status, the effect of age on the odds of reporting opioid use was statistically significant. The effect was almost linear for people with foreign/other qualifications (Figure 3). In people with higher education, older age was associated with higher odds of reported opioid use. In contrast, in people with lower qualifications (A-levels, O-levels/GSE) the odds of reported opioid use peaked around 60 years old slightly decrease after that. The odds of reported opioid use peaked earlier in people with no qualifications at around 50 years old.

DISCUSSION

This study found that the relationship between age and the likelihood of reported opioid use changes with different measures of socioeconomic status. In people with higher income and education, the relationship is more linear, increasing with age. In people on lower income with lower levels of education, the likelihood of reported opioid use peaks at around 40 to 60 years old and slowly decreases after that. Consistently with previous research, females, white people, those living in urban areas, and those with lower levels of SES were more likely to report opioid use. The difference between employed and unemployed people was not statistically significant regarding the likelihood of reported opioid use. In both groups, the association with age was linear and increasing with age.

Existing studies found mixed results regarding the correlation between age and use of opioids; some studies found a higher prevalence in older populations[9] whereas others in the working-age population.[11] This study shows that SES may contribute to the non-linear relationship between age and opioid use. Shaw, Roelofs, and Punnett (2017)[19] suggest two pathways in which working conditions, strongly correlated with income, related to opioid use. The first suggests that physical injury at work or cumulative trauma from strenuous labour leads to pain and the use of painkillers. The second relates to psychological stress from unmanageable job demands like time pressure or economic insecurity and job instability, leading to depression and anxiety, which may correlate with chronic opioid use. These adverse working conditions are expected in people with lower-paying jobs, explaining the observed higher prevalence of reported opioid use in working-age people with lower SES. Furthermore, people experiencing disability or illness who are in a greater need of opioid treatment are more likely to be on lower income due to difficulties sustaining employment, regardless of age.

For peer review only - http://bmjopen.bmj.com/site/about/guidelines.xhtml

Our findings show an increase in reported opioid use with age in people with higher income and higher education. Reported opioid use in people with lower income and no higher education decreased after it peaked around 40 to 60 years old. Two possible explanations have been identified in the literature. Firstly, previous research found that the prevalence of musculoskeletal pain increases up to approximately 65 years old, after which it declines.[20] This has been linked to the decline of the adverse physical and mental effects of the workplace at the age of retirement and the lower prevalence of several diseases in surviving populations as people with chronic diseases tend to die early.[21] Furthermore, a review of pain management in the elderly showed that pain in the elderly might be untreated and misdiagnosed.[22] Conversely, a higher prevalence of opioid use would be expected in older people due to more complex health needs and the use of opioid analgesia in palliative care. Further research is needed to establish if older people from lower socioeconomic backgrounds are more likely to experience difficulties accessing adequate analgesia.

Strengths and limitations

The statistical techniques used in this study allowed modelling the relationship between age and odds of reported opioid use in a non-linear manner and present results separately for people with different levels of SES. Various methods can examine the interaction between two variables, each with different strengths and limitations. A common approach includes an interaction term where the effect of a covariate of interest can vary depending on another variable.[23] Alternatively, multilevel models allow parameters of interest, including the intercept and the slope of the regression line, to vary across more than one level.[24] However, these methods assume a linear relationship. Age can be modelled as categorical, but this can lead to complex models where multiple age categories interact with multiple SES categories, and information is difficult to interpret. Other techniques, including several machine learning methods, can produce highly accurate but difficult to interpret estimations. This study employed generalised additive models, a regression analysis method that allows exploration of the non-linearity of the relationship between age and opioid use within groups of different income, employment status, and education levels. To our knowledge, this is the first study to employ this technique to explore the relationship between age and prescription opioid use in England.

However, some limitations should be acknowledged. Firstly, the relationship between age and opioid use may be specific to different opioid strengths and durations. The data records in HSE did not capture the type of prescribed opioids; hence they did not adjust for the opioid type strength in the analysis. Secondly, opioid use was assessed based on self-reported data, which may be susceptible to recall and social desirability bias. However, this risk of bias was reduced by the involvement of qualified nurses in collecting medication data. Thirdly, the low prevalence of opioid use meant that a limited number of covariates was included in the model. Some variables, such as the presence of comorbidities, had to be excluded from the models.

Conclusion

Findings from this study suggest that although the relationship between age and odds of opioid use in people with higher income and education approaches linearity, in people with lower SES, the probability of exposure peaks between 40 to 60 years old and decreases after that. More research is needed into exploring patterns in opioid use in older people, particularly from disadvantaged socioeconomic backgrounds.

For peer review only - http://bmjopen.bmj.com/site/about/guidelines.xhtml

Authors contributions

All authors contributed to the design of the study. MN prepared and analysed the data. MN wrote the manuscript and MN, SSZ, LCC, and EK all critically edited the manuscript. All authors approved the manuscript before submission.

Competing interest

MN, SSZ, LCC, and EK have no conflict of interest to declare.

Role of the funding source

This study was funded as part of a PhD studentship from the National Institute for Health Research (NIHR) School for Primary Care Research (SPCR) and NIHR Greater Manchester Patient Safety Translational Research Centre. The study represents independent research by the NIHR. The views expressed in this publication are those of the authors and not necessarily those of the National Health Service, the NIHR or the Department of Health and Social Care. The study funders had no role in the study design, data collection, analysis or interpretation, in the writing of the paper or in the decision to submit the paper for publication. MN had full access to all the data in the study and had final responsibility for the decision to submit for publication.

Data sharing

All data used in this study are publicly available from the sources cited in the manuscript.

Research Ethics Approval

This study involves analysis of anonymous and publicly available data. No ethical approval was sought or required for this study.

For peer review only

For peer review only - http://bmjopen.bmj.com/site/about/guidelines.xhtml

Figure 1: Non-parametric estimation of the effect of age on likelihood of reported opioid use by income decile

Figure 2: Non-parametric estimation of the effect of age on likelihood of reported opioid use by economic status

Figure 3: Non-parametric estimation of the effect of age on likelihood of reported opioid use by education status

For peer review only

For peer review only - http://bmjopen.bmj.com/site/about/guidelines.xhtml

REFERENCES

1 Benyamin R, Trescot AM, Datta S, *et al.* Opioid complications and side effects. 2008. doi:10.36076/ppj.2008/11/s105

2 Moore RA, McQuay HJ. Prevalence of opioid adverse events in chronic non-malignant pain: systematic review of randomised trials of oral opioids. Arthritis Res. Ther. 2005;**7**:1–6. doi:10.1186/ar1782

3 Nowakowska M, Zghebi SS, Perisi R, *et al.* Association of socioeconomic deprivation with opioid prescribing in primary care in England: A spatial analysis. *J Epidemiol Community Health* 2021;**75**:128–36. doi:10.1136/jech-2020-214676

4 Macfarlane GJ, Beasley M, Jones GT, *et al.* The epidemiology of regular opioid use and its association with mortality: Prospective cohort study of 466 486 UK biobank participants. *EClinicalMedicine* 2020;**21**:100321. doi:10.1016/j.eclinm.2020.100321

5 Serdarevic M, Striley CW, Cottler LB. Sex differences in prescription opioid use. Curr. Opin. Psychiatry. 2017;**30**:238–46. doi:10.1097/YCO.0000000000000337

6 Ringwalt C, Roberts AW, Gugelmann H, *et al.* Racial Disparities Across Provider Specialties in Opioid Prescriptions Dispensed to Medicaid Beneficiaries with Chronic Noncancer Pain. *Pain Med (United States)* 2015;**16**:633–40. doi:10.1111/pme.12555

7 Davis MA, Lin LA, Liu H, *et al.* Prescription opioid use among adults with mental health disorders in the United States. *J Am Board Fam Med* 2017;**30**:407–17. doi:10.3122/jabfm.2017.04.170112

8 Hajjar ER, Cafiero AC, Hanlon JT. Polypharmacy in elderly patients. *Am J Geriatr Pharmacother* 2007;**5**:345–51. doi:10.1016/j.amjopharm.2007.12.002

9 Bedene A, Lijfering WM, Niesters M, *et al.* Opioid Prescription Patterns and Risk Factors Associated With Opioid Use in the Netherlands. *JAMA Netw open* 2019;**2**:e1910223. doi:10.1001/jamanetworkopen.2019.10223

10 Gibson SJ, Lussier D. Prevalence and Relevance of Pain in Older Persons. Pain Med. 2012;**13**:S23–6. doi:10.1111/j.1526-4637.2012.01349.x

11 Rasu RS, Knell ME. Determinants of opioid prescribing for nonmalignant chronic pain in US outpatient settings. *Pain Med (United States)* 2018;**19**:524–32. doi:10.1093/pm/pnx025

12 Jani M, Yimer BB, Sheppard T, *et al.* Time trends and prescribing patterns of opioid drugs in UK primary care patients with non-cancer pain: A retrospective cohort study. *PLoS Med* 2020;**17**:e1003270. doi:10.1371/journal.pmed.1003270

13 Friedman J, Kim D, Schneberk T, *et al.* Assessment of Racial/Ethnic and Income Disparities in the Prescription of Opioids and Other Controlled Medications in California. *JAMA Intern Med* 2019;**179**:469–76. doi:10.1001/jamainternmed.2018.6721

14 Persmark A, Wemrell M, Zettermark S, *et al.* Precision public health: Mapping socioeconomic disparities in opioid dispensations at Swedish pharmacies by Multilevel Analysis of Individual Heterogeneity and Discriminatory Accuracy (MAIHDA). *PLoS One* 2019;**14**:e0224008. doi:10.1371/journal.pone.0220322

15 Mindell J, Biddulph JP, Hirani V, *et al.* Cohort profile: The health survey for england. *Int J Epidemiol* 2012;**41**:1585–93. doi:10.1093/ije/dyr199

16 NHS Digital. Health Survey for England - NHS Digital. https://digital.nhs.uk/data-and-information/publications/statistical/health-survey-for-england (accessed 14 Jun 2021).

17 Hastie T, Tibshirani R. Generalized additive models for medical research.

18 Wood Simon. Mixed GAM Computation Vehicle with Automatic Smoothness Estimation. 2021. doi:10.1201/9781315370279

19 Shaw WS, Roelofs C, Punnett L. Work environment factors and prevention of opioid-related deaths. *Am J Public Health* 2020;**110**:1235–41. doi:10.2105/AJPH.2020.305716

20 Parsons S, Breen A, Foster NE, *et al.* Prevalence and comparative troublesomeness by age of musculoskeletal pain in different body locations. *Fam Pract* 2007;**24**:308–16.

For peer review only - http://bmjopen.bmj.com/site/about/guidelines.xhtml

doi:10.1093/fampra/cmm027
21 Cimmino MA, Ferrone C, Cutolo M. Epidemiology of chronic musculoskeletal pain. 2011.
22 Noroozian M, Raeesi S, Hashemi R, *et al.* Pain: The neglect issue in old people's life. *Open Access Maced J Med Sci* 2018;**6**:1773–8. doi:10.3889/oamjms.2018.335
23 Kontopantelis E, Sperrin M, Mamas MA, *et al.* Investigating heterogeneity of effects and associations using interaction terms. J. Clin. Epidemiol. 2018;**93**:79–83. doi:10.1016/j.jclinepi.2017.09.012
24 Peugh JL. A practical guide to multilevel modeling. *J Sch Psychol* 2010;**48**:85–112. doi:10.1016/j.jsp.2009.09.002

For peer review only

For peer review only - http://bmjopen.bmj.com/site/about/guidelines.xhtml

Figure 1: Non-parametric estimation of the effect of age on likelihood of reported opioid use by income decile

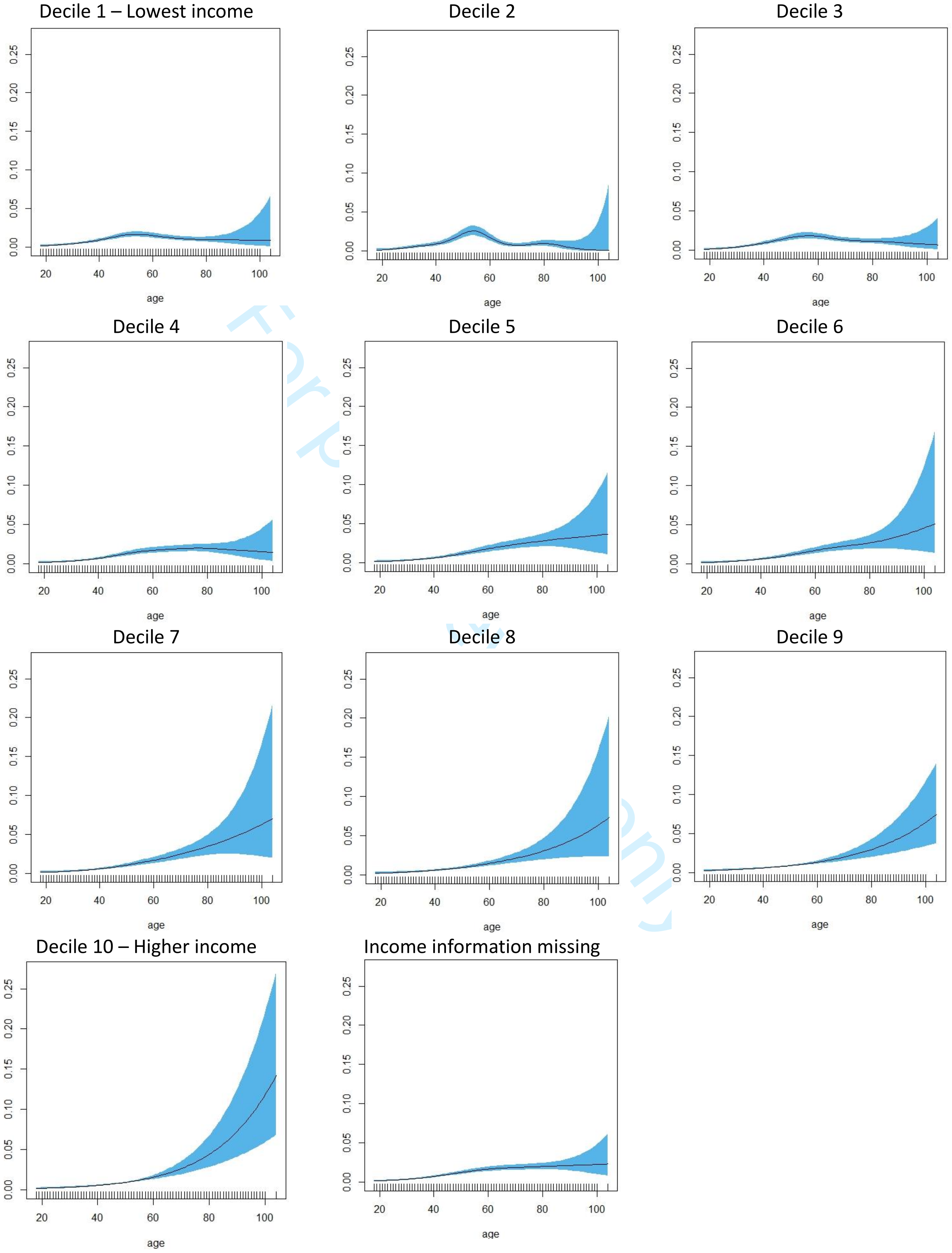

Figure 2: Non-parametric estimation of the effect of age on likelihood of reported opioid use by economic status

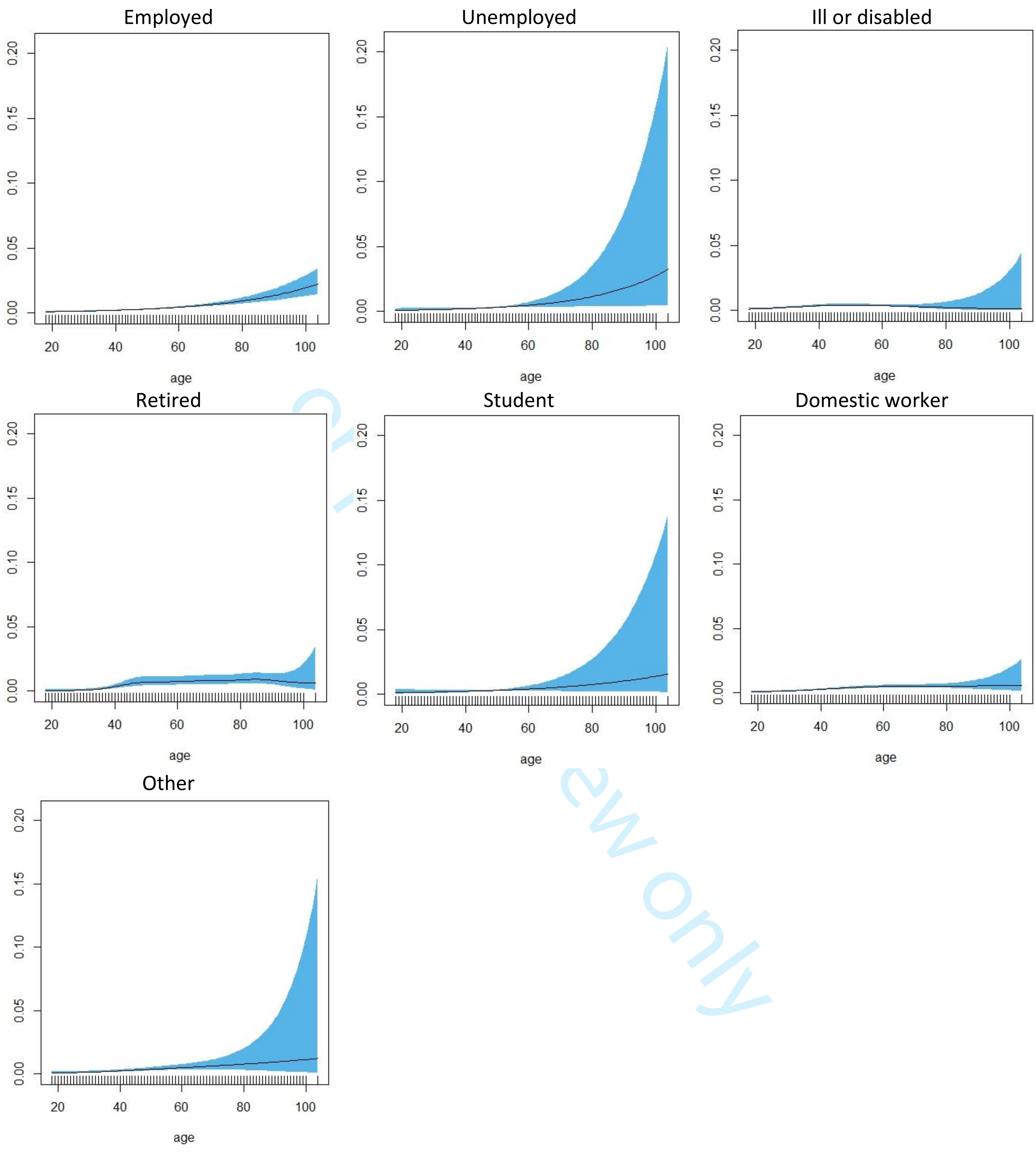

For peer review only - http://bmjopen.bmj.com/site/about/guidelines.xhtml

Figure 3: Non-parametric estimation of the effect of age on likelihood of reported opioid use by education status

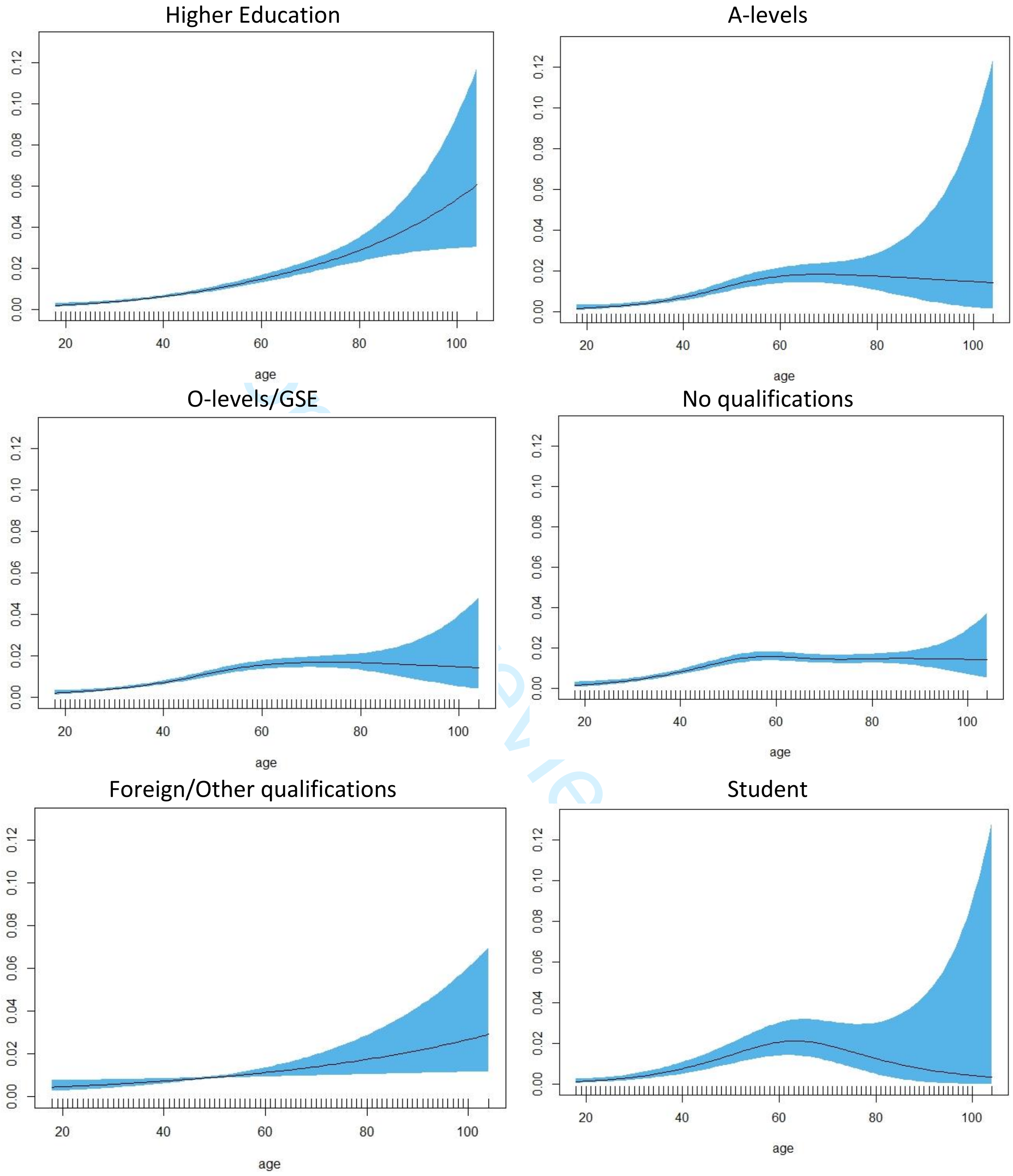

The nonlinear association between age and socioeconomic status on reported opioid use: cohort study using health survey for England waves from 1997 to 2014

Supplementary Materials: Sensitivity Analysis

For peer review only

Table SM1: Semi-parametric generalised additive models for the odds of reported prescription opioid use with smooth function for the effect of age, varying by SES measure in Females only

	Opioid use (Model including income	Opioid use (Model including employment status)	Opioid use (Model including education)
**Parametric coefficients**	**OR (95% CI)**	**OR (95% CI)**	**OR (95% CI)**
Intercept	0.01 (0.01:0.01) ***	0.004 (0.003:0.006) ***	0.012 (0.009:0.159) ***
White (ref: Not white)	2.11 (1.66:2.67) ***	1.91 (1.51:2.43)***	1.90 (1.50:2.41)***
Urban (ref: Rural)	1.30 (1.14:1.48) ***	1.23 (1.08:1.40)**	1.31 (1.14:1.49)***
Income Quantile (ref: 1-Lowest)			
Income 2	0.98 (0.81:1.19)		
Income 3	0.71 (0.57:0.87) ***		
Income 4	0.47 (0.38:0.59) ***		
Income 5 - Highest	0.41 (0.32:0.52) ***		
Income Missing	0.75 (0.60:0.93) **		
Economic Status (ref: Employed)			
Domestic		1.59 (1.29:1.97) ***	
Ill or disabled		17.52 ***(12.97:23.66)	
Other		3.04 (1.70:5.43)***	
Retired		1.75 (1.70:5.43)*	
Student		0.75 (0.19:2.80)	
Unemployed		1.78 (0.83:3.81)	
Education (ref: No qualification)			
Higher education			0.51 (0.41:0.62)***
A-levels			0.53 (0.41:0.69) ***
O-levels/GSE			0.71 (0.58:0.85)***
Foreign/Other			0.61 (0.39:0.94) *
Student			0.48 (0.31:0.75)**
**Non Parametric**	**EDF**	**EDF**	**EDF**
**Income Quantile**			
Income 1 - lowest	4.91 ***		
Income 2	3.52 ***		
Income 3	2.56 ***		
Income 4	1.12 ***		
Income 5 - highest	1.91 ***		
Income Missing	3.01 ***		

Employment status			
Domestic		2.669***	
Ill or disabled		2.626 *	
Other		1.428	
Retired		4.013 ***	
Student		1.000	
Unemployed		1.002	
Employed		1.003 ***	
Education			
Higher education			2.108***
A-levels			2.646***
O-levels/GSE			2.302***
No qualifications			3.631***
Foreign/Other			1.049*
FT Student			2.062*

Table SM2: Semi-parametric generalised additive models for the odds of reported prescription opioid use with smooth function for the effect of age, varying by SES measure in Males only

	**Opioid use (Model including income**	**Opioid use (Model including employment status)**	**Opioid use (Model including education)**
**Parametric coefficients**	**OR (95% CI)**	**OR (95% CI)**	**OR (95% CI)**
Intercept	0.01 (0.01:0.01) ***	0.003 (0.002:0.004)***	0.009 (0.006:0.13)***
White (ref: Not white)	2.48 (1.84:3.35) ***	2.17 (1.60:2.93)***	2.02***
Urban (ref: Rural)	1.24 (1.05:1.46) **	1.13 (0.96:1.34)	1.26**
Income Quintile (ref: 1-Lowest)	ref		
Income 2	0.85 (0.66:1.09)		
Income 3	0.34 (0.25:0.47) ***		
Income 4	0.24 (0.17:0.34) ***		
Income 5 - highest	0.30 (0.22:0.40) ***		
Income Missing	0.54 (0.41:0.71) ***		
Economic Status (ref: Employed))			
Domestic		2.82 (1.40:5.66)**	
Ill or disabled		15.51 (11.40:21.10)***	
Other		3.57 (1.89:6.74)***	
Retired		3.80 (2.66:5.43)****	
Student		0.12 (0.0002:89.32)	
Unemployed		0.33 (0.02:4.08)	
Education (ref: No qualification)			
Higher education			0.44 (0.34:0.57)***
A-levels			0.56 (0.40:0.79)***
O-levels/GSE			0.70 (0.54:0.91)**
Foreign/Other			0.65 (0.31:1.39)
Student			0.63 (0.39:1.06)
**Non Parametric**	**EDF**	**EDF**	**EDF**
**Income Quantile**			
Income 1 - lowest	4.73 ***		
Income 2	3.89 ***		
Income 3	2.69 ***		
Income 4	2.79 ***		
Income 5 - highest	1.13 ***		
Income Missing	2.89 ***		

For peer review only

Employment status			
Employed		1.300***	
Domestic		1.001*	
Ill or disabled		2.900**	
Other		1.002*	
Retired		1.002	
Student		2.742	
Unemployed		3.232	
Education			
Higher education			1.002***
A-levels			2.878***
O-levels/GSE			3.929***
No qualifications			3.695***
Foreign/Other			1.002
Student			2.741***

Figure SM1: Non-parametric estimation of the effect of age on likelihood of reported opioid use by income quantile in females

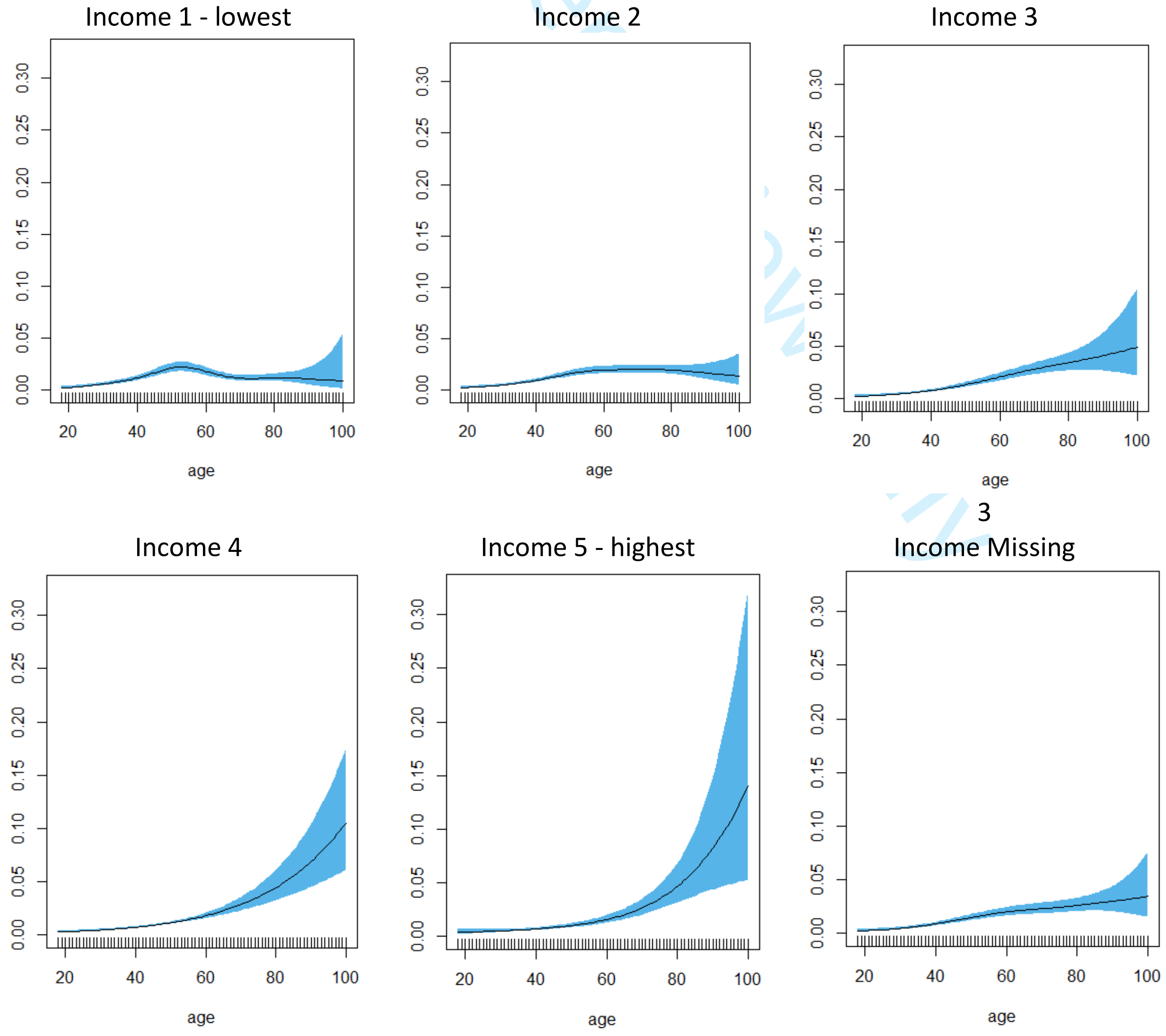

3

Figure SM2: Non-parametric estimation of the effect of age on likelihood of reported opioid use by employment status in females

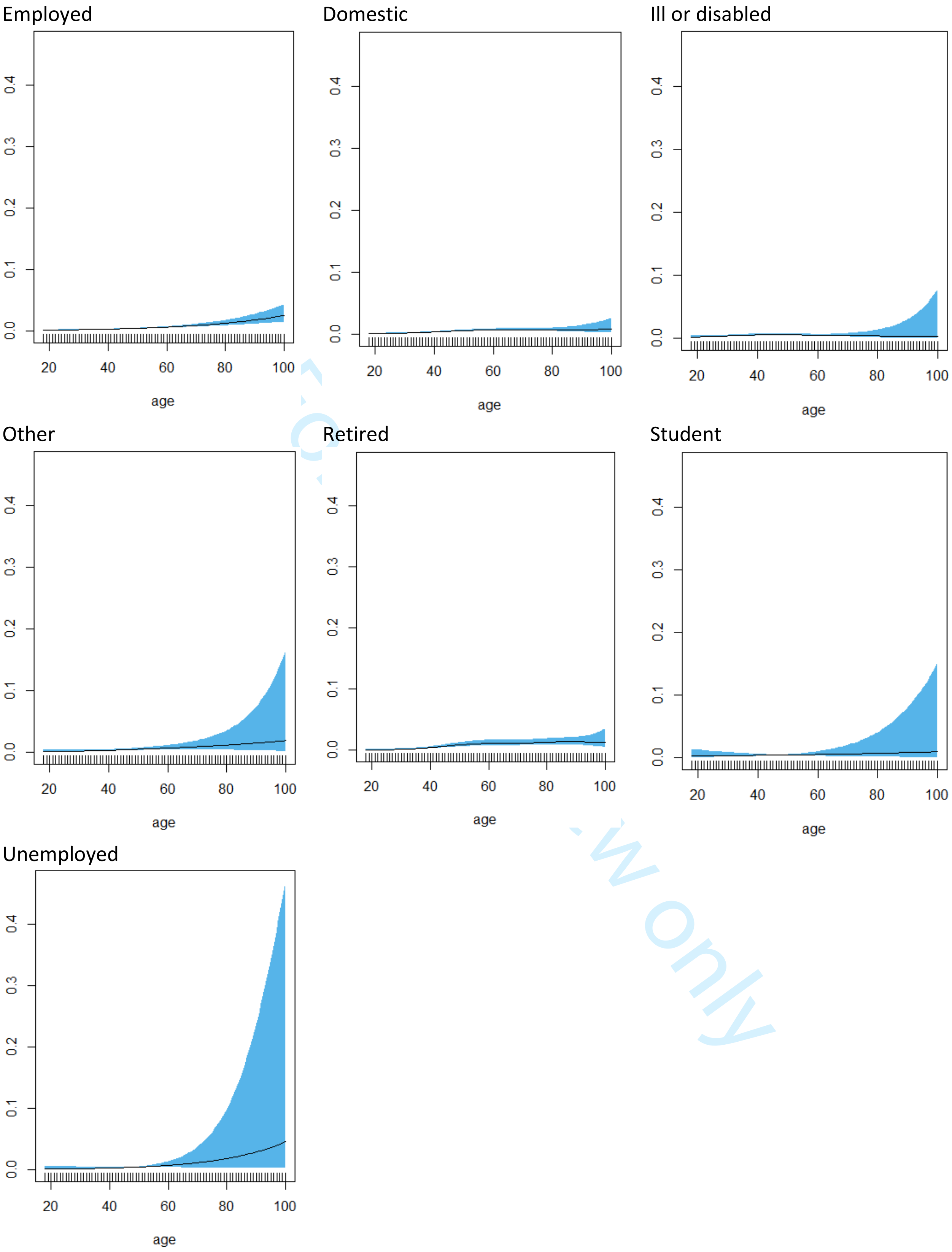

Figure SM3: Non-parametric estimation of the effect of age on likelihood of reported opioid use by income highest qualification in females

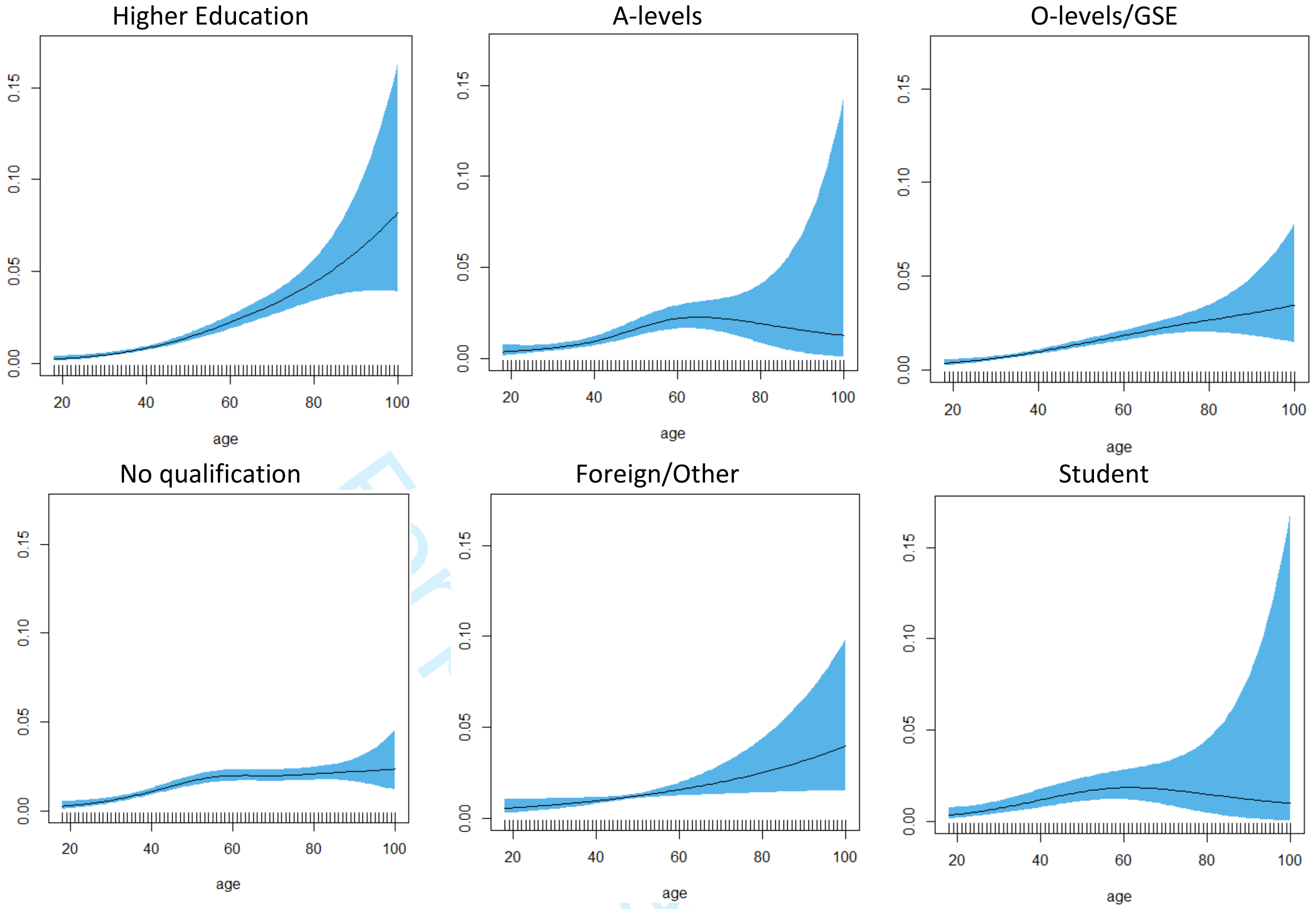

Figure SM4: Non-parametric estimation of the effect of age on likelihood of reported opioid use by income quantile in males

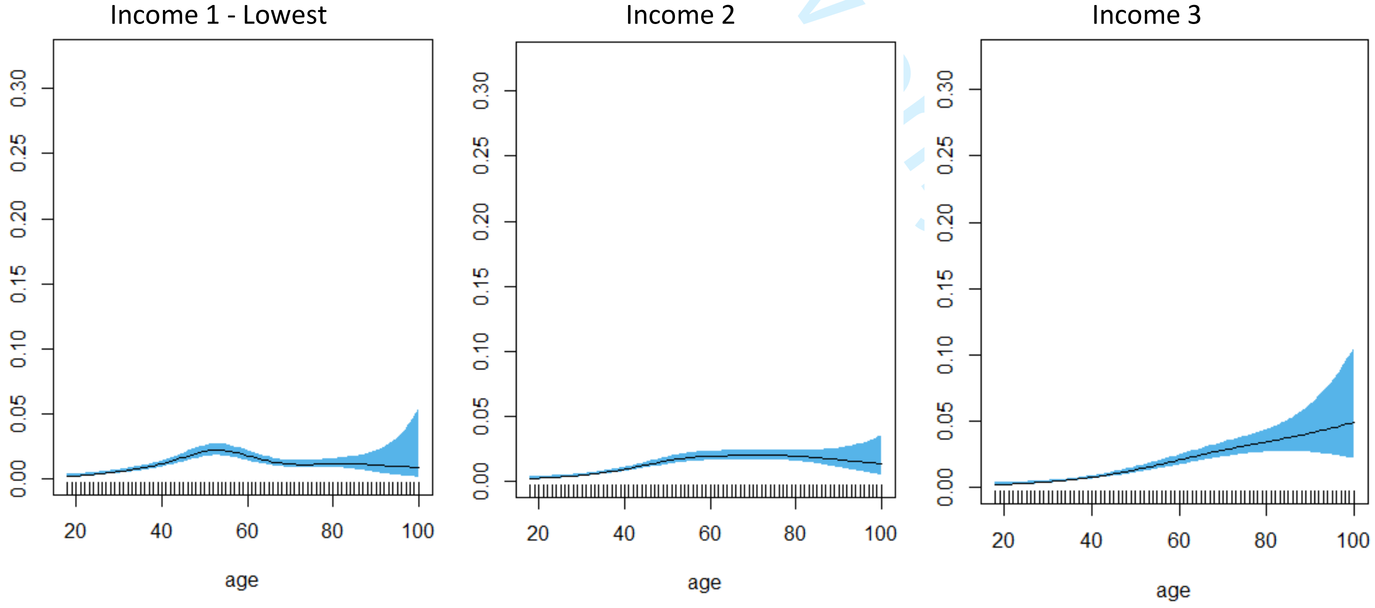

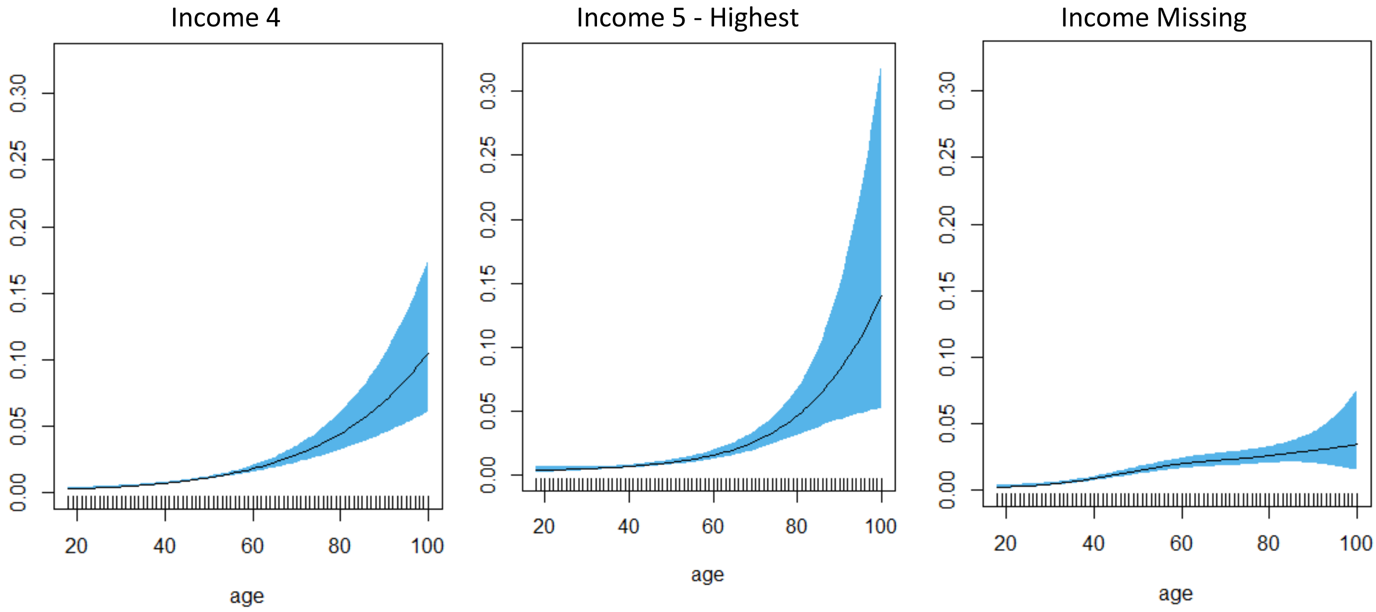

For peer review only

Figure SM5: Non-parametric estimation of the effect of age on likelihood of reported opioid use by employment status in males

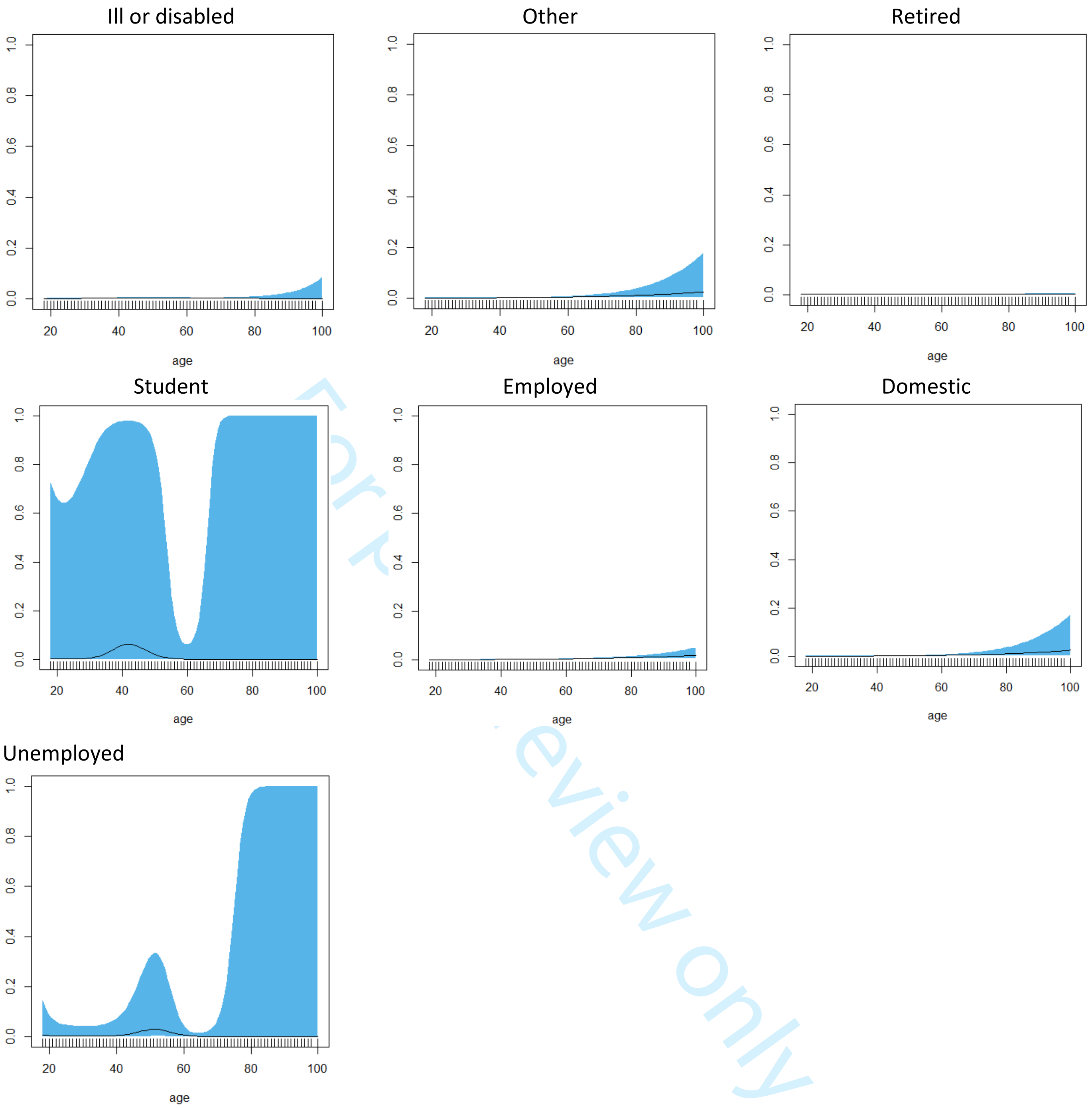

For peer review only

Figure SM6: Non-parametric estimation of the effect of age on likelihood of reported opioid use by income highest qualification in males

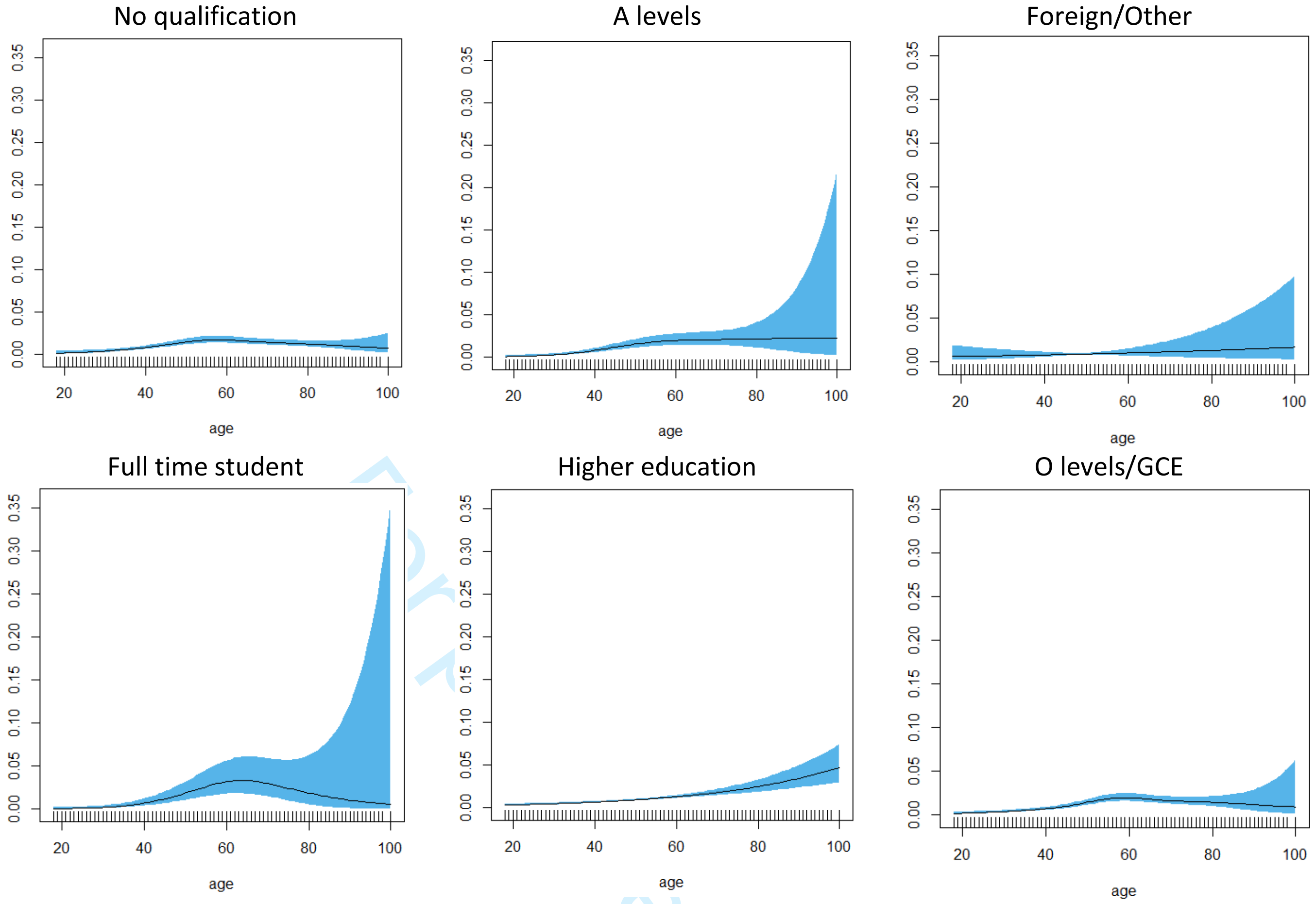

For Review only

STROBE Statement—Checklist of items that should be included in reports of ***cohort studies***

	Item No	Recommendation	Page No
**Title and abstract**	1	(*a*) Indicate the study's design with a commonly used term in the title or the abstract	2
		(*b*) Provide in the abstract an informative and balanced summary of what was done and what was found	2
**Introduction**			
Background/rationale	2	Explain the scientific background and rationale for the investigation being reported	3
Objectives	3	State specific objectives, including any prespecified hypotheses	3-4
**Methods**			
Study design	4	Present key elements of study design early in the paper	4
Setting	5	Describe the setting, locations, and relevant dates, including periods of recruitment, exposure, follow-up, and data collection	4
Participants	6	(*a*) Give the eligibility criteria, and the sources and methods of selection of participants. Describe methods of follow-up	4
		(*b*) For matched studies, give matching criteria and number of exposed and unexposed	NA
Variables	7	Clearly define all outcomes, exposures, predictors, potential confounders, and effect modifiers. Give diagnostic criteria, if applicable	4
Data sources/ measurement	8*	For each variable of interest, give sources of data and details of methods of assessment (measurement). Describe comparability of assessment methods if there is more than one group	4
Bias	9	Describe any efforts to address potential sources of bias	4-5
Study size	10	Explain how the study size was arrived at	4 and 6
Quantitative variables	11	Explain how quantitative variables were handled in the analyses. If applicable, describe which groupings were chosen and why	4-5
Statistical methods	12	(*a*) Describe all statistical methods, including those used to control for confounding	5
		(*b*) Describe any methods used to examine subgroups and interactions	5
		(*c*) Explain how missing data were addressed	4
		(*d*) If applicable, explain how loss to follow-up was addressed	NA
		(*e*) Describe any sensitivity analyses	5
**Results**			
Participants	13*	(a) Report numbers of individuals at each stage of study—eg numbers potentially eligible, examined for eligibility, confirmed eligible, included in the study, completing follow-up, and analysed	5
		(b) Give reasons for non-participation at each stage	4-5
		(c) Consider use of a flow diagram	NA
Descriptive data	14*	(a) Give characteristics of study participants (eg demographic, clinical, social) and information on exposures and potential confounders	5-6
		(b) Indicate number of participants with missing data for each variable of interest	4-6
		(c) Summarise follow-up time (eg, average and total amount)	NA
Outcome data	15*	Report numbers of outcome events or summary measures over time	5

For peer review only - http://bmjopen.bmj.com/site/about/guidelines.xhtml

Main results	16	(*a*) Give unadjusted estimates and, if applicable, confounder-adjusted estimates and their precision (eg, 95% confidence interval). Make clear which confounders were adjusted for and why they were included	6-7
		(*b*) Report category boundaries when continuous variables were categorized	4
		(*c*) If relevant, consider translating estimates of relative risk into absolute risk for a meaningful time period	NA
Other analyses	17	Report other analyses done—eg analyses of subgroups and interactions, and sensitivity analyses	SM
**Discussion**			
Key results	18	Summarise key results with reference to study objectives	8-9
Limitations	19	Discuss limitations of the study, taking into account sources of potential bias or imprecision. Discuss both direction and magnitude of any potential bias	9
Interpretation	20	Give a cautious overall interpretation of results considering objectives, limitations, multiplicity of analyses, results from similar studies, and other relevant evidence	9
Generalisability	21	Discuss the generalisability (external validity) of the study results	9
**Other information**			
Funding	22	Give the source of funding and the role of the funders for the present study and, if applicable, for the original study on which the present article is based	10

*Give information separately for exposed and unexposed groups.

Note: An Explanation and Elaboration article discusses each checklist item and gives methodological background and published examples of transparent reporting. The STROBE checklist is best used in conjunction with this article (freely available on the Web sites of PLoS Medicine at http://www.plosmedicine.org/, Annals of Internal Medicine at http://www.annals.org/, and Epidemiology at http://www.epidem.com/). Information on the STROBE Initiative is available at http://www.strobe-statement.org.

For peer review only

BMJ Open

The Nonlinear Association between Age and Reported Opioid Use and the Role of Socioeconomic Status: Cohort Study Using Health Survey for England Waves from 1997 to 2014

Journal:	*BMJ Open*
Manuscript ID	bmjopen-2021-057428.R1
Article Type:	Original research
Date Submitted by the Author:	14-Sep-2022
Complete List of Authors:	Nowakowska, Magdalena; The University of Manchester, Centre for Primary Care and Health Services Research<br>Zghebi, Salwa; The University of Manchester, Centre for Primary Care and Health Services Research<br>Chen, Li-chia; The University of Manchester, Division of Population Health, Health Services Research and Primary Care; School of Health Sciences; Faculty of Biology, Medicine and Health<br>Kontopantelis, Evangelos; The University of Manchester, Division of Informatics, Imagine and Data Sciences
**Primary Subject Heading**:	Epidemiology
Secondary Subject Heading:	Health services research, Epidemiology, Public health
Keywords:	EPIDEMIOLOGY, PAIN MANAGEMENT, SOCIAL MEDICINE

SCHOLARONE™
Manuscripts

I, the Submitting Author has the right to grant and does grant on behalf of all authors of the Work (as defined in the below author licence), an exclusive licence and/or a non-exclusive licence for contributions from authors who are: i) UK Crown employees; ii) where BMJ has agreed a CC-BY licence shall apply, and/or iii) in accordance with the terms applicable for US Federal Government officers or employees acting as part of their official duties; on a worldwide, perpetual, irrevocable, royalty-free basis to BMJ Publishing Group Ltd ("BMJ") its licensees and where the relevant Journal is co-owned by BMJ to the co-owners of the Journal, to publish the Work in this journal and any other BMJ products and to exploit all rights, as set out in our licence.

The Submitting Author accepts and understands that any supply made under these terms is made by BMJ to the Submitting Author unless you are acting as an employee on behalf of your employer or a postgraduate student of an affiliated institution which is paying any applicable article publishing charge ("APC") for Open Access articles. Where the Submitting Author wishes to make the Work available on an Open Access basis (and intends to pay the relevant APC), the terms of reuse of such Open Access shall be governed by a Creative Commons licence – details of these licences and which Creative Commons licence will apply to this Work are set out in our licence referred to above.

Other than as permitted in any relevant BMJ Author's Self Archiving Policies, I confirm this Work has not been accepted for publication elsewhere, is not being considered for publication elsewhere and does not duplicate material already published. I confirm all authors consent to publication of this Work and authorise the granting of this licence.

For peer review only

The Nonlinear Association between Age and Reported Opioid Use and the Role of Socioeconomic Status: Cohort Study Using Health Survey for England Waves from 1997 to 2014

Magdalena Nowakowska,[1,2,3] Salwa S Zghebi,[1,4] Li-Chia Chen,[5] Evangelos Kontopantelis,[1,2]

1. NIHR School for Primary Care Research; Centre for Primary Care and Health Services Research; Manchester Academic Health Science Centre (MAHSC), University of Manchester, M13 9PL, Manchester, UK
2. Division of Data Informatics, Imaging and Data Sciences; School of Health Sciences; Faculty of Biology, Medicine and Health; Manchester Academic Health Science Centre (MAHSC), University of Manchester, M13 9PL, Manchester, UK
3. NIHR Greater Manchester Patient Safety Translational Research Centre; University of Manchester, M13 9PL, Manchester, UK
4. Division of Population Health, Health Services Research and Primary Care; School of Health Sciences; Faculty of Biology, Medicine and Health; Manchester Academic Health Science Centre (MAHSC), University of Manchester, M13 9PL, Manchester, UK
5. Division of Pharmacy and Optometry; School of Health Sciences; Faculty of Biology, Medicine and Health; Manchester Academic Health Science Centre (MAHSC), University of Manchester, M13 9PL, Manchester, UK

Corresponding Author
Evangelos Kontopantelis
Suite 5, 5th floor, Williamson Building,
176 Oxford Road, M13 9PL Manchester
e.kontonpantelis@manchester.ac.uk

Word count: 2,834
Number of tables: 2
Number of figures: 3

For peer review only - http://bmjopen.bmj.com/site/about/guidelines.xhtml

ABSTRACT

Background: Age and socioeconomic status (SES) predict several health-related outcomes, including prescription opioid use. Contrasting findings from previous literature found higher prevalence of opioid use in both people over 65 years old and the working-age population of 35-55 years old. This study aimed to analyse if the association between age and opioid use is nonlinear and differs in adults with different SES levels.

Methods: This cohort study utilised the Health Survey for England waves 1997 to 2014 data to investigate the shape of the correlation between reported opioid use and income decile, employment status, and educational level. A semi-parametric generalised additive model (GAM) was employed, so that linearity of correlation was not assumed. The shape of the relationship was assessed using the effective degrees of freedom (EDF).

Results: Positive correlation between age and reported opioid use, more linear in people in the highest income decile (EDF: 1.01M, p<0.001) and higher education (EDF: 2.03, p<0.001) was observed. In people on lower income and with lower levels of education, the probability of reported opioid use peaks was at around 40 to 60 years old and slowly decreased after that. Higher income decile and higher levels of education were predictors of a lower probability of reported opioid use (OR: 0.27, 95%CI: 0.21 to 0.36 and OR: 0.48, 95%CI: 0.41 to 0.57 respectively). There was no statistically significant difference in opioid use between employed and unemployed people.

Conclusion: The relationship between age and the probability of prescribed opioid use varies greatly across different income and educations strata, highlighting different drivers in opioid prescribing across population groups. More research is needed into exploring patterns in opioid use in older people, particularly from disadvantaged socioeconomic backgrounds.

For peer review only

For peer review only - http://bmjopen.bmj.com/site/about/guidelines.xhtml

Strengths and Limitations of this Study:

- The use of Generalised Additive Models to investigate the relationship between age and reported opioid use within different socioeconomic strata allowed exploration of the correlation without the assumption of linearity
- Due to limitations in recorded data, the distinction between type, strengths and duration of opioids was not accounted for
- Survey data used in this study is sensitive to self-reporting bias
- The low prevalence of opioid users limited the number of covariates used in the statistical model

INTRODUCTION

Opioid painkillers are effective analgesics that can provide crucial pain relief for patients with acute or cancer pain, which are often still used for the treatment of chronic non-cancer pain, although evidence for the latter indicate higher risk of short-term harms and limited or no benefits compared to nonopioid therapy.[1] Extensive research suggests a dose and potency dependent association between prescribed opioid use and several adverse severe outcomes in patients who persistently use them.[2,3] Prescribed opioid use, as well as the associated adverse effects, varies across individuals and communities.[4,5] A growing body of academic literature focuses on the biological, psychosocial, and institutional factors that influence the likelihood of prescribed opioid use. Socioeconomic status (SES), which can be defined through several measures, including income, occupational status, and education, was found to be closely associated with opioid prescribing[5] as well as female gender,[6] ethnicities,[7] and presence of psychological comorbidities.[8] Age is one of the strongest predictors of health and health outcomes, and the factor most commonly included in predictive models. However, the association between age and prescribed opioid use is not necessarily linear.

Older people are more likely to experience complex health problems and often in need of multiple prescribed medications.[9] Several studies found that older people are more likely to use opioid analgesia.[10] This could be partially explained by the opioids' recommended indications in palliative care and the higher prevalence of painful conditions in older people.[11] In contrast, some studies observed a higher prevalence of prescribed opioid use in 35-49 year olds.[12] Working-age adults may be more likely to be exposed to trauma and experience occupation-related pain. In a UK-based study of prescribed opioid use, the highest proportion of new users of weak and moderate opioids were aged 35 to 54 years old, whereas over 50% of new users of strong opioids were 75 years and older.[13]

The relationship between age and opioid analgesia need is complex and influenced by many factors, including race, underlying mental health conditions and socioeconomic circumstances. A nearly 300% difference in opioid prescription prevalence was observed across the race/ethnicity-income gradient in the US.[14] In Sweden, the highest risk of prescribed opioid use was observed in medium-income women aged 65 years or older, living alone and with psychological distress; whereas the lowest risk was observed in low-income men aged 18-44, living alone and without psychological distress.[15] Socioeconomic status, including income, employment status and education level, can determine persons living and working conditions, access and interactions with the healthcare system, and overall health status, changing personal health and opioid-related life trajectories. This study hypothesises that the association between age and reported prescribed opioid use varies by SES level. We used generalised additive models to examine this hypothesis, a regression analysis method that allows exploration of the non-linearity of the relationship between age and prescribed opioid use within groups of different income, employment status, and education levels. Different associations between

For peer review only - http://bmjopen.bmj.com/site/about/guidelines.xhtml

age and opioid prescribing, across socio-economic strata, would potentially highlight the presence of different health needs and drivers for prescribing. This would have implications for research, since we would need to understand the underlying cause or causes of this variation, to inform policy and improve patient care.

METHODS

Data Source

This cohort study used the Health Survey for England (HSE), a national annual survey designed to monitor the nation's health and care trends.[16] Participants were offered a nurse visit during which several questions about medication use and measurements such as height and weight are recorded. Data on opioid use was available for extraction from surveys conducted between 1997 and 2014, and therefore, these data were analysed in this study. The HSE surveys are reviewed yearly by the Research Ethics Committee (East Midlands - Nottingham 2), and informed consent was obtained from all participants. All data used in this analysis is freely available and no further approvals are required.[17]

Study Population

Participants in the HSE are randomly chosen, for each wave, from all private households' addresses in England. Participants aged 18 and older with no reported diagnosis of cancer who completed the nurse interview part of the survey were included in the study. At a number of annual waves (1997-2014), a boost sample of a population with specific characteristics was collected. To minimise bias, we excluded the boost sample of people living in care homes which were included in the 2000- and 2005-year waves. The ethnic minority boost sample included at waves 1999 and 2004 was included in the analysis.

Definition of Opioid Use

Participants were asked if they take any prescribed medications and, if yes, permission was sought for a nurse to see its container and to record the name of the medication. Up to 22 prescribed medications were recorded using the British National Formulary (BNF) Codes. Medications coded as opioid analgesics (BNF code 40702) were included in this study. If at least on opioid analgesic was recorded, participant was coded as opioid users, and otherwise, they were coded as not an opioid users.

Socioeconomic Status Measures

Socioeconomic status was measured using three commonly used indicators: income, employment status, and education level. Income was measured using equalised income variable deciles from lowest to highest. This measure considers different financial resource requirements of different households by adjusting total household income for the number of adults and children in the household. Employment was categorised using participants' reported activity in the week before the interview. Education was categorised using participants' reported highest qualification achieved at the time of the interview. People with missing employment or education data were excluded from the main analysis due to small numbers (n=45; 0.04% and n=105; 0.08%, respectively). A larger number of people had missing income information (n=19,007; 15.2%); therefore, a separate category of "missing income" was included to investigate if they represent a different population.

Statistical Analysis

Descriptive statistics for people with and without reported opioid use were calculated. To analyse the effect of age on the likelihood of reported opioid use in groups of different socioeconomic status, a semi-parametric generalised additive model (GAM) was implemented in the following form:

For peer review only - http://bmjopen.bmj.com/site/about/guidelines.xhtml

$$\log\left(\frac{P(Y=1 \mid X=x)}{P(Y=0 \mid X=x)}\right) = \alpha + \beta X + f(age)(z_i)$$

In the parametric portion of the model, α is the intercept and β is the vector of parameters associated with a set of explanatory variables X. The results are presented in odds ratios (ORs) and the corresponding 95% confidence intervals (95%CI).

The non-parametric portion of the model is formed by the smoothing function f for age which can vary for each category of SES measure zi. GAM is an extension of the commonly used Generalised Linear Model (GLM), which relaxes the hypothesis that the relationship between predictor and outcome variable is linear.[18] Instead, the relationship is modelled using smooth functions which can take any shape. Varying the shape of the smooth function by category of SES allows better interpretation of the relationship between age and opioid use in groups with different income, employment status, and education levels. This relationship can be interpreted using the effective degrees of freedom (EDF) which can indicate whether the relationship is linear (EDF=1) or non-linear (EDF>1). Furthermore, graphical visualisation of the relationship is provided to aid the interpretation. Models were controlled for available demographics: sex and ethnicity.

As a sensitivity analysis, all analyses were performed separately for females and males to investigate if different patterns emerge. These analyses showed no noticeable difference and are presented in the Supplementary Material (Tables SM1 and SM2, figures SM1-SM6). All analyses were performed using R version 3.6.3, and GAM estimations were performed using the *mgcv* package.[19]

Patient and Public Involvement

No patients were involved in the design, implementation or writing of this paper. The results will be disseminated to the appropriate audience.

RESULTS

In total, 124,740 people met the inclusion criteria, with 2,470 (1.98%) reporting opioid use across all waves (Table 1). The mean age of opioid users was higher than non-users (59.00 ± 15.38 vs. 48.47 ± 17.75). A higher proportion of females and white people were opioid users than males and people of any other ethnicity (2.23% vs 1.67% and 2.13% vs. 0.86%, respectively). After adjusting for all covariates, in all models with the three SES indicators, females, white people, and people living in urban areas were more likely to report opioid use (Table 2).

For peer review only

Table 1: Characteristics of all eligible participants from HSE 1997-2014

	Opioid non-users	Opioid users
Number (%)	122,270 (98.02)	2,470 (1.98)
Age, mean (± SD)	48.47 (±17.75)	59.00 (±15.38)
Gender		
Female, N (%)	67,584 (97.77)	1,544 (2.23)
Male, N (%)	54,686 (98.33)	926 (1.67)
Ethnicity		
White	107,797 (97.87)	2,343 (2.13)
Non white	14,390 (99.14)	125 (0.86)
Income decile		
1 – lowest	10,300 (97.41)	274 (2.59)
2	10,288 (97.30)	286 (2.71)
3	10,218 (96.63)	356 (3.37)
4	10,272 (97.15)	301 (2.85)
5	10,342 (98.34)	231 (2.18)
6	10,398 (98.74)	175 (1.66)
7	10,440 (98.74)	133 (1.26)
8	10,475 (99.07)	98 (0.93)
9	10,461 (98.94)	112 (1.06)
10 - Highest	10,487 (99.19)	86 (0.81)
Income missing	18,589 (97.80)	418 (2.20)
Employment Status		
Student	4,123 (99.66)	14 (0.34)
Employed	69,564 (99.33)	465 (0.66)
Unemployed	2,367 (99.25)	18 (0.75)
Ill or disabled	4,726 (86.87)	714 (13.13)
Retired	29,055 (96.46)	1,067 (3.54)
Domestic worker†	11,321 (98.56)	165 (1.44)
Other	1,071 (97.72)	25 (2.28)
Employment Missing	43 (95.55)	2 (4.44)
Education*		
Higher Education	36,026 (98.79)	441 (1.21)
A-levels	13,896 (98.71)	181 (1.29)
O-levels/GCSE	31,358 (98.16)	586 (1.83)
Foreign/other	3,624 (97.84)	80 (2.16)
No qualifications	31,238 (96.51)	1,130 (3.49)
Full time student	6,026 (99.19)	49 (0.81)
Education Missing	102 (97.14)	3 (2.86)

* The O- and A-Levels examination certificates are the secondary and pre-university credentials in England, Wales and Northern Ireland. The O Levels, or Ordinary Levels, typically represent a total of 11 years of study and mark the end of the secondary education cycle. A-levels, or Advanced level qualifications, are subject-based qualifications (leading to university, further study, training, or work), studied by students in Sixth Form, which refers to the last two years of secondary education (ages 16–18). A General Certificate of Secondary Education (GCSE) is a qualification normally taken by most UK students at the end of compulsory education.

† Any person engaged in domestic work within an employment relationship

For peer review only

Compared to people in the lowest income decile, people reporting income in the highest decile were significantly less likely to report opioid use (OR: 0.27, 95%CI: 0.21 to 0.36). Compared to people who were employed the week before the interview, unemployed people were not statistically different in the likelihood of reporting opioid use. However, as expected, those classified as ill or disabled were significantly more likely to report opioid use (OR: 16.50, 95%CI: 13.24 to 20.55). People performing domestic work were also more likely to report opioid use (OR: 1.60, 95%CI: 1.32 to 1.94). Those with higher education were less likely to report opioid use than those with no qualifications (OR: 0.48, 95%CI: 0.41 to 0.57).

Table 2: Semi-parametric generalised additive models for the odds of reported prescription opioid use with smooth function for the effect of age, varying by SES measure

	**Opioid use (Model including income**	**Opioid use (Model including employment status)**	**Opioid use (Model including education)**
**Parametric coefficients**	**OR (95% CI)**	**OR (95% CI)**	**OR (95% CI)**
Intercept	0.009 (0.007 to 0.011) ***	0.003 (0.002 to 0.003) ***	0.01 (0.007 to 0.011) ***
Female (ref: Male)	1.32 (1.21 to 1.43) ***	1.53 (1.40 to 1.67) ***	1.33 (1.22 to 1.44) ***
White (ref: Not white)	2.24 (1.86 to 2.70) ***	2.01 (1.67 to 2.42)***	1.94 (1.61 to 2.32) ***
Urban (ref: Rural)	1.27 (1.15 to 1.41) ***	1.20 (1.08 to 1.33) ***	1.29 (1.17 to 1.43) ***
Income Decile (ref: 1-lowest)			
2	0.93 (0.76 to 1.13)		
3	1.01 (0.82 to 1.24)		
4	0.80 (0.65 to 0.99) *		
5	0.55 (0.43 to 0.70) ***		
6	0.51 (0.40 to 0.64) ***		
7	0.38 (0.29 to 0.49) ***		
8	0.34 (0.26 to 0.44) ***		
9	0.40 (0.32 to 0.51) ***		
10 – Highest	0.27 (0.21 to 0.36) ***		
Income Missing	0.64 (0.53 to 0.77) ***		
Economic status (ref: Employed)			
Student		0.92 (0.36 to 2.35)	
Unemployed		1.61 (0.97 to 2.64)	
Ill or disabled		16.50 (13.24 to 20.55) ***	
Retired		1.60 (0.93 to 2.76)	
Domestic worker		1.60 (1.32 to 1.94) ***	
Other		3.14 (2.02 to 4.87) ***	
Education (ref: No qualifications)			
Higher Education			0.48 (0.41 to 0.57) ***
A-levels			0.57 (0.47 to 0.70) ***
O-levels/GCSE			0.71 (0.61 to 0.83) ***
Foreign/other			0.63 (0.43 to 0.91) *
Student			0.57 (0.44 to 0.82) **
**Non Parametric**	**EDF**	**EDF**	**EDF**
Income Decile:			
1 – Lowest	3.97 ***		
2	6.08 ***		
3	4.17 ***		
4	3.31 ***		

For peer review only - http://bmjopen.bmj.com/site/about/guidelines.xhtml

5	2.64 ***		
6	2.51 ***		
7	2.06 ***		
8	1.52 ***		
9	1.04 ***		
10 – Highest	1.01 ***		
Income Missing	3.30 ***		
Economic status:			
Employed		1.01 ***	
Student		1.002	
Unemployed		1.002 *	
Ill or disabled		3.12 ***	
Retired		4.99 **	
Domestic worker		2.74 ***	
Other		1.76 *	
Education:			
Higher Education			2.03 ***
A-levels			2.93 ***
O-levels/GCSE			3.30 ***
No qualifications			4.30 ***
Foreign/Other			1.01 **
Student			2.81 ***

OR – Odds Ratio; CI: Confidence Intervals; EDF - Estimated Degrees of Freedom
** p-value < 0.05*
*** p-value < 0.01*
**** p-value <0.001*

The effect of age on the likelihood of reporting opioid use was non-linear and significant in all income deciles as suggested by the statistically significant EDF. The higher the income decile, the relationship between age and probability of opioid use approached linear relationship with the EDF: 1.01, $p<0.001$ for highest income decile and EDF: 3.97, $p<0.001$ for lowest income decile. This suggests that in people with higher income, older people were more likely to report opioid use. However, in lower-income deciles, the relationship was non-linear. In particular, in people on the lowest income decile, the probability of opioid use increased with age until approximately 55 years old, after which it reduced (Figure 1).

Concerning employment status, age had a statistically significant association with reported opioid use in people who were employed (EDF: 1.01, $p<0.001$), ill or disabled (EDF: 3.12, $p<0.001$), domestic workers (EDF: 2.74, $p<0.001$), and with lower significance levels, retired (EDF: 4.99, $p<0.01$), unemployed (EDF: 1.002, $p<0.05$), and other economic statuses (EDF: 1.76, $p<0.05$). In people who were employed and unemployed, the relationship approached linearity (EDF: 1.01, $p<0.001$ and EDF: 1.002, $p<0.05$ respectively), whereas in people who were ill or disabled, the distribution was slightly skewed towards people between 40 and 60 years old, and in domestic workers, the likelihood peaked at around 60 years of age (Figure 2).

For all levels of education status, the effect of age on the odds of reporting opioid use was statistically significant. The effect was almost linear for people with foreign/other qualifications (Figure 3). In people with higher education, older age was associated with higher odds of reported opioid use. In contrast, in people with lower qualifications (A-levels, O-levels/GCSE) the odds of reported opioid use

For peer review only - http://bmjopen.bmj.com/site/about/guidelines.xhtml

peaked around 60 years old slightly decrease after that. The odds of reported opioid use peaked earlier in people with no qualifications at around 50 years old.

DISCUSSION

This study found that the relationship between age and the likelihood of reported opioid use changes with different measures of socioeconomic status. In people with higher income and education, the relationship is more linear, increasing with age. In people on lower income with lower levels of education, the likelihood of reported opioid use peaks at around 40 to 60 years old and slowly decreases after that. Consistently with previous research, females, white people, those living in urban areas, and those with lower levels of SES were more likely to report opioid use. The difference between employed and unemployed people was not statistically significant regarding the likelihood of reported opioid use. In both groups, the association with age was linear and increasing with age.

Existing studies found mixed results regarding the correlation between age and use of opioids; some studies found a higher prevalence in older populations[10] whereas others in the working-age population.[12] This study shows that SES may contribute to the non-linear relationship between age and opioid use. Shaw, Roelofs, and Punnett (2017)[20] suggest two pathways in which working conditions, strongly correlated with income, related to opioid use. The first suggests that physical injury at work or cumulative trauma from strenuous labour leads to pain and the use of painkillers. The second relates to psychological stress from unmanageable job demands like time pressure or economic insecurity and job instability, leading to depression and anxiety, which may correlate with chronic opioid use. These adverse working conditions are expected in people with lower-paying jobs, explaining the observed higher prevalence of reported opioid use in working-age people with lower SES. Furthermore, people experiencing disability or illness who are in a greater need of opioid treatment are more likely to be on lower income due to difficulties sustaining employment, regardless of age. Interestingly, we observe a reversal in the relationship between rurality and opioid use, compared to what has been reported in the USA. [21,22] However, this is not unexpected considering the different socioeconomic make-up in the rural USA and rural UK, with rural UK areas being generally much more affluent than urban conurbations, contrary to the situation in the USA.[23]

Our findings show an increase in reported opioid use with age in people with higher income and higher education. Reported opioid use in people with lower income, and also in people with no higher education, decreased after it peaked around 40 to 60 years of age. Two possible explanations have been identified in the literature. Firstly, previous research found that the prevalence of musculoskeletal pain increases up to approximately 65 years old, after which it declines.[24] This has been linked to the decline of the adverse physical and mental effects of the workplace at the age of retirement and the lower prevalence of several diseases in surviving populations as people with chronic diseases tend to die early.[25] Furthermore, a review of pain management in the elderly showed that pain in the elderly might be untreated and misdiagnosed.[26] Conversely, a higher prevalence of opioid use would be expected in older people due to more complex health needs and the use of opioid analgesia in palliative care. Further research is needed to establish if older people from lower socioeconomic backgrounds are more likely to experience difficulties accessing adequate analgesia.

Strengths and limitations

The statistical techniques used in this study allowed modelling the relationship between age and odds of reported opioid use in a non-linear manner and present results separately for people with different

For peer review only - http://bmjopen.bmj.com/site/about/guidelines.xhtml

levels of SES. Various methods can examine the interaction between two variables, each with different strengths and limitations. A common approach includes an interaction term where the effect of a covariate of interest can vary depending on another variable.[27] Alternatively, multilevel models allow parameters of interest, including the intercept and the slope of the regression line, to vary across more than one level.[28] However, these methods assume a linear relationship. Age can be modelled as categorical, but this can lead to complex models where multiple age categories interact with multiple SES categories, and information is difficult to interpret. Other techniques, including several machine learning methods, can produce highly accurate but difficult to interpret estimations. This study employed generalised additive models, a regression analysis method that allows exploration of the non-linearity of the relationship between age and opioid use within groups of different income, employment status, and education levels. To our knowledge, this is the first study to employ this technique to explore the relationship between age and prescription opioid use in England.

However, some limitations should be acknowledged. Firstly, the relationship between age and opioid use may be specific to different opioid strengths and durations. The data records in HSE did not capture the type of prescribed opioids; hence they did not adjust for the opioid type strength in the analysis. Secondly, opioid use was assessed based on self-reported data, which may be susceptible to recall and social desirability bias. However, this risk of bias was reduced by the involvement of qualified nurses in collecting medication data. Thirdly, the low prevalence of opioid use meant that a limited number of covariates was included in the model. Some variables, such as the presence of chronic conditions, had to be excluded from the models, which could explain the different patterns of associations across socio-economic strata. Fourth, the HES, although considered representative of the English population,[16] does not include some subgroups of the population, who are not living in private households, such as the homeless (who are at higher risk of opioid abuse) and those living in communal establishments (e.g. residential and nursing homes). However, less than 2% of the population of England live in communal establishments,[29] while less than 0.5% are homeless.[30]

Conclusion

Findings from this study suggest that, although the relationship between age and odds of opioid use in people with higher income and education approaches linearity, in people with lower SES, the probability of exposure peaks between 40 to 60 years old and decreases after that. This variability in the relationship between age and the probability of prescribed opioid use, highlights different drivers in opioid prescribing across different income and educations strata. More research is needed into exploring patterns in opioid use in older people, particularly from disadvantaged socioeconomic backgrounds.

For peer review only

For peer review only - http://bmjopen.bmj.com/site/about/guidelines.xhtml

Authors contributions

All authors contributed to the design of the study. MN prepared and analysed the data. MN wrote the manuscript and MN, SSZ, LCC, and EK all critically edited the manuscript. All authors approved the manuscript before submission.

Competing interest

MN, SSZ, LCC, and EK have no conflict of interest to declare.

Role of the funding source

This study was funded as part of a PhD studentship from the National Institute for Health Research (NIHR) School for Primary Care Research (SPCR) and NIHR Greater Manchester Patient Safety Translational Research Centre. The study represents independent research by the NIHR. The views expressed in this publication are those of the authors and not necessarily those of the National Health Service, the NIHR or the Department of Health and Social Care. The study funders had no role in the study design, data collection, analysis or interpretation, in the writing of the paper or in the decision to submit the paper for publication. MN had full access to all the data in the study and had final responsibility for the decision to submit for publication.

Data sharing

All data used in this study are publicly available from the sources cited in the manuscript.

Research Ethics Approval

This study involves analysis of anonymous and publicly available data. No ethical approval was sought or required for this study.

For peer review only

Figure 1: Non-parametric estimation of the effect of age on likelihood of reported opioid use by income decile

Figure 2: Non-parametric estimation of the effect of age on likelihood of reported opioid use by economic status

Figure 3: Non-parametric estimation of the effect of age on likelihood of reported opioid use by education status

For peer review only

For peer review only - http://bmjopen.bmj.com/site/about/guidelines.xhtml

REFERENCES

1 Chou R, Hartung D, Turner J, *et al.* Opioid Treatments for Chronic Pain. *AHRQ Publ No 20-EHC011* Published Online First: 2020. doi:https://doi.org/10.23970/AHRQEPCCER229.
2 Benyamin R, Trescot AM, Datta S, *et al.* Opioid complications and side effects. 2008. doi:10.36076/ppj.2008/11/s105
3 Moore RA, McQuay HJ. Prevalence of opioid adverse events in chronic non-malignant pain: systematic review of randomised trials of oral opioids. Arthritis Res. Ther. 2005;**7**:1–6. doi:10.1186/ar1782
4 Nowakowska M, Zghebi SS, Perisi R, *et al.* Association of socioeconomic deprivation with opioid prescribing in primary care in England: A spatial analysis. *J Epidemiol Community Health* 2021;**75**:128–36. doi:10.1136/jech-2020-214676
5 Macfarlane GJ, Beasley M, Jones GT, *et al.* The epidemiology of regular opioid use and its association with mortality: Prospective cohort study of 466 486 UK biobank participants. *EClinicalMedicine* 2020;**21**:100321. doi:10.1016/j.eclinm.2020.100321
6 Serdarevic M, Striley CW, Cottler LB. Sex differences in prescription opioid use. Curr. Opin. Psychiatry. 2017;**30**:238–46. doi:10.1097/YCO.0000000000000337
7 Ringwalt C, Roberts AW, Gugelmann H, *et al.* Racial Disparities Across Provider Specialties in Opioid Prescriptions Dispensed to Medicaid Beneficiaries with Chronic Noncancer Pain. *Pain Med (United States)* 2015;**16**:633–40. doi:10.1111/pme.12555
8 Davis MA, Lin LA, Liu H, *et al.* Prescription opioid use among adults with mental health disorders in the United States. *J Am Board Fam Med* 2017;**30**:407–17. doi:10.3122/jabfm.2017.04.170112
9 Hajjar ER, Cafiero AC, Hanlon JT. Polypharmacy in elderly patients. *Am J Geriatr Pharmacother* 2007;**5**:345–51. doi:10.1016/j.amjopharm.2007.12.002
10 Bedene A, Lijfering WM, Niesters M, *et al.* Opioid Prescription Patterns and Risk Factors Associated With Opioid Use in the Netherlands. *JAMA Netw open* 2019;**2**:e1910223. doi:10.1001/jamanetworkopen.2019.10223
11 Gibson SJ, Lussier D. Prevalence and Relevance of Pain in Older Persons. Pain Med. 2012;**13**:S23–6. doi:10.1111/j.1526-4637.2012.01349.x
12 Rasu RS, Knell ME. Determinants of opioid prescribing for nonmalignant chronic pain in US outpatient settings. *Pain Med (United States)* 2018;**19**:524–32. doi:10.1093/pm/pnx025
13 Jani M, Yimer BB, Sheppard T, *et al.* Time trends and prescribing patterns of opioid drugs in UK primary care patients with non-cancer pain: A retrospective cohort study. *PLoS Med* 2020;**17**:e1003270. doi:10.1371/journal.pmed.1003270
14 Friedman J, Kim D, Schneberk T, *et al.* Assessment of Racial/Ethnic and Income Disparities in the Prescription of Opioids and Other Controlled Medications in California. *JAMA Intern Med* 2019;**179**:469–76. doi:10.1001/jamainternmed.2018.6721
15 Persmark A, Wemrell M, Zettermark S, *et al.* Precision public health: Mapping socioeconomic disparities in opioid dispensations at Swedish pharmacies by Multilevel Analysis of Individual Heterogeneity and Discriminatory Accuracy (MAIHDA). *PLoS One* 2019;**14**:e0224008. doi:10.1371/journal.pone.0220322
16 Mindell J, Biddulph JP, Hirani V, *et al.* Cohort profile: The health survey for england. *Int J Epidemiol* 2012;**41**:1585–93. doi:10.1093/ije/dyr199
17 NHS Digital. Health Survey for England - NHS Digital.
18 Hastie T, Tibshirani R. Generalized additive models for medical research.
19 Wood Simon. Mixed GAM Computation Vehicle with Automatic Smoothness Estimation. 2021. doi:10.1201/9781315370279
20 Shaw WS, Roelofs C, Punnett L. Work environment factors and prevention of opioid-related deaths. *Am J Public Health* 2020;**110**:1235–41. doi:10.2105/AJPH.2020.305716
21 Palombi LC, St Hill CA, Lipsky MS, *et al.* A scoping review of opioid misuse in the rural United

For peer review only - http://bmjopen.bmj.com/site/about/guidelines.xhtml

States. *Ann Epidemiol* 2018;**28**:641–52.
22 Rigg KK, Monnat SM. Urban vs. rural differences in prescription opioid misuse among adults in the United States: Informing region specific drug policies and interventions. *Int J Drug Policy* 2015;**26**:484–91.
23 Rae A. Spatially Concentrated Deprivation in England: An Empirical Assessment. *Reg Stud* 2012;**46**:1183–99. doi:10.1080/00343404.2011.565321
24 Parsons S, Breen A, Foster NE, *et al.* Prevalence and comparative troublesomeness by age of musculoskeletal pain in different body locations. *Fam Pract* 2007;**24**:308–16. doi:10.1093/fampra/cmm027
25 Cimmino MA, Ferrone C, Cutolo M. Epidemiology of chronic musculoskeletal pain. 2011.
26 Noroozian M, Raeesi S, Hashemi R, *et al.* Pain: The neglect issue in old people's life. *Open Access Maced J Med Sci* 2018;**6**:1773–8. doi:10.3889/oamjms.2018.335
27 Kontopantelis E, Sperrin M, Mamas MA, *et al.* Investigating heterogeneity of effects and associations using interaction terms. J. Clin. Epidemiol. 2018;**93**:79–83. doi:10.1016/j.jclinepi.2017.09.012
28 Peugh JL. A practical guide to multilevel modeling. *J Sch Psychol* 2010;**48**:85–112. doi:10.1016/j.jsp.2009.09.002
29 Office of National Statistics. 2011 Census analysis: What Does the 2011 Census Tell Us About People Living in Communal Establishments. 2015.
30 Shelter. 274,000 people in England are homeless, with thousands more likely to lose their homes. 2021.

For peer review only

For peer review only - http://bmjopen.bmj.com/site/about/guidelines.xhtml

Figure 1: Non-parametric estimation of the effect of age on likelihood of reported opioid use by income decile

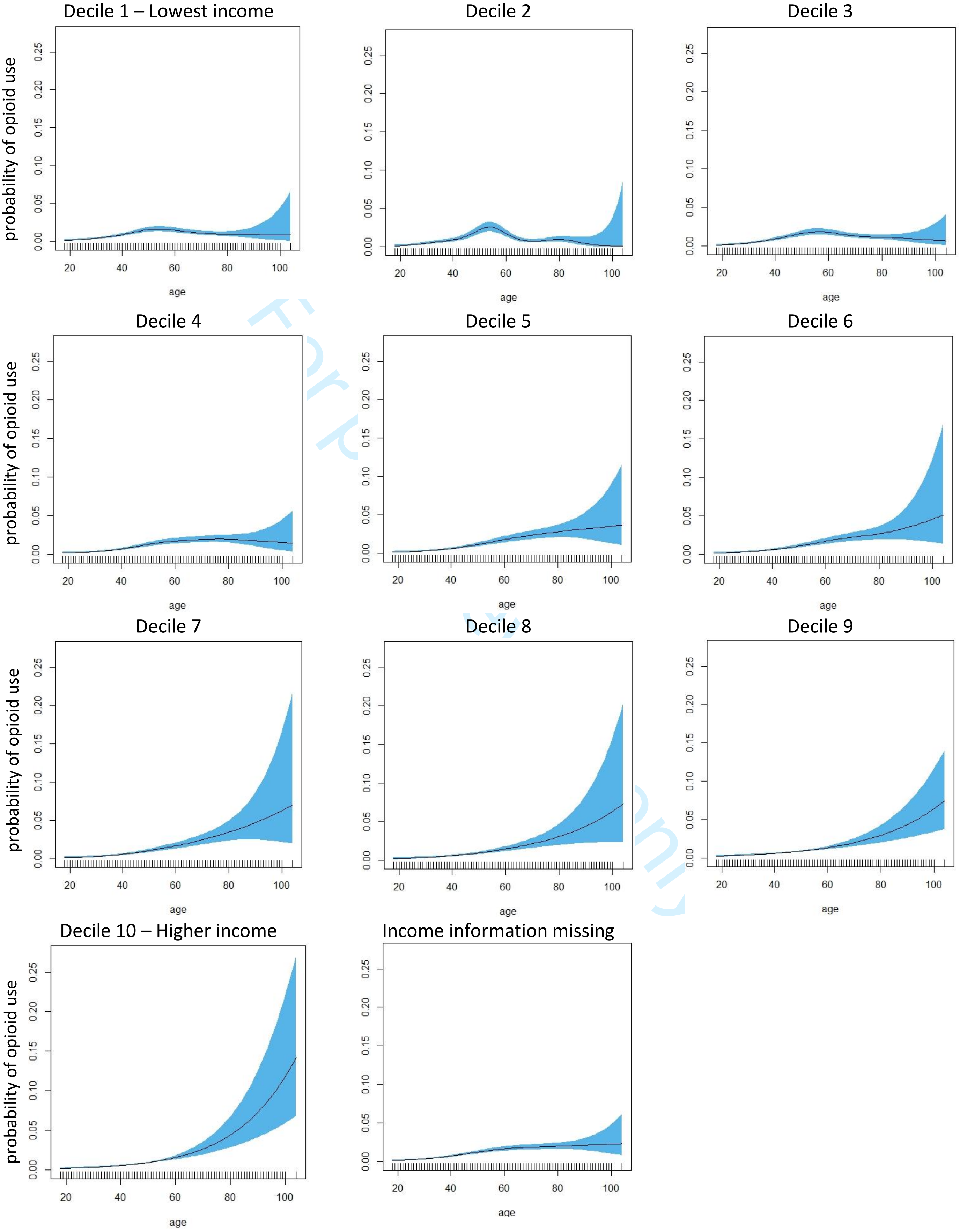

Figure 2: Non-parametric estimation of the effect of age on likelihood of reported opioid use by economic status

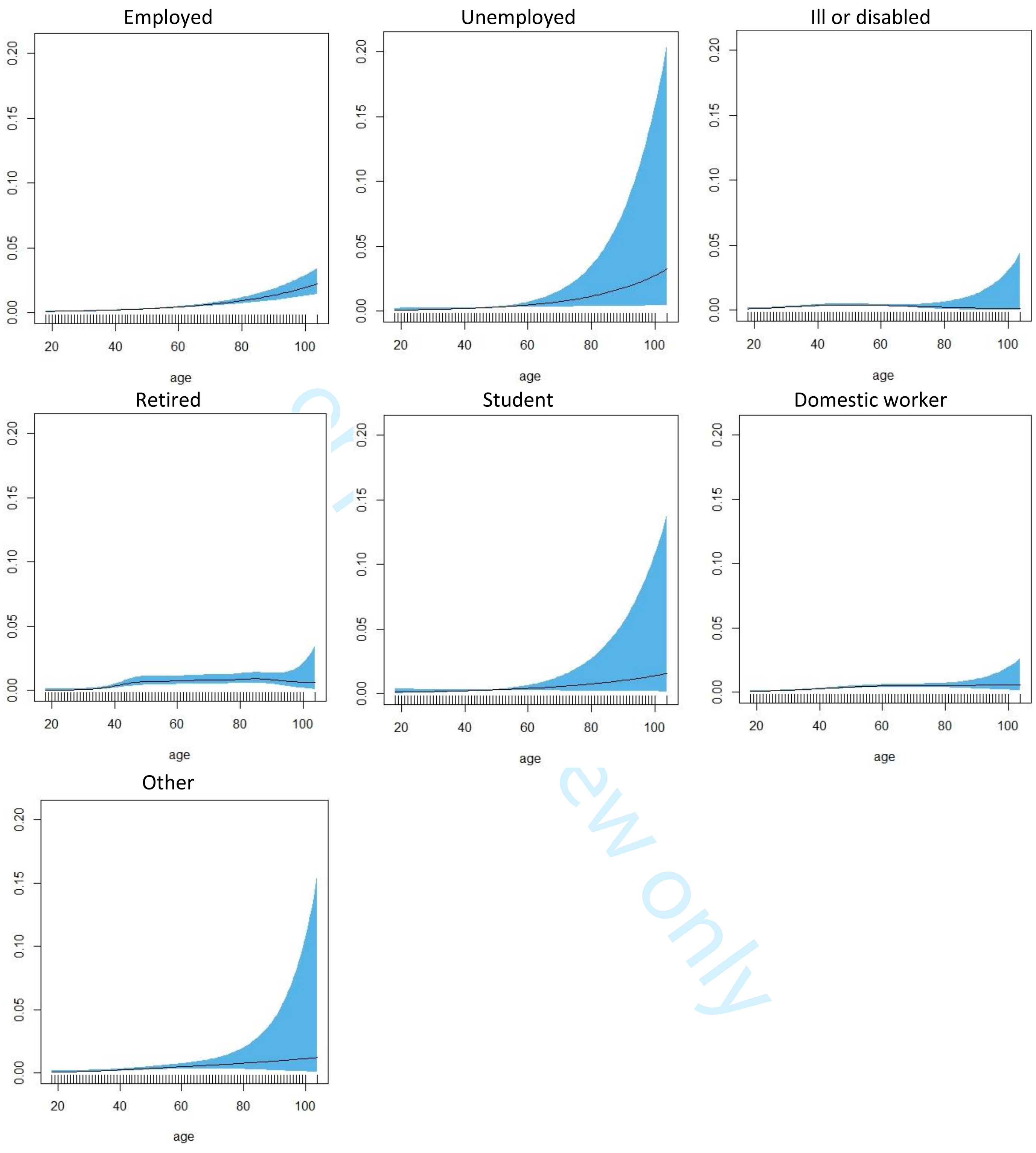

Figure 3: Non-parametric estimation of the effect of age on likelihood of reported opioid use by education status

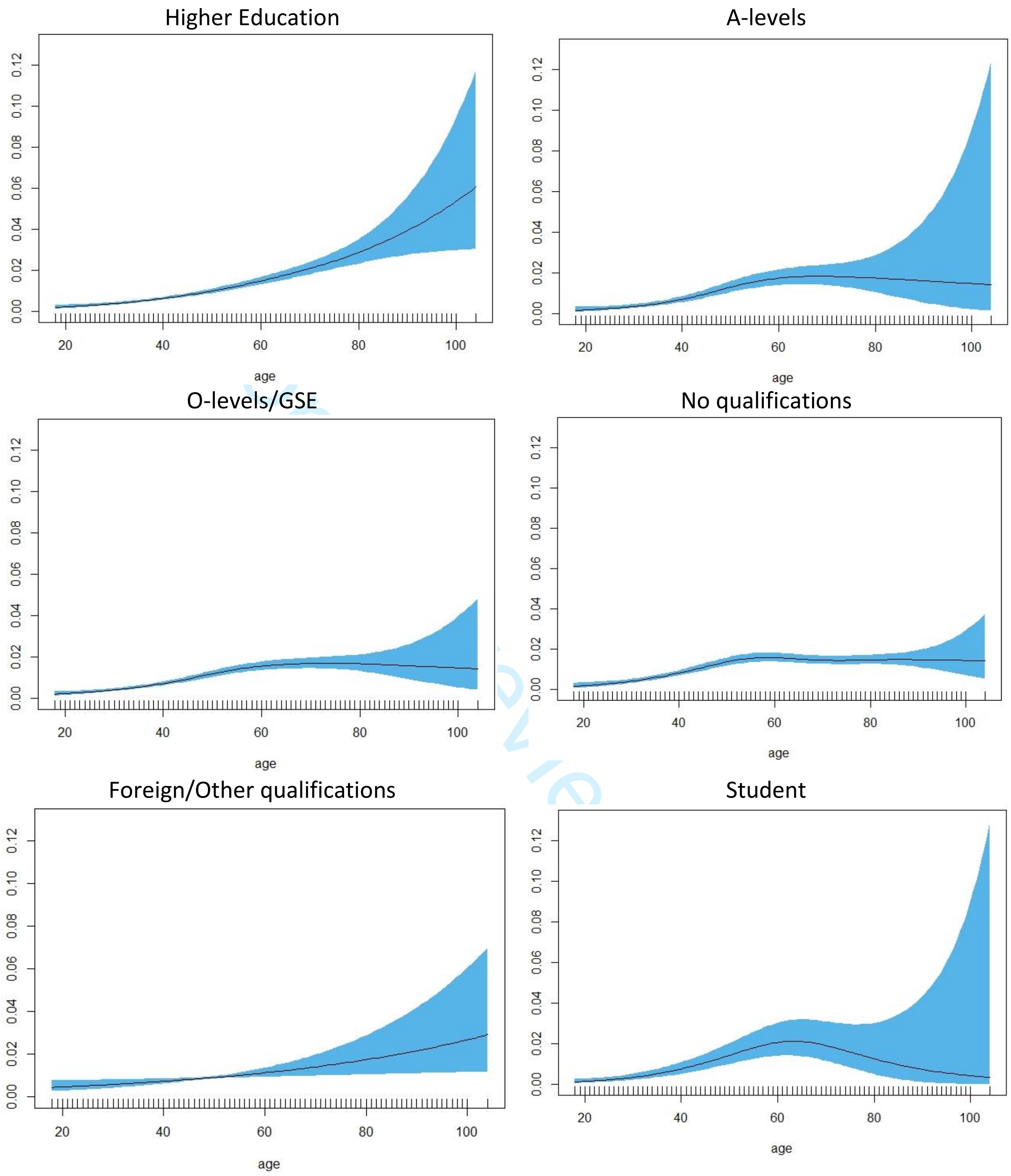

The nonlinear association between age and socioeconomic status on reported opioid use: cohort study using health survey for England waves from 1997 to 2014

Supplementary Materials: Sensitivity Analysis

For peer review only

Table SM1: Semi-parametric generalised additive models for the odds of reported prescription opioid use with smooth function for the effect of age, varying by SES measure in Females only

	Opioid use (Model including income	Opioid use (Model including employment status)	Opioid use (Model including education)
**Parametric coefficients**	**OR (95% CI)**	**OR (95% CI)**	**OR (95% CI)**
Intercept	0.01 (0.01:0.01) ***	0.004 (0.003:0.006) ***	0.012 (0.009:0.159) ***
White (ref: Not white)	2.11 (1.66:2.67) ***	1.91 (1.51:2.43)***	1.90 (1.50:2.41)***
Urban (ref: Rural)	1.30 (1.14:1.48) ***	1.23 (1.08:1.40)**	1.31 (1.14:1.49)***
Income Quantile (ref: 1-Lowest)			
Income 2	0.98 (0.81:1.19)		
Income 3	0.71 (0.57:0.87) ***		
Income 4	0.47 (0.38:0.59) ***		
Income 5 - Highest	0.41 (0.32:0.52) ***		
Income Missing	0.75 (0.60:0.93) **		
Economic Status (ref: Employed)			
Domestic		1.59 (1.29:1.97) ***	
Ill or disabled		17.52 ***(12.97:23.66)	
Other		3.04 (1.70:5.43)***	
Retired		1.75 (1.70:5.43)*	
Student		0.75 (0.19:2.80)	
Unemployed		1.78 (0.83:3.81)	
Education (ref: No qualification)			
Higher education			0.51 (0.41:0.62)***
A-levels			0.53 (0.41:0.69) ***
O-levels/GSE			0.71 (0.58:0.85)***
Foreign/Other			0.61 (0.39:0.94) *
Student			0.48 (0.31:0.75)**
**Non Parametric**	**EDF**	**EDF**	**EDF**
**Income Quantile**			
Income 1 - lowest	4.91 ***		
Income 2	3.52 ***		
Income 3	2.56 ***		
Income 4	1.12 ***		
Income 5 - highest	1.91 ***		
Income Missing	3.01 ***		

Employment status			
Domestic		2.669***	
Ill or disabled		2.626 *	
Other		1.428	
Retired		4.013 ***	
Student		1.000	
Unemployed		1.002	
Employed		1.003 ***	
Education			
Higher education			2.108***
A-levels			2.646***
O-levels/GSE			2.302***
No qualifications			3.631***
Foreign/Other			1.049*
FT Student			2.062*

For peer review only

Table SM2: Semi-parametric generalised additive models for the odds of reported prescription opioid use with smooth function for the effect of age, varying by SES measure in Males only

	**Opioid use (Model including income**	**Opioid use (Model including employment status)**	**Opioid use (Model including education)**
**Parametric coefficients**	**OR (95% CI)**	**OR (95% CI)**	**OR (95% CI)**
Intercept	0.01 (0.01:0.01) ***	0.003 (0.002:0.004)***	0.009 (0.006:0.13)***
White (ref: Not white)	2.48 (1.84:3.35) ***	2.17 (1.60:2.93)***	2.02***
Urban (ref: Rural)	1.24 (1.05:1.46) **	1.13 (0.96:1.34)	1.26**
Income Quintile (ref: 1-Lowest)	ref		
Income 2	0.85 (0.66:1.09)		
Income 3	0.34 (0.25:0.47) ***		
Income 4	0.24 (0.17:0.34) ***		
Income 5 - highest	0.30 (0.22:0.40) ***		
Income Missing	0.54 (0.41:0.71) ***		
Economic Status (ref: Employed))			
Domestic		2.82 (1.40:5.66)**	
Ill or disabled		15.51 (11.40:21.10)***	
Other		3.57 (1.89:6.74)***	
Retired		3.80 (2.66:5.43)****	
Student		0.12 (0.0002:89.32)	
Unemployed		0.33 (0.02:4.08)	
Education (ref: No qualification)			
Higher education			0.44 (0.34:0.57)***
A-levels			0.56 (0.40:0.79)***
O-levels/GSE			0.70 (0.54:0.91)**
Foreign/Other			0.65 (0.31:1.39)
Student			0.63 (0.39:1.06)
**Non Parametric**	**EDF**	**EDF**	**EDF**
**Income Quantile**			
Income 1 - lowest	4.73 ***		
Income 2	3.89 ***		
Income 3	2.69 ***		
Income 4	2.79 ***		
Income 5 - highest	1.13 ***		
Income Missing	2.89 ***		

Employment status			
Employed		1.300***	
Domestic		1.001*	
Ill or disabled		2.900**	
Other		1.002*	
Retired		1.002	
Student		2.742	
Unemployed		3.232	
Education			
Higher education			1.002***
A-levels			2.878***
O-levels/GSE			3.929***
No qualifications			3.695***
Foreign/Other			1.002
Student			2.741***

Figure SM1: Non-parametric estimation of the effect of age on likelihood of reported opioid use by income quantile in females

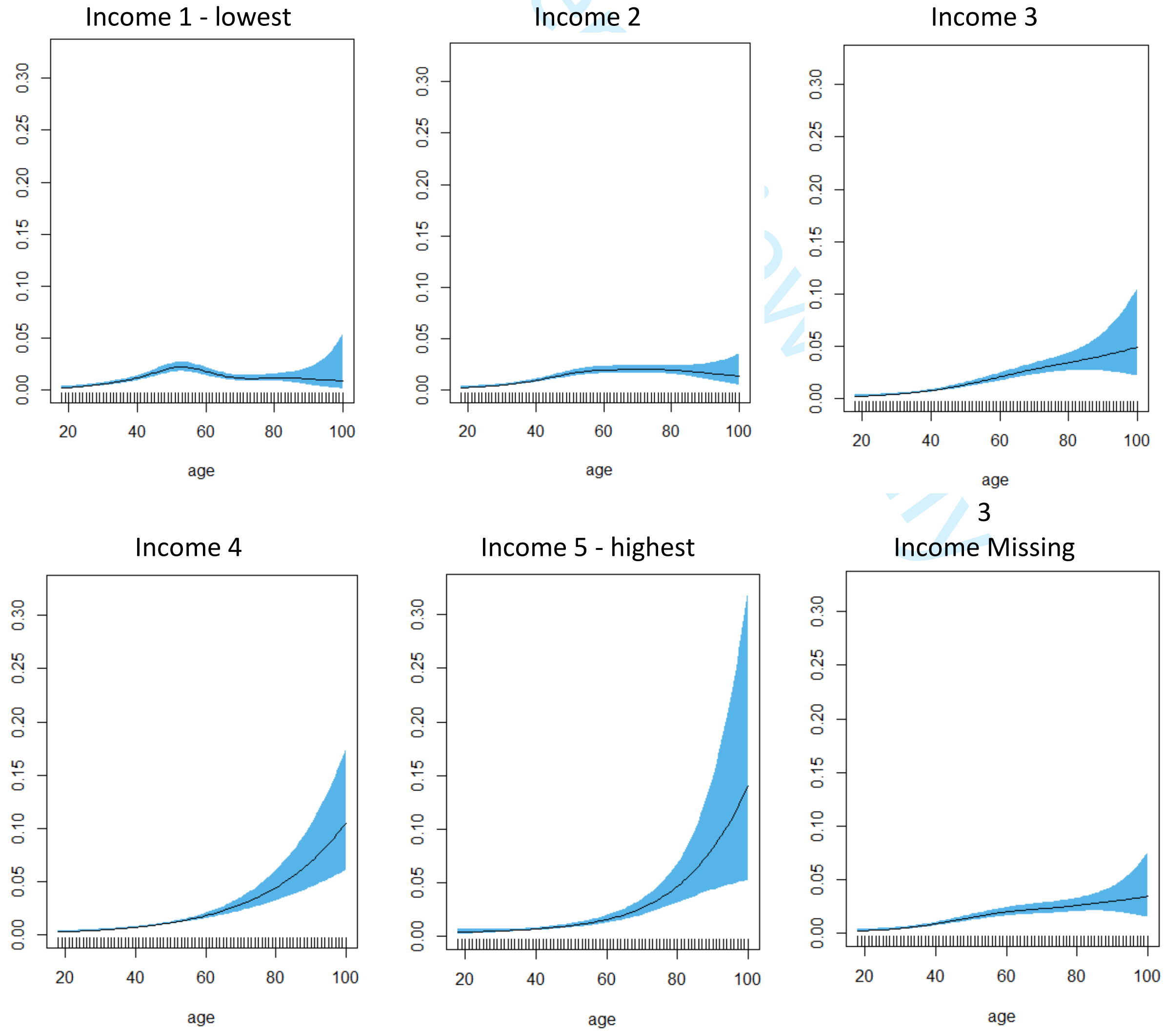

For peer review only

Figure SM2: Non-parametric estimation of the effect of age on likelihood of reported opioid use by employment status in females

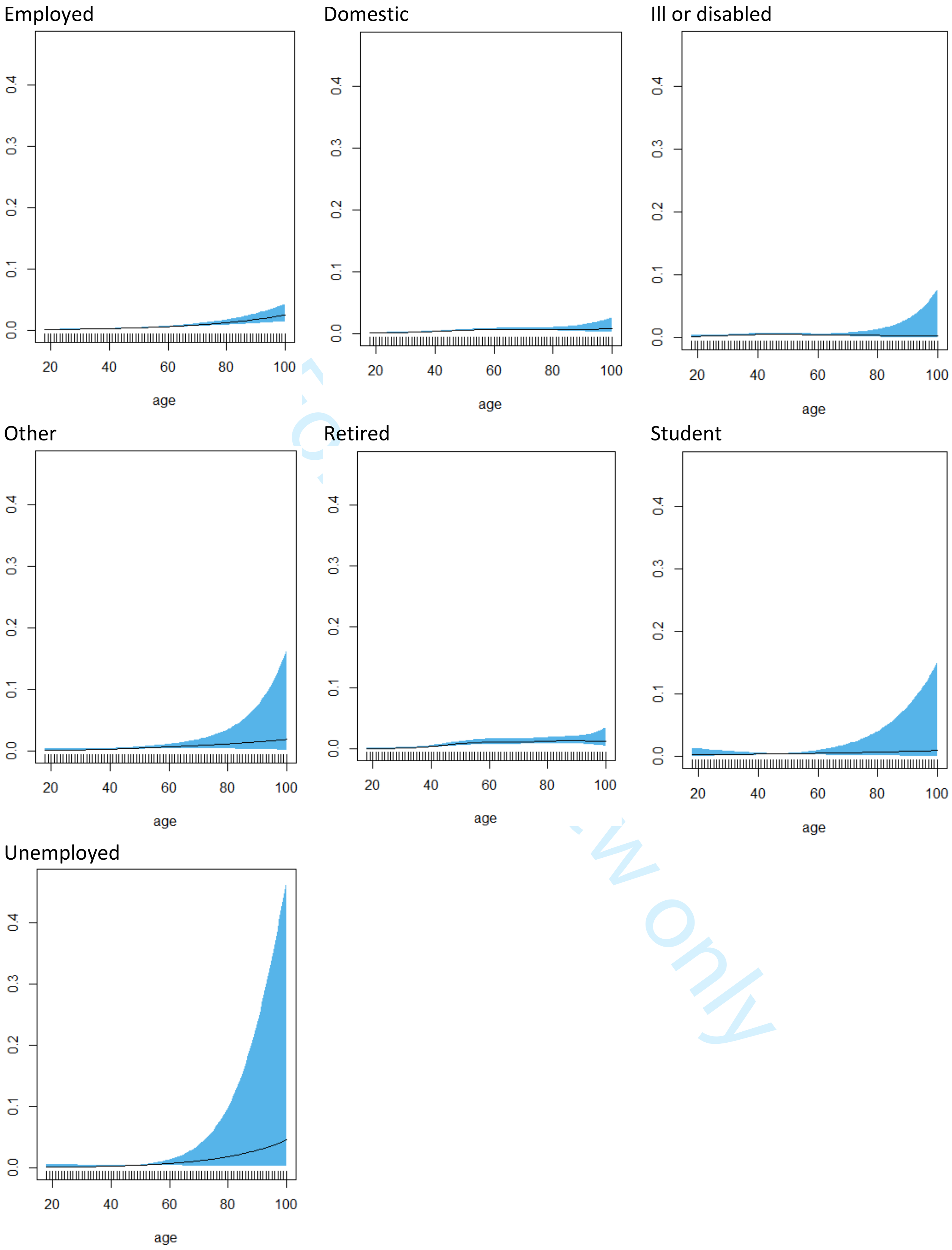

Figure SM3: Non-parametric estimation of the effect of age on likelihood of reported opioid use by income highest qualification in females

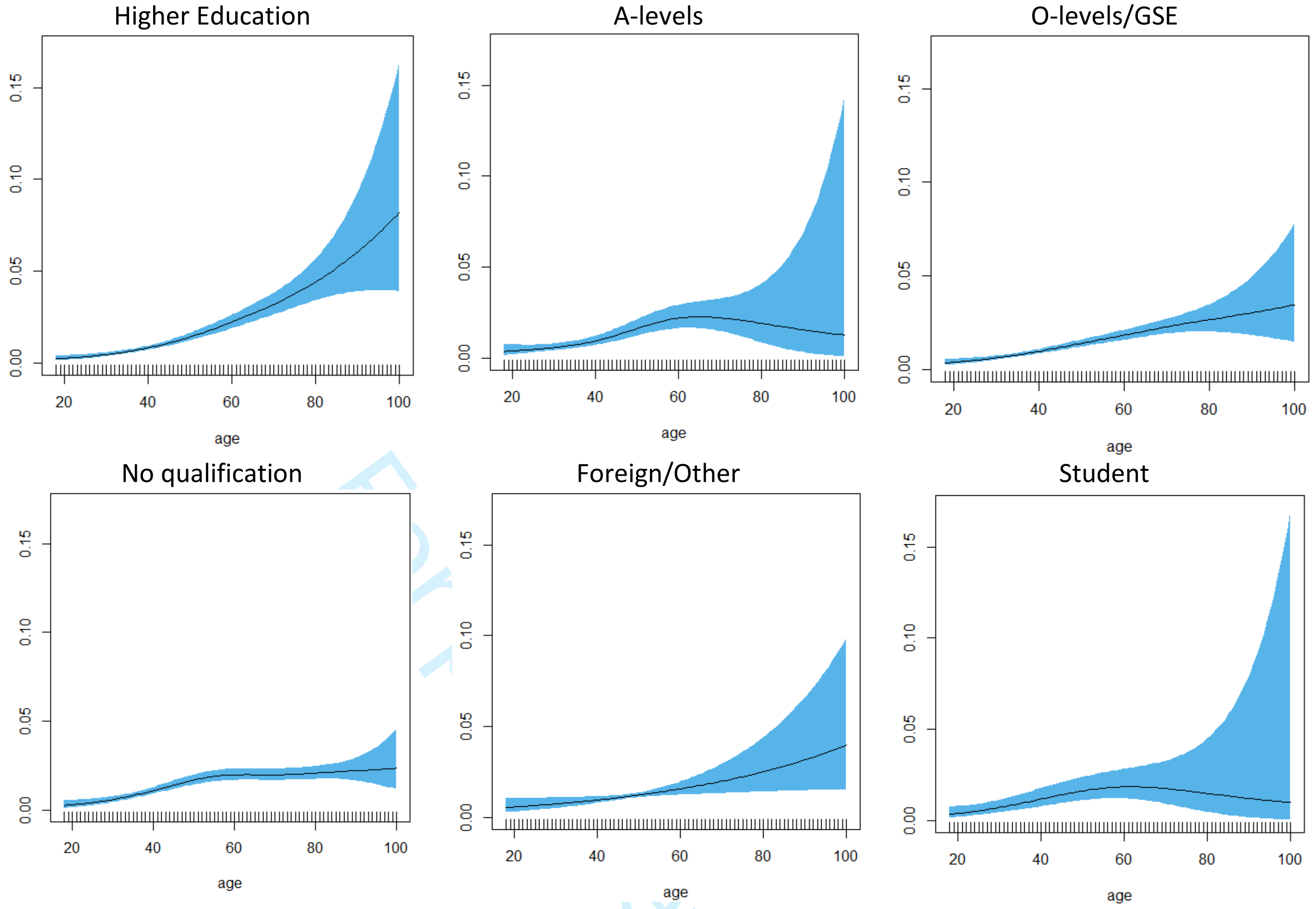

Figure SM4: Non-parametric estimation of the effect of age on likelihood of reported opioid use by income quantile in males

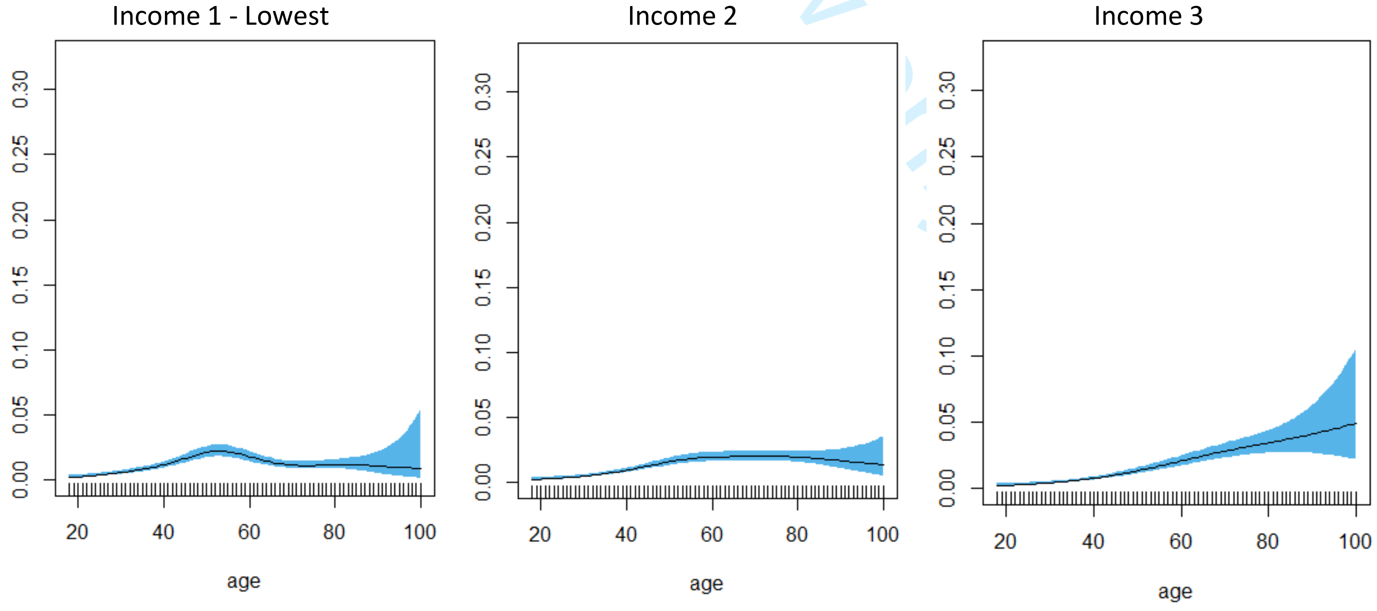

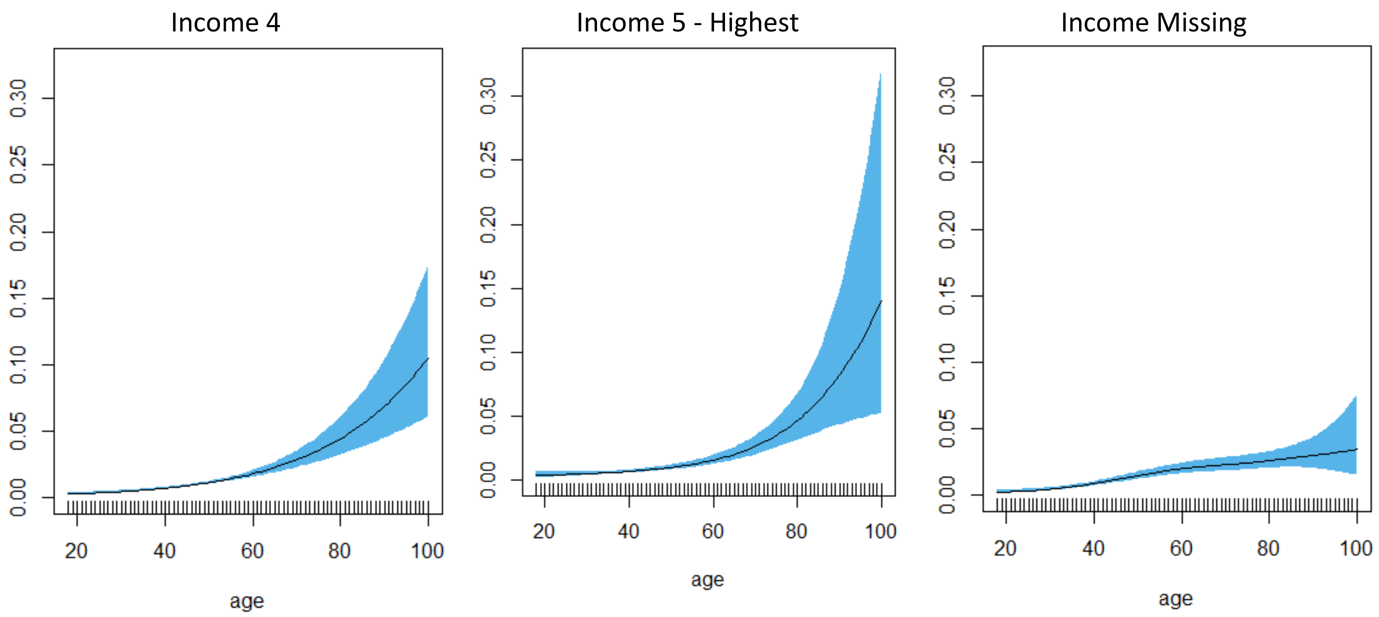

For peer review only

Figure SM5: Non-parametric estimation of the effect of age on likelihood of reported opioid use by employment status in males

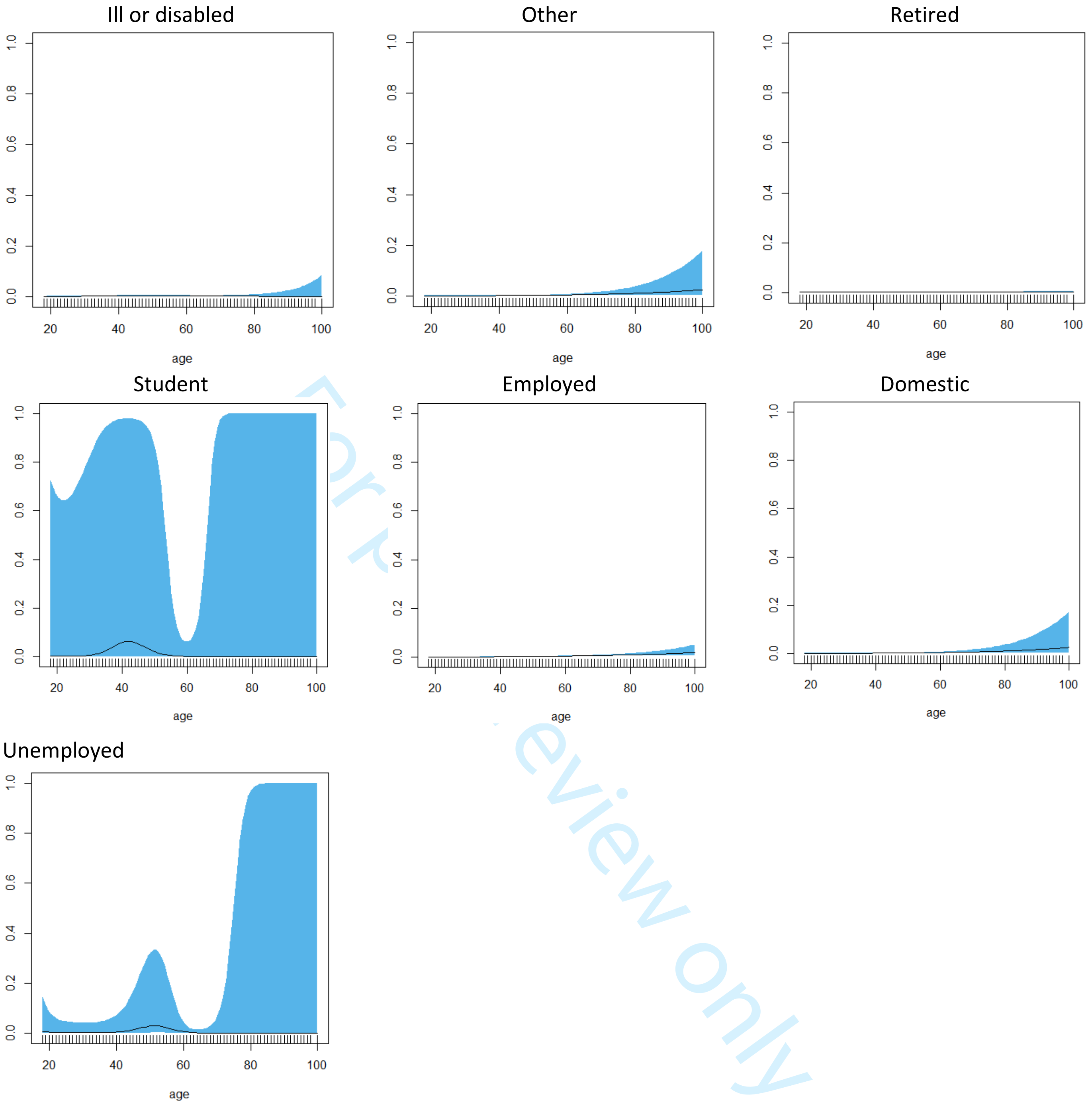

For peer review only

Figure SM6: Non-parametric estimation of the effect of age on likelihood of reported opioid use by income highest qualification in males

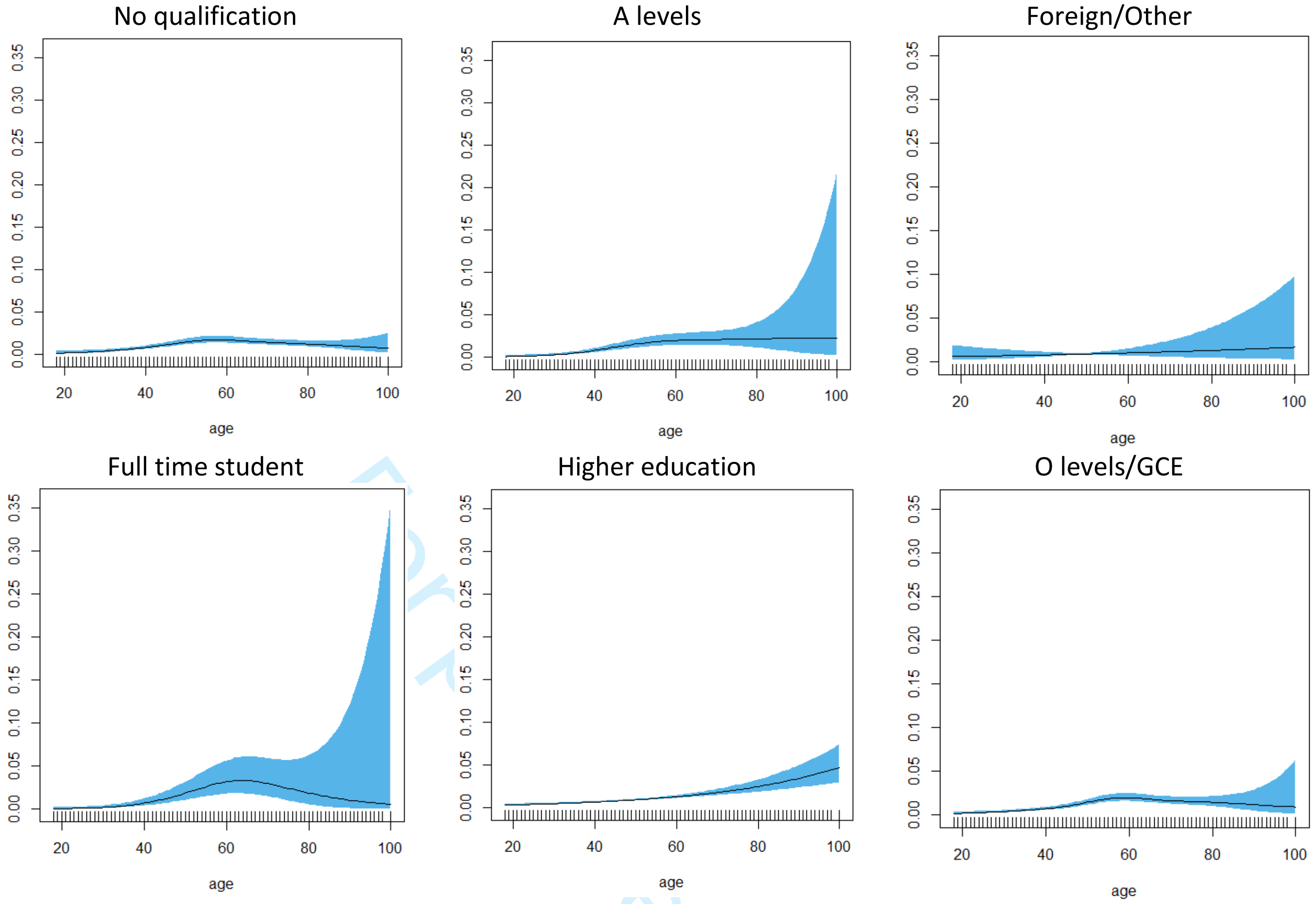

For peer review only

STROBE Statement—Checklist of items that should be included in reports of ***cohort studies***

	Item No	Recommendation	Page No
**Title and abstract**	1	(*a*) Indicate the study's design with a commonly used term in the title or the abstract	2
		(*b*) Provide in the abstract an informative and balanced summary of what was done and what was found	2
**Introduction**			
Background/rationale	2	Explain the scientific background and rationale for the investigation being reported	3
Objectives	3	State specific objectives, including any prespecified hypotheses	3-4
**Methods**			
Study design	4	Present key elements of study design early in the paper	4
Setting	5	Describe the setting, locations, and relevant dates, including periods of recruitment, exposure, follow-up, and data collection	4
Participants	6	(*a*) Give the eligibility criteria, and the sources and methods of selection of participants. Describe methods of follow-up	4
		(*b*) For matched studies, give matching criteria and number of exposed and unexposed	NA
Variables	7	Clearly define all outcomes, exposures, predictors, potential confounders, and effect modifiers. Give diagnostic criteria, if applicable	4
Data sources/ measurement	8*	For each variable of interest, give sources of data and details of methods of assessment (measurement). Describe comparability of assessment methods if there is more than one group	4
Bias	9	Describe any efforts to address potential sources of bias	4-5
Study size	10	Explain how the study size was arrived at	4 and 6
Quantitative variables	11	Explain how quantitative variables were handled in the analyses. If applicable, describe which groupings were chosen and why	4-5
Statistical methods	12	(*a*) Describe all statistical methods, including those used to control for confounding	5
		(*b*) Describe any methods used to examine subgroups and interactions	5
		(*c*) Explain how missing data were addressed	4
		(*d*) If applicable, explain how loss to follow-up was addressed	NA
		(*e*) Describe any sensitivity analyses	5
**Results**			
Participants	13*	(a) Report numbers of individuals at each stage of study—eg numbers potentially eligible, examined for eligibility, confirmed eligible, included in the study, completing follow-up, and analysed	5
		(b) Give reasons for non-participation at each stage	4-5
		(c) Consider use of a flow diagram	NA
Descriptive data	14*	(a) Give characteristics of study participants (eg demographic, clinical, social) and information on exposures and potential confounders	5-6
		(b) Indicate number of participants with missing data for each variable of interest	4-6
		(c) Summarise follow-up time (eg, average and total amount)	NA
Outcome data	15*	Report numbers of outcome events or summary measures over time	5

For peer review only

Main results	16	(*a*) Give unadjusted estimates and, if applicable, confounder-adjusted estimates and their precision (eg, 95% confidence interval). Make clear which confounders were adjusted for and why they were included	6-7
		(*b*) Report category boundaries when continuous variables were categorized	4
		(*c*) If relevant, consider translating estimates of relative risk into absolute risk for a meaningful time period	NA
Other analyses	17	Report other analyses done—eg analyses of subgroups and interactions, and sensitivity analyses	SM
**Discussion**			
Key results	18	Summarise key results with reference to study objectives	8-9
Limitations	19	Discuss limitations of the study, taking into account sources of potential bias or imprecision. Discuss both direction and magnitude of any potential bias	9
Interpretation	20	Give a cautious overall interpretation of results considering objectives, limitations, multiplicity of analyses, results from similar studies, and other relevant evidence	9
Generalisability	21	Discuss the generalisability (external validity) of the study results	9
**Other information**			
Funding	22	Give the source of funding and the role of the funders for the present study and, if applicable, for the original study on which the present article is based	10

*Give information separately for exposed and unexposed groups.

Note: An Explanation and Elaboration article discusses each checklist item and gives methodological background and published examples of transparent reporting. The STROBE checklist is best used in conjunction with this article (freely available on the Web sites of PLoS Medicine at http://www.plosmedicine.org/, Annals of Internal Medicine at http://www.annals.org/, and Epidemiology at http://www.epidem.com/). Information on the STROBE Initiative is available at http://www.strobe-statement.org.

For peer review only

For peer review only - http://bmjopen.bmj.com/site/about/guidelines.xhtml

BMJ Open

Examining non-linearity in the association between age and reported Opioid Use, in different socioeconomic strata: Cohort Study Using Health Survey for England Waves from 1997 to 2014

Journal:	*BMJ Open*
Manuscript ID	bmjopen-2021-057428.R2
Article Type:	Original research
Date Submitted by the Author:	19-Dec-2022
Complete List of Authors:	Nowakowska, Magdalena; The University of Manchester, Centre for Primary Care and Health Services Research<br>Zghebi, Salwa; The University of Manchester, Centre for Primary Care and Health Services Research<br>Chen, Li-chia; The University of Manchester, Division of Population Health, Health Services Research and Primary Care; School of Health Sciences; Faculty of Biology, Medicine and Health<br>Kontopantelis, Evangelos; The University of Manchester, Division of Informatics, Imagine and Data Sciences
**Primary Subject Heading**:	Epidemiology
Secondary Subject Heading:	Health services research, Epidemiology, Public health
Keywords:	EPIDEMIOLOGY, PAIN MANAGEMENT, SOCIAL MEDICINE

SCHOLARONE™
Manuscripts

I, the Submitting Author has the right to grant and does grant on behalf of all authors of the Work (as defined in the below author licence), an exclusive licence and/or a non-exclusive licence for contributions from authors who are: i) UK Crown employees; ii) where BMJ has agreed a CC-BY licence shall apply, and/or iii) in accordance with the terms applicable for US Federal Government officers or employees acting as part of their official duties; on a worldwide, perpetual, irrevocable, royalty-free basis to BMJ Publishing Group Ltd ("BMJ") its licensees and where the relevant Journal is co-owned by BMJ to the co-owners of the Journal, to publish the Work in this journal and any other BMJ products and to exploit all rights, as set out in our licence.

The Submitting Author accepts and understands that any supply made under these terms is made by BMJ to the Submitting Author unless you are acting as an employee on behalf of your employer or a postgraduate student of an affiliated institution which is paying any applicable article publishing charge ("APC") for Open Access articles. Where the Submitting Author wishes to make the Work available on an Open Access basis (and intends to pay the relevant APC), the terms of reuse of such Open Access shall be governed by a Creative Commons licence – details of these licences and which Creative Commons licence will apply to this Work are set out in our licence referred to above.

Other than as permitted in any relevant BMJ Author's Self Archiving Policies, I confirm this Work has not been accepted for publication elsewhere, is not being considered for publication elsewhere and does not duplicate material already published. I confirm all authors consent to publication of this Work and authorise the granting of this licence.

For peer review only

Examining non-linearity in the association between age and reported Opioid Use, in different socioeconomic strata: Cohort Study Using Health Survey for England Waves from 1997 to 2014

Magdalena Nowakowska,[1,2,3] Salwa S Zghebi,[1,4] Li-Chia Chen,[5] Evangelos Kontopantelis,[1,2]

1. NIHR School for Primary Care Research; Centre for Primary Care and Health Services Research; Manchester Academic Health Science Centre (MAHSC), University of Manchester, M13 9PL, Manchester, UK
2. Division of Data Informatics, Imaging and Data Sciences; School of Health Sciences; Faculty of Biology, Medicine and Health; Manchester Academic Health Science Centre (MAHSC), University of Manchester, M13 9PL, Manchester, UK
3. NIHR Greater Manchester Patient Safety Translational Research Centre; University of Manchester, M13 9PL, Manchester, UK
4. Division of Population Health, Health Services Research and Primary Care; School of Health Sciences; Faculty of Biology, Medicine and Health; Manchester Academic Health Science Centre (MAHSC), University of Manchester, M13 9PL, Manchester, UK
5. Division of Pharmacy and Optometry; School of Health Sciences; Faculty of Biology, Medicine and Health; Manchester Academic Health Science Centre (MAHSC), University of Manchester, M13 9PL, Manchester, UK

Corresponding Author
Evangelos Kontopantelis
Suite 5, 5th floor, Williamson Building,
176 Oxford Road, M13 9PL Manchester
e.kontopantelis@manchester.ac.uk

For peer review only - http://bmjopen.bmj.com/site/about/guidelines.xhtml

Word count: 2,834
Number of tables: 2
Number of figures: 3

For peer review only - http://bmjopen.bmj.com/site/about/guidelines.xhtml

ABSTRACT

Background: Age and socioeconomic status (SES) predict several health-related outcomes, including prescription opioid use. Contrasting findings from previous literature found higher prevalence of opioid use in both people over 65 years old and the working-age population of 35-55 years old. This study aimed to analyse if the association between age and opioid use is nonlinear and differs in adults with different SES levels.

Methods: This cohort study utilised the Health Survey for England waves 1997 to 2014 data to investigate the shape of the correlation between reported opioid use and income decile, employment status, and educational level. A semi-parametric generalised additive model (GAM) was employed, so that linearity of correlation was not assumed. The shape of the relationship was assessed using the effective degrees of freedom (EDF).

Results: Positive correlation between age and reported opioid use, more linear in people in the highest income decile (EDF: 1.01M, $p<0.001$) and higher education (EDF: 2.03, $p<0.001$) was observed. In people on lower income and with lower levels of education, the probability of reported opioid use peaks was at around 40 to 60 years old and slowly decreased after that. Higher income decile and higher levels of education were predictors of a lower probability of reported opioid use (OR: 0.27, 95%CI: 0.21 to 0.36 and OR: 0.48, 95%CI: 0.41 to 0.57 respectively). There was no statistically significant difference in opioid use between employed and unemployed people.

Conclusion: The relationship between age and the probability of prescribed opioid use varies greatly across different income and educations strata, highlighting different drivers in opioid prescribing across population groups. More research is needed into exploring patterns in opioid use in older people, particularly from disadvantaged socioeconomic backgrounds.

For peer review only - http://bmjopen.bmj.com/site/about/guidelines.xhtml

Strengths and Limitations of this Study:

- The use of Generalised Additive Models to investigate the relationship between age and reported opioid use within different socioeconomic strata allowed exploration of the correlation without the assumption of linearity
- Due to limitations in recorded data, the distinction between type, strengths and duration of opioids was not accounted for
- Survey data used in this study is sensitive to self-reporting bias
- The low prevalence of opioid users limited the number of covariates used in the statistical model

INTRODUCTION

Opioid painkillers are effective analgesics that can provide crucial pain relief for patients with acute or cancer pain, which are often still used for the treatment of chronic non-cancer pain, although evidence for the latter indicate higher risk of short-term harms and limited or no benefits compared to nonopioid therapy.[1] Extensive research suggests a dose and potency dependent association between prescribed opioid use and several adverse severe outcomes in patients who persistently use them.[2,3] Prescribed opioid use, as well as the associated adverse effects, varies across individuals and communities.[4,5] A growing body of academic literature focuses on the biological, psychosocial, and institutional factors that influence the likelihood of prescribed opioid use. Socioeconomic status (SES), which can be defined through several measures, including income, occupational status, and education, was found to be closely associated with opioid prescribing[5] as well as female gender,[6] ethnicities,[7] and presence of psychological comorbidities.[8] Age is one of the strongest predictors of health and health outcomes, and the factor most commonly included in predictive models. However, the association between age and prescribed opioid use is not necessarily linear.

Older people are more likely to experience complex health problems and often in need of multiple prescribed medications.[9] Several studies found that older people are more likely to use opioid analgesia.[10] This could be partially explained by the opioids' recommended indications in palliative care and the higher prevalence of painful conditions in older people.[11] In contrast, some studies observed a higher prevalence of prescribed opioid use in 35-49 year olds.[12] Working-age adults may be more likely to be exposed to trauma and experience occupation-related pain. In a UK-based study of prescribed opioid use, the highest proportion of new users of weak and moderate opioids were aged 35 to 54 years old, whereas over 50% of new users of strong opioids were 75 years and older.[13]

The relationship between age and opioid analgesia need is complex and influenced by many factors, including race, underlying mental health conditions and socioeconomic circumstances. A nearly 300% difference in opioid prescription prevalence was observed across the race/ethnicity-income gradient in the US.[14] In Sweden, the highest risk of prescribed opioid use was observed in medium-income women aged 65 years or older, living alone and with psychological distress; whereas the lowest risk was observed in low-income men aged 18-44, living alone and without psychological distress.[15] Socioeconomic status, including income, employment status and education level, can determine persons living and working conditions, access and interactions with the healthcare system, and overall health status, changing personal health and opioid-related life trajectories. This study hypothesises that the association between age and reported prescribed opioid use varies by SES level. We used generalised additive models to examine this hypothesis, a regression analysis method that allows exploration of the non-linearity of the relationship between age and prescribed opioid use within groups of different income, employment status, and education levels. Different associations between

For peer review only - http://bmjopen.bmj.com/site/about/guidelines.xhtml

age and opioid prescribing, across socio-economic strata, would potentially highlight the presence of different health needs and drivers for prescribing. This would have implications for research, since we would need to understand the underlying cause or causes of this variation, to inform policy and improve patient care.

METHODS

Data Source

This cohort study used the Health Survey for England (HSE), a national annual survey designed to monitor the nation's health and care trends.[16] Participants were offered a nurse visit during which several questions about medication use and measurements such as height and weight are recorded. Data on opioid use was available for extraction from surveys conducted between 1997 and 2014, and therefore, these data were analysed in this study. The HSE surveys are reviewed yearly by the Research Ethics Committee (East Midlands - Nottingham 2), and informed consent was obtained from all participants. All data used in this analysis is freely available and no further approvals are required.[17]

Study Population

Participants in the HSE are randomly chosen, for each wave, from all private households' addresses in England. Participants aged 18 and older with no reported diagnosis of cancer who completed the nurse interview part of the survey were included in the study. At a number of annual waves (1997-2014), a boost sample of a population with specific characteristics was collected. To minimise bias, we excluded the boost sample of people living in care homes which were included in the 2000- and 2005-year waves. The ethnic minority boost sample included at waves 1999 and 2004 was included in the analysis.

Definition of Opioid Use

Participants were asked if they take any prescribed medications and, if yes, permission was sought for a nurse to see its container and to record the name of the medication. Up to 22 prescribed medications were recorded using the British National Formulary (BNF) Codes. Medications coded as opioid analgesics (BNF code 40702) were included in this study. If at least on opioid analgesic was recorded, participant was coded as opioid users, and otherwise, they were coded as not an opioid users.

Socioeconomic Status Measures

Socioeconomic status was measured using three commonly used indicators: income, employment status, and education level. Income was measured using equalised income variable deciles from lowest to highest. This measure considers different financial resource requirements of different households by adjusting total household income for the number of adults and children in the household. Employment was categorised using participants' reported activity in the week before the interview. Education was categorised using participants' reported highest qualification achieved at the time of the interview. People with missing employment or education data were excluded from the main analysis due to small numbers (n=45; 0.04% and n=105; 0.08%, respectively). A larger number of people had missing income information (n=19,007; 15.2%); therefore, a separate category of "missing income" was included to investigate if they represent a different population.

Statistical Analysis

Descriptive statistics for people with and without reported opioid use were calculated. To analyse the effect of age on the likelihood of reported opioid use in groups of different socioeconomic status, a semi-parametric generalised additive model (GAM) was implemented in the following form:

For peer review only - http://bmjopen.bmj.com/site/about/guidelines.xhtml

$$\log\left(\frac{P(Y=1 \mid X=x)}{P(Y=0 \mid X=x)}\right) = \alpha + \beta X + f(age)(z_i)$$

In the parametric portion of the model, α is the intercept and β is the vector of parameters associated with a set of explanatory variables X. The results are presented in odds ratios (ORs) and the corresponding 95% confidence intervals (95%CI).

The non-parametric portion of the model is formed by the smoothing function f for age which can vary for each category of SES measure zi. GAM is an extension of the commonly used Generalised Linear Model (GLM), which relaxes the hypothesis that the relationship between predictor and outcome variable is linear.[18] Instead, the relationship is modelled using smooth functions which can take any shape. Varying the shape of the smooth function by category of SES allows better interpretation of the relationship between age and opioid use in groups with different income, employment status, and education levels. This relationship can be interpreted using the effective degrees of freedom (EDF) which can indicate whether the relationship is linear (EDF=1) or non-linear (EDF>1). Furthermore, graphical visualisation of the relationship is provided to aid the interpretation. Models were controlled for available demographics: sex and ethnicity.

As a sensitivity analysis, all analyses were performed separately for females and males to investigate if different patterns emerge. These analyses showed no noticeable difference and are presented in the Supplementary Material (Tables SM1 and SM2, figures SM1-SM6). All analyses were performed using R version 3.6.3, and GAM estimations were performed using the *mgcv* package, with thin plate regression splines, for modelling the smooth term in age.[19] The number of basis dimensions k was automatically selected through the $gam()$ function and the adequacy of k was assessed.

Patient and Public Involvement

No patients were involved in the design, implementation or writing of this paper. The results will be disseminated to the appropriate audience.

RESULTS

In total, 124,740 people met the inclusion criteria, with 2,470 (1.98%) reporting opioid use across all waves (Table 1). The mean age of opioid users was higher than non-users (59.00 ± 15.38 vs. 48.47 ± 17.75). A higher proportion of females and white people were opioid users than males and people of any other ethnicity (2.23% vs 1.67% and 2.13% vs. 0.86%, respectively). After adjusting for all covariates, in all models with the three SES indicators, females, white people, and people living in urban areas were more likely to report opioid use (Table 2).

For peer review only - http://bmjopen.bmj.com/site/about/guidelines.xhtml

Table 1: Characteristics of all eligible participants from HSE 1997-2014

	Opioid non-users	Opioid users
Number (%)	122,270 (98.02)	2,470 (1.98)
Age, mean (± SD)	48.47 (±17.75)	59.00 (±15.38)
Gender		
Female, N (%)	67,584 (97.77)	1,544 (2.23)
Male, N (%)	54,686 (98.33)	926 (1.67)
Ethnicity		
White	107,797 (97.87)	2,343 (2.13)
Non white	14,390 (99.14)	125 (0.86)
Income decile		
1 – lowest	10,300 (97.41)	274 (2.59)
2	10,288 (97.30)	286 (2.71)
3	10,218 (96.63)	356 (3.37)
4	10,272 (97.15)	301 (2.85)
5	10,342 (98.34)	231 (2.18)
6	10,398 (98.74)	175 (1.66)
7	10,440 (98.74)	133 (1.26)
8	10,475 (99.07)	98 (0.93)
9	10,461 (98.94)	112 (1.06)
10 - Highest	10,487 (99.19)	86 (0.81)
Income missing	18,589 (97.80)	418 (2.20)
Employment Status		
Student	4,123 (99.66)	14 (0.34)
Employed	69,564 (99.33)	465 (0.66)
Unemployed	2,367 (99.25)	18 (0.75)
Ill or disabled	4,726 (86.87)	714 (13.13)
Retired	29,055 (96.46)	1,067 (3.54)
Domestic worker†	11,321 (98.56)	165 (1.44)
Other	1,071 (97.72)	25 (2.28)
Employment Missing	43 (95.55)	2 (4.44)
Education*		
Higher Education	36,026 (98.79)	441 (1.21)
A-levels	13,896 (98.71)	181 (1.29)
O-levels/GCSE	31,358 (98.16)	586 (1.83)
Foreign/other	3,624 (97.84)	80 (2.16)
No qualifications	31,238 (96.51)	1,130 (3.49)
Full time student	6,026 (99.19)	49 (0.81)
Education Missing	102 (97.14)	3 (2.86)

* The O- and A-Levels examination certificates are the secondary and pre-university credentials in England, Wales and Northern Ireland. The O Levels, or Ordinary Levels, typically represent a total of 11 years of study and mark the end of the secondary education cycle. A-levels, or Advanced level qualifications, are subject-based qualifications (leading to university, further study, training, or work), studied by students in Sixth Form, which refers to the last two years of secondary education (ages 16–18). A General Certificate of Secondary Education (GCSE) is a qualification normally taken by most UK students at the end of compulsory education.

† Any person engaged in domestic work within an employment relationship

For peer review only - http://bmjopen.bmj.com/site/about/guidelines.xhtml

Compared to people in the lowest income decile, people reporting income in the highest decile were significantly less likely to report opioid use (OR: 0.27, 95%CI: 0.21 to 0.36). Compared to people who were employed the week before the interview, unemployed people were not statistically different in the likelihood of reporting opioid use. However, as expected, those classified as ill or disabled were significantly more likely to report opioid use (OR: 16.50, 95%CI: 13.24 to 20.55). People performing domestic work were also more likely to report opioid use (OR: 1.60, 95%CI: 1.32 to 1.94). Those with higher education were less likely to report opioid use than those with no qualifications (OR: 0.48, 95%CI: 0.41 to 0.57).

Table 2: Semi-parametric generalised additive models for the odds of reported prescription opioid use with smooth function for the effect of age, varying by SES measure

	**Opioid use (Model including income**	**Opioid use (Model including employment status)**	**Opioid use (Model including education)**
**Parametric coefficients**	**OR (95% CI)**	**OR (95% CI)**	**OR (95% CI)**
Intercept	0.009 (0.007 to 0.011) ***	0.003 (0.002 to 0.003) ***	0.01 (0.007 to 0.011) ***
Female (ref: Male)	1.32 (1.21 to 1.43) ***	1.53 (1.40 to 1.67) ***	1.33 (1.22 to 1.44) ***
White (ref: Not white)	2.24 (1.86 to 2.70) ***	2.01 (1.67 to 2.42)***	1.94 (1.61 to 2.32) ***
Urban (ref: Rural)	1.27 (1.15 to 1.41) ***	1.20 (1.08 to 1.33) ***	1.29 (1.17 to 1.43) ***
Income Decile (ref: 1-lowest)			
2	0.93 (0.76 to 1.13)		
3	1.01 (0.82 to 1.24)		
4	0.80 (0.65 to 0.99) *		
5	0.55 (0.43 to 0.70) ***		
6	0.51 (0.40 to 0.64) ***		
7	0.38 (0.29 to 0.49) ***		
8	0.34 (0.26 to 0.44) ***		
9	0.40 (0.32 to 0.51) ***		
10 – Highest	0.27 (0.21 to 0.36) ***		
Income Missing	0.64 (0.53 to 0.77) ***		
Economic status (ref: Employed)			
Student		0.92 (0.36 to 2.35)	
Unemployed		1.61 (0.97 to 2.64)	
Ill or disabled		16.50 (13.24 to 20.55) ***	
Retired		1.60 (0.93 to 2.76)	
Domestic worker		1.60 (1.32 to 1.94) ***	
Other		3.14 (2.02 to 4.87) ***	
Education (ref: No qualifications)			
Higher Education			0.48 (0.41 to 0.57) ***
A-levels			0.57 (0.47 to 0.70) ***
O-levels/GCSE			0.71 (0.61 to 0.83) ***
Foreign/other			0.63 (0.43 to 0.91) *
Student			0.57 (0.44 to 0.82) **
**Non Parametric**	**EDF**	**EDF**	**EDF**
Income Decile:			
1 – Lowest	3.97 ***		
2	6.08 ***		
3	4.17 ***		

For peer review only - http://bmjopen.bmj.com/site/about/guidelines.xhtml

4	3.31 ***		
5	2.64 ***		
6	2.51 ***		
7	2.06 ***		
8	1.52 ***		
9	1.04 ***		
10 – Highest	1.01 ***		
Income Missing	3.30 ***		
Economic status:			
Employed		1.01 ***	
Student		1.002	
Unemployed		1.002 *	
Ill or disabled		3.12 ***	
Retired		4.99 **	
Domestic worker		2.74 ***	
Other		1.76 *	
Education:			
Higher Education			2.03 ***
A-levels			2.93 ***
O-levels/GCSE			3.30 ***
No qualifications			4.30 ***
Foreign/Other			1.01 **
Student			2.81 ***

OR – Odds Ratio; CI: Confidence Intervals; EDF - Estimated Degrees of Freedom
** p-value < 0.05*
*** p-value < 0.01*
**** p-value <0.001*

The effect of age on the likelihood of reporting opioid use was non-linear and significant in all income deciles as suggested by the statistically significant EDF. The higher the income decile, the relationship between age and probability of opioid use approached linear relationship with the EDF: 1.01, p<0.001 for highest income decile and EDF: 3.97, p<0.001 for lowest income decile. This suggests that in people with higher income, older people were more likely to report opioid use. However, in lower-income deciles, the relationship was non-linear. In particular, in people on the lowest income decile, the probability of opioid use increased with age until approximately 55 years old, after which it reduced (Figure 1).

Concerning employment status, age had a statistically significant association with reported opioid use in people who were employed (EDF: 1.01, p<0.001), ill or disabled (EDF: 3.12, p<0.001), domestic workers (EDF: 2.74, p<0.001), and with lower significance levels, retired (EDF: 4.99, p<0.01), unemployed (EDF: 1.002, p<0.05), and other economic statuses (EDF: 1.76, p<0.05). In people who were employed and unemployed, the relationship approached linearity (EDF: 1.01, p<0.001 and EDF: 1.002, p<0.05 respectively), whereas in people who were ill or disabled, the distribution was slightly skewed towards people between 40 and 60 years old, and in domestic workers, the likelihood peaked at around 60 years of age (Figure 2).

For all levels of education status, the effect of age on the odds of reporting opioid use was statistically significant. The effect was almost linear for people with foreign/other qualifications (Figure 3). In people with higher education, older age was associated with higher odds of reported opioid use. In contrast, in people with lower qualifications (A-levels, O-levels/GCSE) the odds of reported opioid use

For peer review only - http://bmjopen.bmj.com/site/about/guidelines.xhtml

peaked around 60 years old slightly decrease after that. The odds of reported opioid use peaked earlier in people with no qualifications at around 50 years old.

DISCUSSION

This study found that the relationship between age and the likelihood of reported opioid use changes with different measures of socioeconomic status. In people with higher income and education, the relationship is more linear, increasing with age. In people on lower income with lower levels of education, the likelihood of reported opioid use peaks at around 40 to 60 years old and slowly decreases after that. Consistently with previous research, females, white people, those living in urban areas, and those with lower levels of SES were more likely to report opioid use. The difference between employed and unemployed people was not statistically significant regarding the likelihood of reported opioid use. In both groups, the association with age was linear and increasing with age.

Existing studies found mixed results regarding the correlation between age and use of opioids; some studies found a higher prevalence in older populations[10] whereas others in the working-age population.[12] This study shows that SES may contribute to the non-linear relationship between age and opioid use. Shaw, Roelofs, and Punnett (2017)[20] suggest two pathways in which working conditions, strongly correlated with income, related to opioid use. The first suggests that physical injury at work or cumulative trauma from strenuous labour leads to pain and the use of painkillers. The second relates to psychological stress from unmanageable job demands like time pressure or economic insecurity and job instability, leading to depression and anxiety, which may correlate with chronic opioid use. These adverse working conditions are expected in people with lower-paying jobs, explaining the observed higher prevalence of reported opioid use in working-age people with lower SES. Furthermore, people experiencing disability or illness who are in a greater need of opioid treatment are more likely to be on lower income due to difficulties sustaining employment, regardless of age. Interestingly, we observe a reversal in the relationship between rurality and opioid use, compared to what has been reported in the USA. [21,22] However, this is not unexpected considering the different socioeconomic make-up in the rural USA and rural UK, with rural UK areas being generally much more affluent than urban conurbations, contrary to the situation in the USA.[23]

Our findings show an increase in reported opioid use with age in people with higher income and higher education. Reported opioid use in people with lower income, and also in people with no higher education, decreased after it peaked around 40 to 60 years of age. Two possible explanations have been identified in the literature. Firstly, previous research found that the prevalence of musculoskeletal pain increases up to approximately 65 years old, after which it declines.[24] This has been linked to the decline of the adverse physical and mental effects of the workplace at the age of retirement and the lower prevalence of several diseases in surviving populations as people with chronic diseases tend to die early.[25] Furthermore, a review of pain management in the elderly showed that pain in the elderly might be untreated and misdiagnosed.[26] Conversely, a higher prevalence of opioid use would be expected in older people due to more complex health needs and the use of opioid analgesia in palliative care. Further research is needed to establish if older people from lower socioeconomic backgrounds are more likely to experience difficulties accessing adequate analgesia.

Strengths and limitations

The statistical techniques used in this study allowed modelling the relationship between age and odds of reported opioid use in a non-linear manner and present results separately for people with different

For peer review only - http://bmjopen.bmj.com/site/about/guidelines.xhtml

levels of SES. Various methods can examine the interaction between two variables, each with different strengths and limitations. A common approach includes an interaction term where the effect of a covariate of interest can vary depending on another variable.[27] Alternatively, multilevel models allow parameters of interest, including the intercept and the slope of the regression line, to vary across more than one level.[28] However, these methods assume a linear relationship. Age can be modelled as categorical, but this can lead to complex models where multiple age categories interact with multiple SES categories, and information is difficult to interpret. Other techniques, including several machine learning methods, can produce highly accurate but difficult to interpret estimations. This study employed generalised additive models, a regression analysis method that allows exploration of the non-linearity of the relationship between age and opioid use within groups of different income, employment status, and education levels. To our knowledge, this is the first study to employ this technique to explore the relationship between age and prescription opioid use in England.

However, some limitations should be acknowledged. Firstly, the relationship between age and opioid use may be specific to different opioid strengths and durations. The data records in HSE did not capture the type of prescribed opioids; hence they did not adjust for the opioid type strength in the analysis. Secondly, opioid use was assessed based on self-reported data, which may be susceptible to recall and social desirability bias. However, this risk of bias was reduced by the involvement of qualified nurses in collecting medication data. Thirdly, the low prevalence of opioid use meant that a limited number of covariates was included in the model. Some variables, such as the presence of chronic conditions, had to be excluded from the models, which could explain the different patterns of associations across socio-economic strata. Fourth, the HES, although considered representative of the English population,[16] does not include some subgroups of the population, who are not living in private households, such as the homeless (who are at higher risk of opioid abuse) and those living in communal establishments (e.g. residential and nursing homes). However, less than 2% of the population of England live in communal establishments,[29] while less than 0.5% are homeless.[30]

Conclusion

Findings from this study suggest that, although the relationship between age and odds of opioid use in people with higher income and education approaches linearity, in people with lower SES, the probability of exposure peaks between 40 to 60 years old and decreases after that. This variability in the relationship between age and the probability of prescribed opioid use, highlights different drivers in opioid prescribing across different income and educations strata. More research is needed into exploring patterns in opioid use in older people, particularly from disadvantaged socioeconomic backgrounds.

For peer review only - http://bmjopen.bmj.com/site/about/guidelines.xhtml

Authors contributions

All authors contributed to the design of the study. MN prepared and analysed the data. MN wrote the manuscript and MN, SSZ, LCC, and EK all critically edited the manuscript. All authors approved the manuscript before submission.

Competing interest

MN, SSZ, LCC, and EK have no conflict of interest to declare.

Role of the funding source

This study was funded as part of a PhD studentship from the National Institute for Health Research (NIHR) School for Primary Care Research (SPCR) and NIHR Greater Manchester Patient Safety Translational Research Centre. The study represents independent research by the NIHR. The views expressed in this publication are those of the authors and not necessarily those of the National Health Service, the NIHR or the Department of Health and Social Care. The study funders had no role in the study design, data collection, analysis or interpretation, in the writing of the paper or in the decision to submit the paper for publication. MN had full access to all the data in the study and had final responsibility for the decision to submit for publication.

Data sharing

All data used in this study are publicly available from the sources cited in the manuscript.

Research Ethics Approval

This study involves analysis of anonymous and publicly available data. No ethical approval was sought or required for this study.

For peer review only

For peer review only - http://bmjopen.bmj.com/site/about/guidelines.xhtml

Figure 1: Non-parametric estimation of the effect of age on likelihood of reported opioid use by income decile

Figure 2: Non-parametric estimation of the effect of age on likelihood of reported opioid use by economic status

Figure 3: Non-parametric estimation of the effect of age on likelihood of reported opioid use by education status

For peer review only

For peer review only - http://bmjopen.bmj.com/site/about/guidelines.xhtml

REFERENCES

1 Chou R, Hartung D, Turner J, *et al.* Opioid Treatments for Chronic Pain. *AHRQ Publ No 20-EHC011* Published Online First: 2020. doi:https://doi.org/10.23970/AHRQEPCCER229.
2 Benyamin R, Trescot AM, Datta S, *et al.* Opioid complications and side effects. 2008. doi:10.36076/ppj.2008/11/s105
3 Moore RA, McQuay HJ. Prevalence of opioid adverse events in chronic non-malignant pain: systematic review of randomised trials of oral opioids. Arthritis Res. Ther. 2005;**7**:1–6. doi:10.1186/ar1782
4 Nowakowska M, Zghebi SS, Perisi R, *et al.* Association of socioeconomic deprivation with opioid prescribing in primary care in England: A spatial analysis. *J Epidemiol Community Health* 2021;**75**:128–36. doi:10.1136/jech-2020-214676
5 Macfarlane GJ, Beasley M, Jones GT, *et al.* The epidemiology of regular opioid use and its association with mortality: Prospective cohort study of 466 486 UK biobank participants. *EClinicalMedicine* 2020;**21**:100321. doi:10.1016/j.eclinm.2020.100321
6 Serdarevic M, Striley CW, Cottler LB. Sex differences in prescription opioid use. Curr. Opin. Psychiatry. 2017;**30**:238–46. doi:10.1097/YCO.0000000000000337
7 Ringwalt C, Roberts AW, Gugelmann H, *et al.* Racial Disparities Across Provider Specialties in Opioid Prescriptions Dispensed to Medicaid Beneficiaries with Chronic Noncancer Pain. *Pain Med (United States)* 2015;**16**:633–40. doi:10.1111/pme.12555
8 Davis MA, Lin LA, Liu H, *et al.* Prescription opioid use among adults with mental health disorders in the United States. *J Am Board Fam Med* 2017;**30**:407–17. doi:10.3122/jabfm.2017.04.170112
9 Hajjar ER, Cafiero AC, Hanlon JT. Polypharmacy in elderly patients. *Am J Geriatr Pharmacother* 2007;**5**:345–51. doi:10.1016/j.amjopharm.2007.12.002
10 Bedene A, Lijfering WM, Niesters M, *et al.* Opioid Prescription Patterns and Risk Factors Associated With Opioid Use in the Netherlands. *JAMA Netw open* 2019;**2**:e1910223. doi:10.1001/jamanetworkopen.2019.10223
11 Gibson SJ, Lussier D. Prevalence and Relevance of Pain in Older Persons. Pain Med. 2012;**13**:S23–6. doi:10.1111/j.1526-4637.2012.01349.x
12 Rasu RS, Knell ME. Determinants of opioid prescribing for nonmalignant chronic pain in US outpatient settings. *Pain Med (United States)* 2018;**19**:524–32. doi:10.1093/pm/pnx025
13 Jani M, Yimer BB, Sheppard T, *et al.* Time trends and prescribing patterns of opioid drugs in UK primary care patients with non-cancer pain: A retrospective cohort study. *PLoS Med* 2020;**17**:e1003270. doi:10.1371/journal.pmed.1003270
14 Friedman J, Kim D, Schneberk T, *et al.* Assessment of Racial/Ethnic and Income Disparities in the Prescription of Opioids and Other Controlled Medications in California. *JAMA Intern Med* 2019;**179**:469–76. doi:10.1001/jamainternmed.2018.6721
15 Persmark A, Wemrell M, Zettermark S, *et al.* Precision public health: Mapping socioeconomic disparities in opioid dispensations at Swedish pharmacies by Multilevel Analysis of Individual Heterogeneity and Discriminatory Accuracy (MAIHDA). *PLoS One* 2019;**14**:e0224008. doi:10.1371/journal.pone.0220322
16 Mindell J, Biddulph JP, Hirani V, *et al.* Cohort profile: The health survey for england. *Int J Epidemiol* 2012;**41**:1585–93. doi:10.1093/ije/dyr199
17 NHS Digital. Health Survey for England - NHS Digital.
18 Hastie T, Tibshirani R. Generalized additive models for medical research.
19 Wood Simon. Mixed GAM Computation Vehicle with Automatic Smoothness Estimation. 2021. doi:10.1201/9781315370279
20 Shaw WS, Roelofs C, Punnett L. Work environment factors and prevention of opioid-related deaths. *Am J Public Health* 2020;**110**:1235–41. doi:10.2105/AJPH.2020.305716
21 Palombi LC, St Hill CA, Lipsky MS, *et al.* A scoping review of opioid misuse in the rural United

For peer review only

States. *Ann Epidemiol* 2018;**28**:641–52.
22 Rigg KK, Monnat SM. Urban vs. rural differences in prescription opioid misuse among adults in the United States: Informing region specific drug policies and interventions. *Int J Drug Policy* 2015;**26**:484–91.
23 Rae A. Spatially Concentrated Deprivation in England: An Empirical Assessment. *Reg Stud* 2012;**46**:1183–99. doi:10.1080/00343404.2011.565321
24 Parsons S, Breen A, Foster NE, *et al.* Prevalence and comparative troublesomeness by age of musculoskeletal pain in different body locations. *Fam Pract* 2007;**24**:308–16. doi:10.1093/fampra/cmm027
25 Cimmino MA, Ferrone C, Cutolo M. Epidemiology of chronic musculoskeletal pain. 2011.
26 Noroozian M, Raeesi S, Hashemi R, *et al.* Pain: The neglect issue in old people's life. *Open Access Maced J Med Sci* 2018;**6**:1773–8. doi:10.3889/oamjms.2018.335
27 Kontopantelis E, Sperrin M, Mamas MA, *et al.* Investigating heterogeneity of effects and associations using interaction terms. J. Clin. Epidemiol. 2018;**93**:79–83. doi:10.1016/j.jclinepi.2017.09.012
28 Peugh JL. A practical guide to multilevel modeling. *J Sch Psychol* 2010;**48**:85–112. doi:10.1016/j.jsp.2009.09.002
29 Office of National Statistics. 2011 Census analysis: What Does the 2011 Census Tell Us About People Living in Communal Establishments. 2015.
30 Shelter. 274,000 people in England are homeless, with thousands more likely to lose their homes. 2021.

For peer review only

Figure 1: Non-parametric estimation of the effect of age on likelihood of reported opioid use by income decile

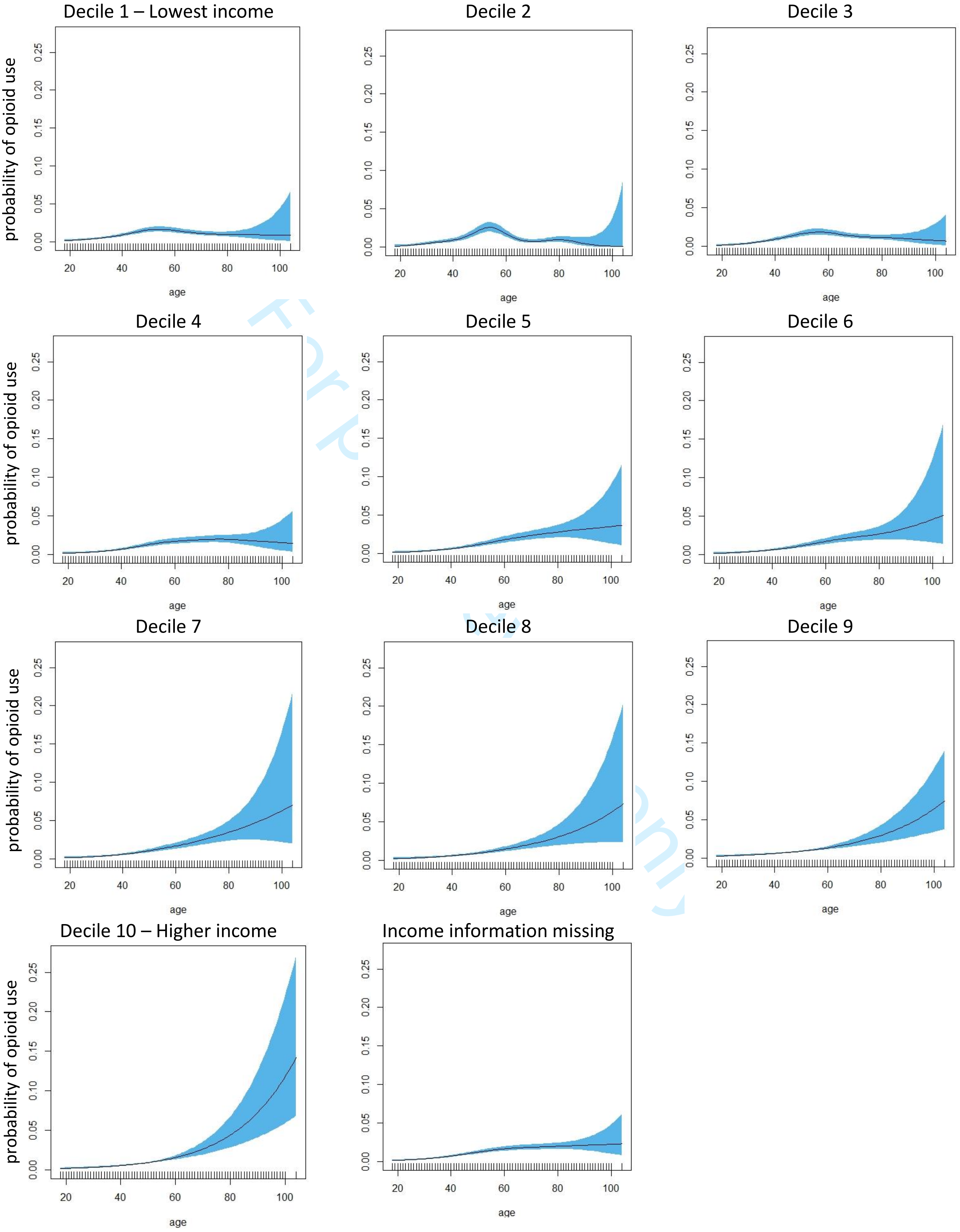

Figure 2: Non-parametric estimation of the effect of age on likelihood of reported opioid use by economic status

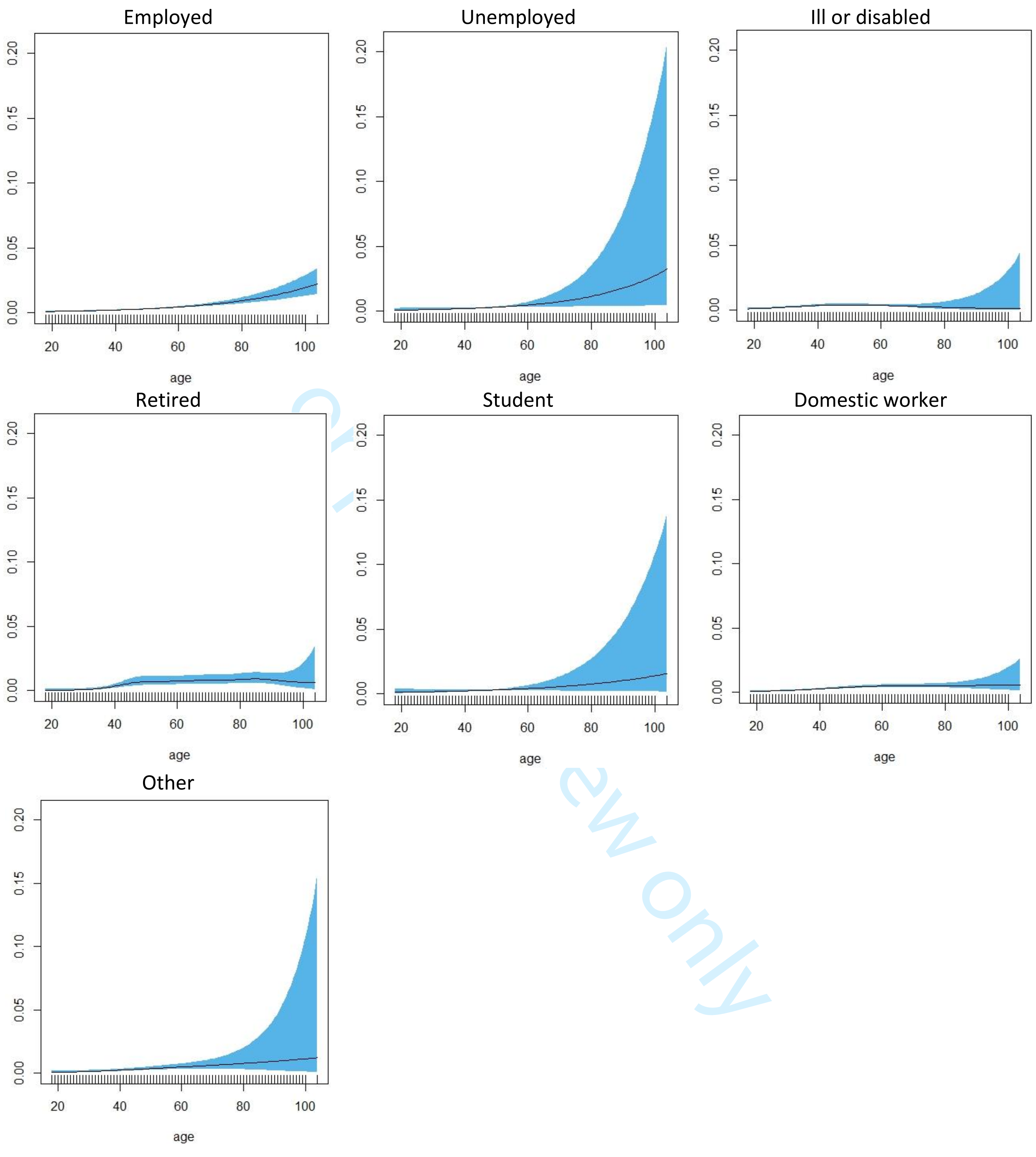

Figure 3: Non-parametric estimation of the effect of age on likelihood of reported opioid use by education status

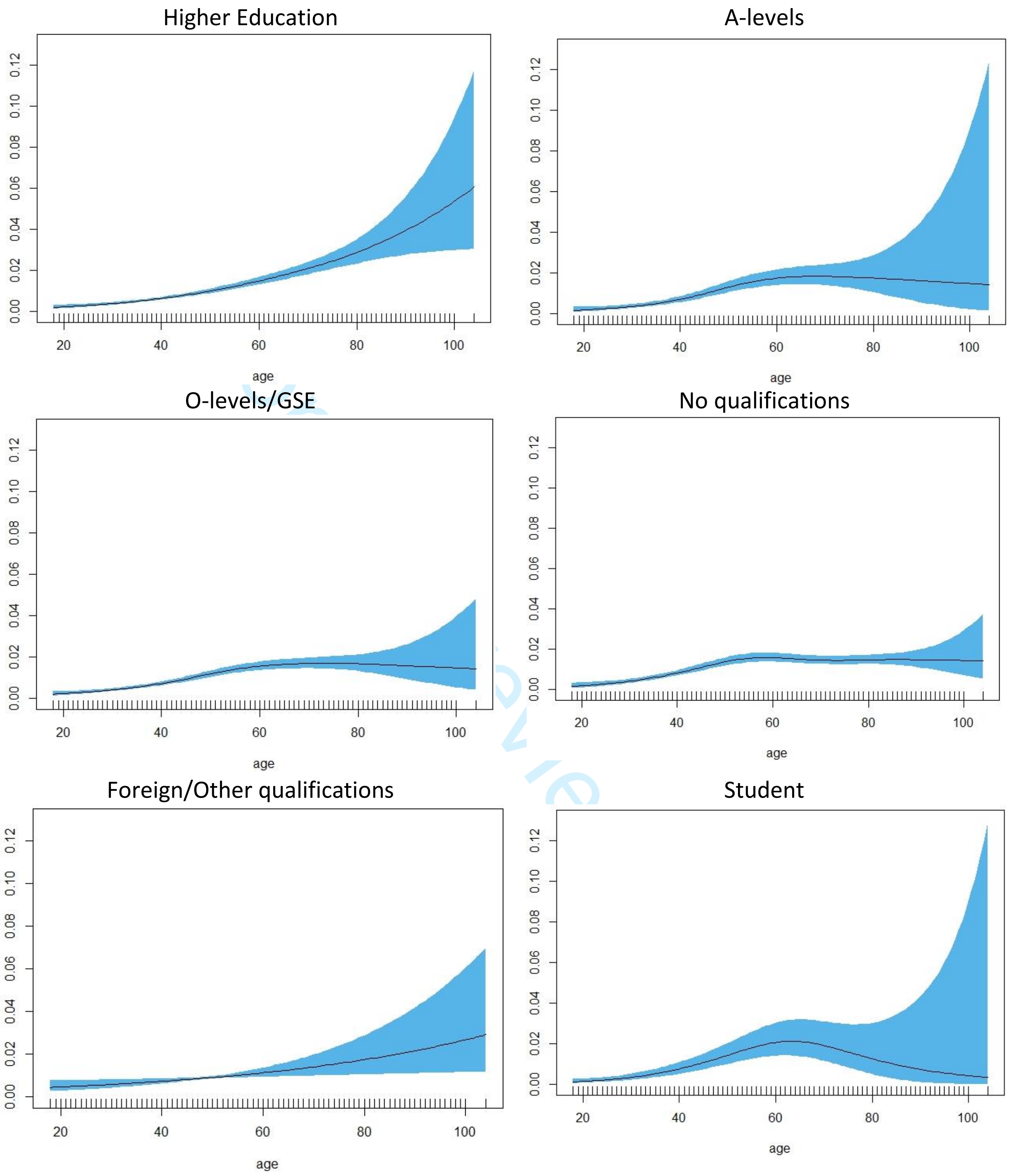

The nonlinear association between age and socioeconomic status on reported opioid use: cohort study using health survey for England waves from 1997 to 2014

Supplementary Materials: Sensitivity Analysis

For peer review only

Table SM1: Semi-parametric generalised additive models for the odds of reported prescription opioid use with smooth function for the effect of age, varying by SES measure in Females only

	Opioid use (Model including income	Opioid use (Model including employment status)	Opioid use (Model including education)
**Parametric coefficients**	**OR (95% CI)**	**OR (95% CI)**	**OR (95% CI)**
Intercept	0.01 (0.01:0.01) ***	0.004 (0.003:0.006) ***	0.012 (0.009:0.159) ***
White (ref: Not white)	2.11 (1.66:2.67) ***	1.91 (1.51:2.43)***	1.90 (1.50:2.41)***
Urban (ref: Rural)	1.30 (1.14:1.48) ***	1.23 (1.08:1.40)**	1.31 (1.14:1.49)***
Income Quantile (ref: 1-Lowest)			
Income 2	0.98 (0.81:1.19)		
Income 3	0.71 (0.57:0.87) ***		
Income 4	0.47 (0.38:0.59) ***		
Income 5 - Highest	0.41 (0.32:0.52) ***		
Income Missing	0.75 (0.60:0.93) **		
Economic Status (ref: Employed)			
Domestic		1.59 (1.29:1.97) ***	
Ill or disabled		17.52 ***(12.97:23.66)	
Other		3.04 (1.70:5.43)***	
Retired		1.75 (1.70:5.43)*	
Student		0.75 (0.19:2.80)	
Unemployed		1.78 (0.83:3.81)	
Education (ref: No qualification)			
Higher education			0.51 (0.41:0.62)***
A-levels			0.53 (0.41:0.69) ***
O-levels/GSE			0.71 (0.58:0.85)***
Foreign/Other			0.61 (0.39:0.94) *
Student			0.48 (0.31:0.75)**
**Non Parametric**	**EDF**	**EDF**	**EDF**
**Income Quantile**			
Income 1 - lowest	4.91 ***		
Income 2	3.52 ***		
Income 3	2.56 ***		
Income 4	1.12 ***		
Income 5 - highest	1.91 ***		
Income Missing	3.01 ***		

Employment status			
Domestic		2.669***	
Ill or disabled		2.626 *	
Other		1.428	
Retired		4.013 ***	
Student		1.000	
Unemployed		1.002	
Employed		1.003 ***	
Education			
Higher education			2.108***
A-levels			2.646***
O-levels/GSE			2.302***
No qualifications			3.631***
Foreign/Other			1.049*
FT Student			2.062*

Table SM2: Semi-parametric generalised additive models for the odds of reported prescription opioid use with smooth function for the effect of age, varying by SES measure in Males only

	**Opioid use (Model including income**	**Opioid use (Model including employment status)**	**Opioid use (Model including education)**
**Parametric coefficients**	**OR (95% CI)**	**OR (95% CI)**	**OR (95% CI)**
Intercept	0.01 (0.01:0.01) ***	0.003 (0.002:0.004)***	0.009 (0.006:0.13)***
White (ref: Not white)	2.48 (1.84:3.35) ***	2.17 (1.60:2.93)***	2.02***
Urban (ref: Rural)	1.24 (1.05:1.46) **	1.13 (0.96:1.34)	1.26**
Income Quintile (ref: 1-Lowest)	ref		
Income 2	0.85 (0.66:1.09)		
Income 3	0.34 (0.25:0.47) ***		
Income 4	0.24 (0.17:0.34) ***		
Income 5 - highest	0.30 (0.22:0.40) ***		
Income Missing	0.54 (0.41:0.71) ***		
Economic Status (ref: Employed))			
Domestic		2.82 (1.40:5.66)**	
Ill or disabled		15.51 (11.40:21.10)***	
Other		3.57 (1.89:6.74)***	
Retired		3.80 (2.66:5.43)****	
Student		0.12 (0.0002:89.32)	
Unemployed		0.33 (0.02:4.08)	
Education (ref: No qualification)			
Higher education			0.44 (0.34:0.57)***
A-levels			0.56 (0.40:0.79)***
O-levels/GSE			0.70 (0.54:0.91)**
Foreign/Other			0.65 (0.31:1.39)
Student			0.63 (0.39:1.06)
**Non Parametric**	**EDF**	**EDF**	**EDF**
**Income Quantile**			
Income 1 - lowest	4.73 ***		
Income 2	3.89 ***		
Income 3	2.69 ***		
Income 4	2.79 ***		
Income 5 - highest	1.13 ***		
Income Missing	2.89 ***		

For peer review only

Employment status			
Employed		1.300***	
Domestic		1.001*	
Ill or disabled		2.900**	
Other		1.002*	
Retired		1.002	
Student		2.742	
Unemployed		3.232	
Education			
Higher education			1.002***
A-levels			2.878***
O-levels/GSE			3.929***
No qualifications			3.695***
Foreign/Other			1.002
Student			2.741***

Figure SM1: Non-parametric estimation of the effect of age on likelihood of reported opioid use by income quantile in females

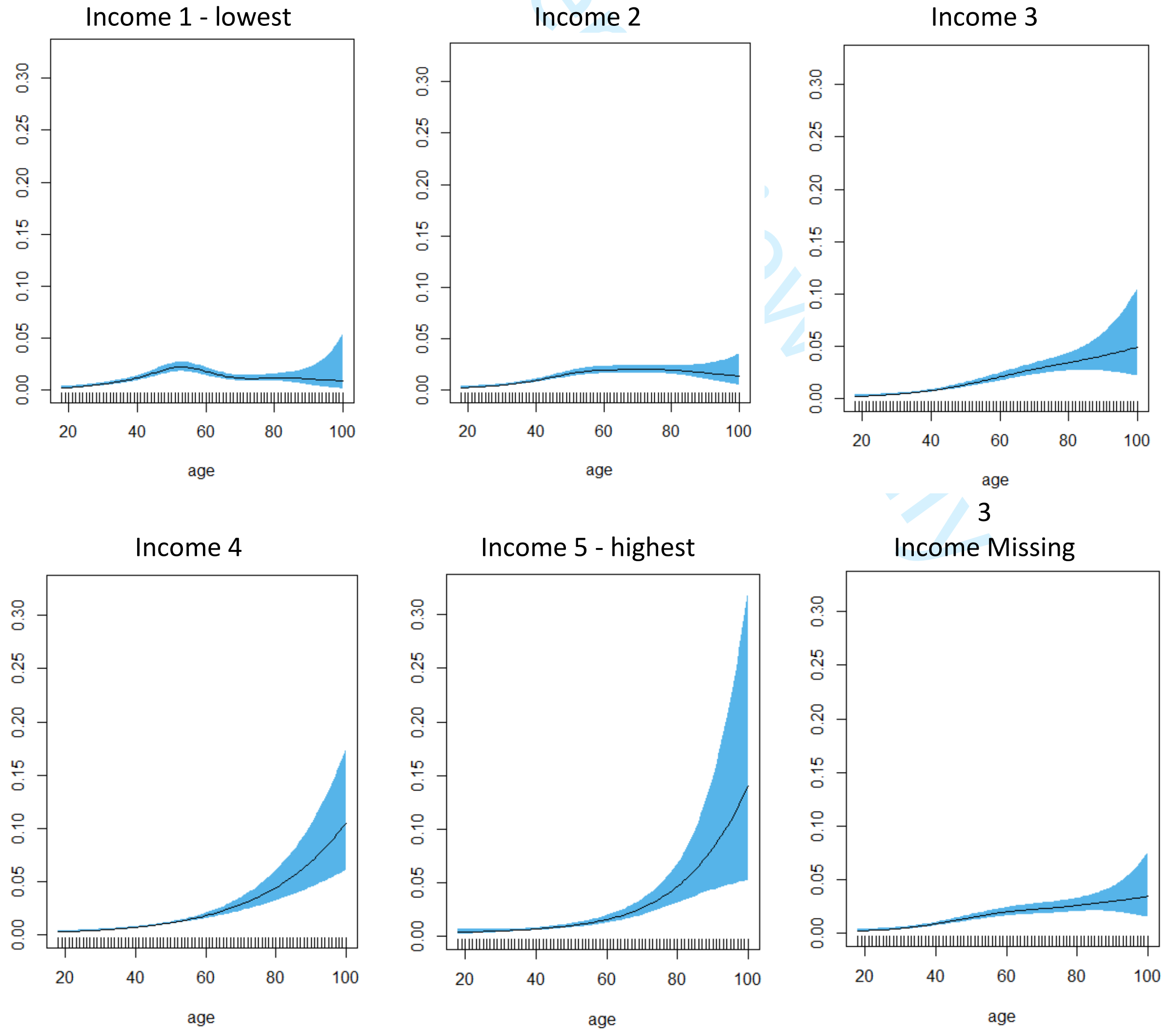

For peer review only

Figure SM2: Non-parametric estimation of the effect of age on likelihood of reported opioid use by employment status in females

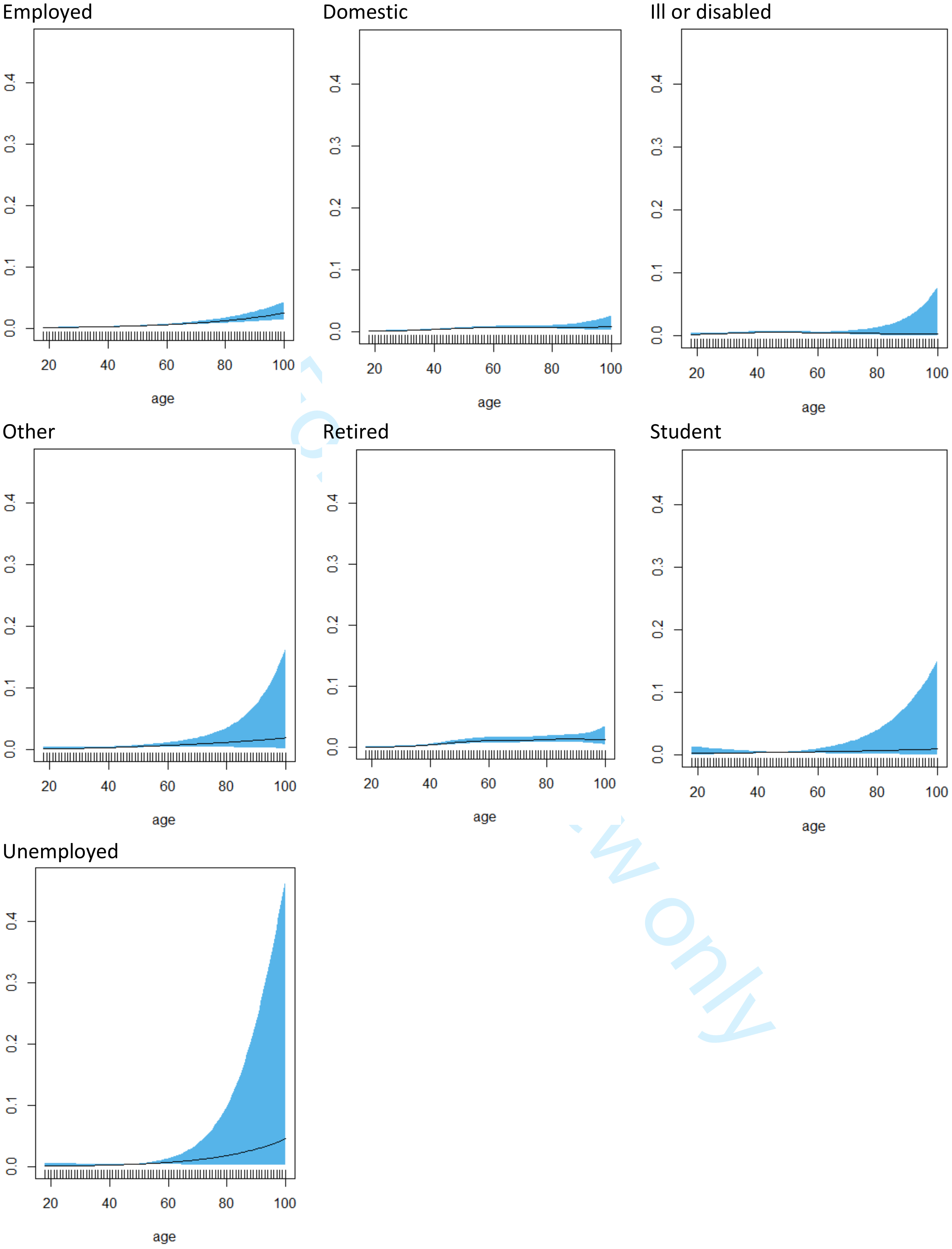

Figure SM3: Non-parametric estimation of the effect of age on likelihood of reported opioid use by income highest qualification in females

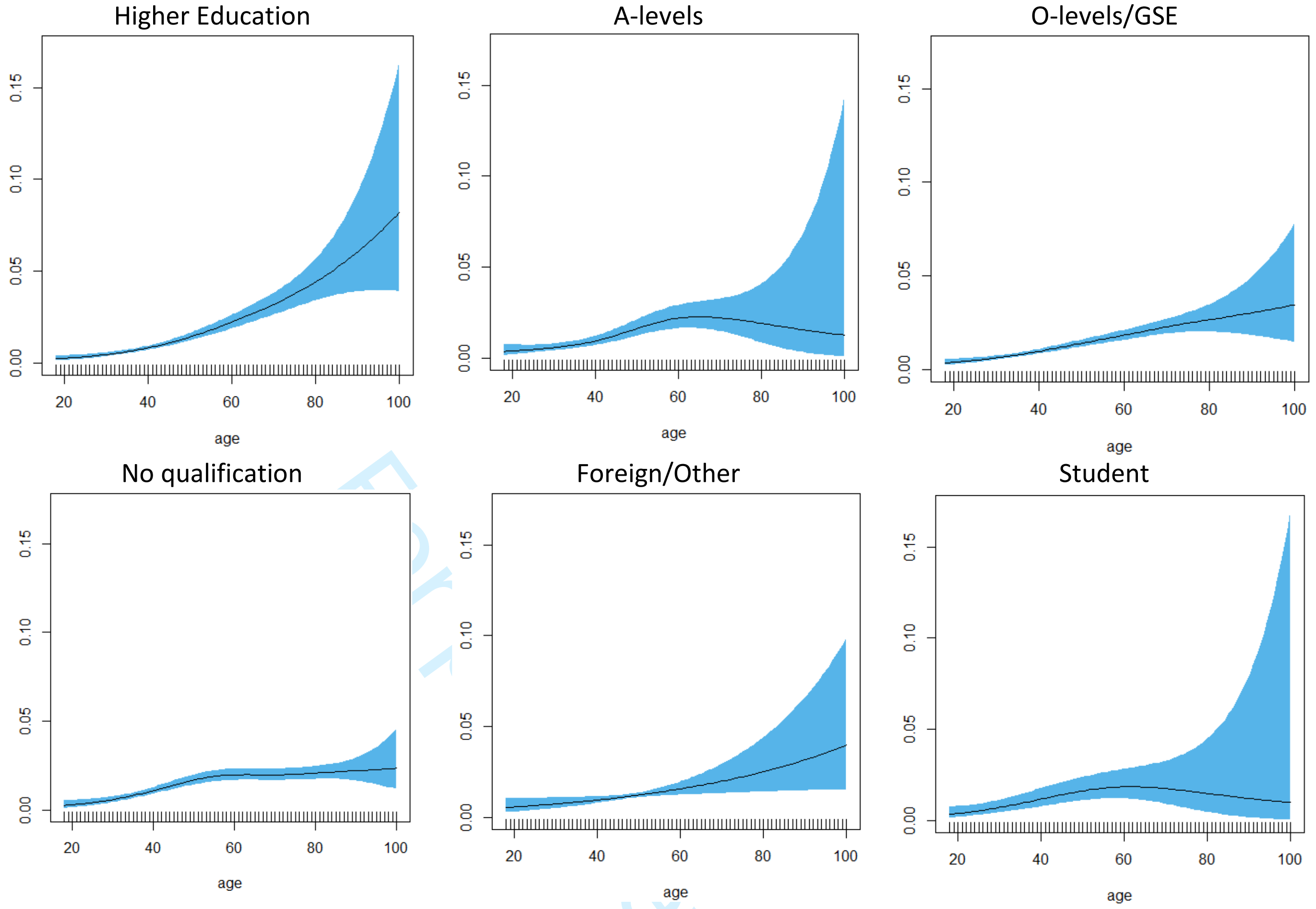

Figure SM4: Non-parametric estimation of the effect of age on likelihood of reported opioid use by income quantile in males

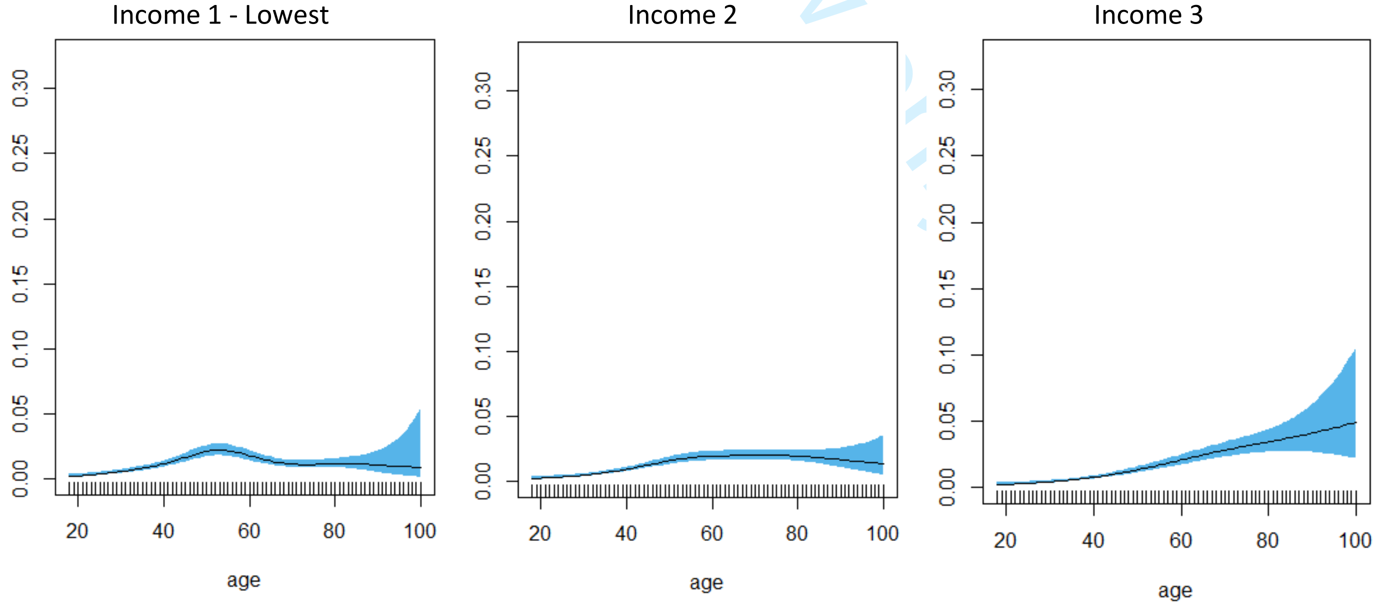

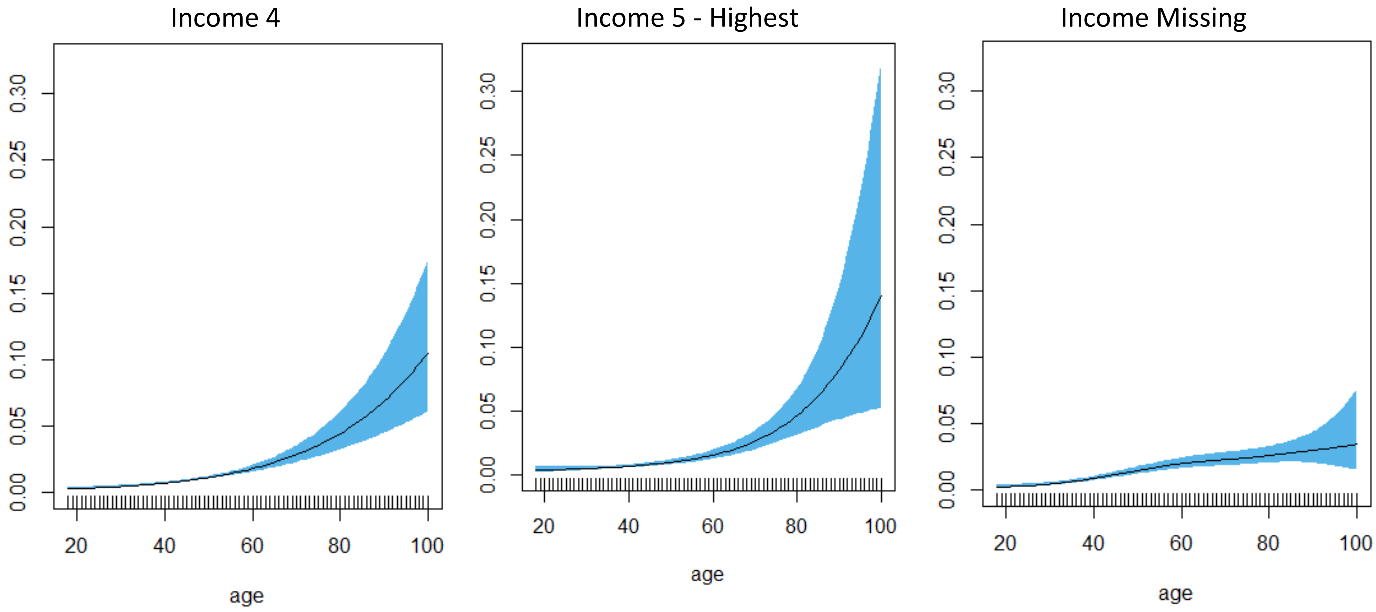

For peer review only

Figure SM5: Non-parametric estimation of the effect of age on likelihood of reported opioid use by employment status in males

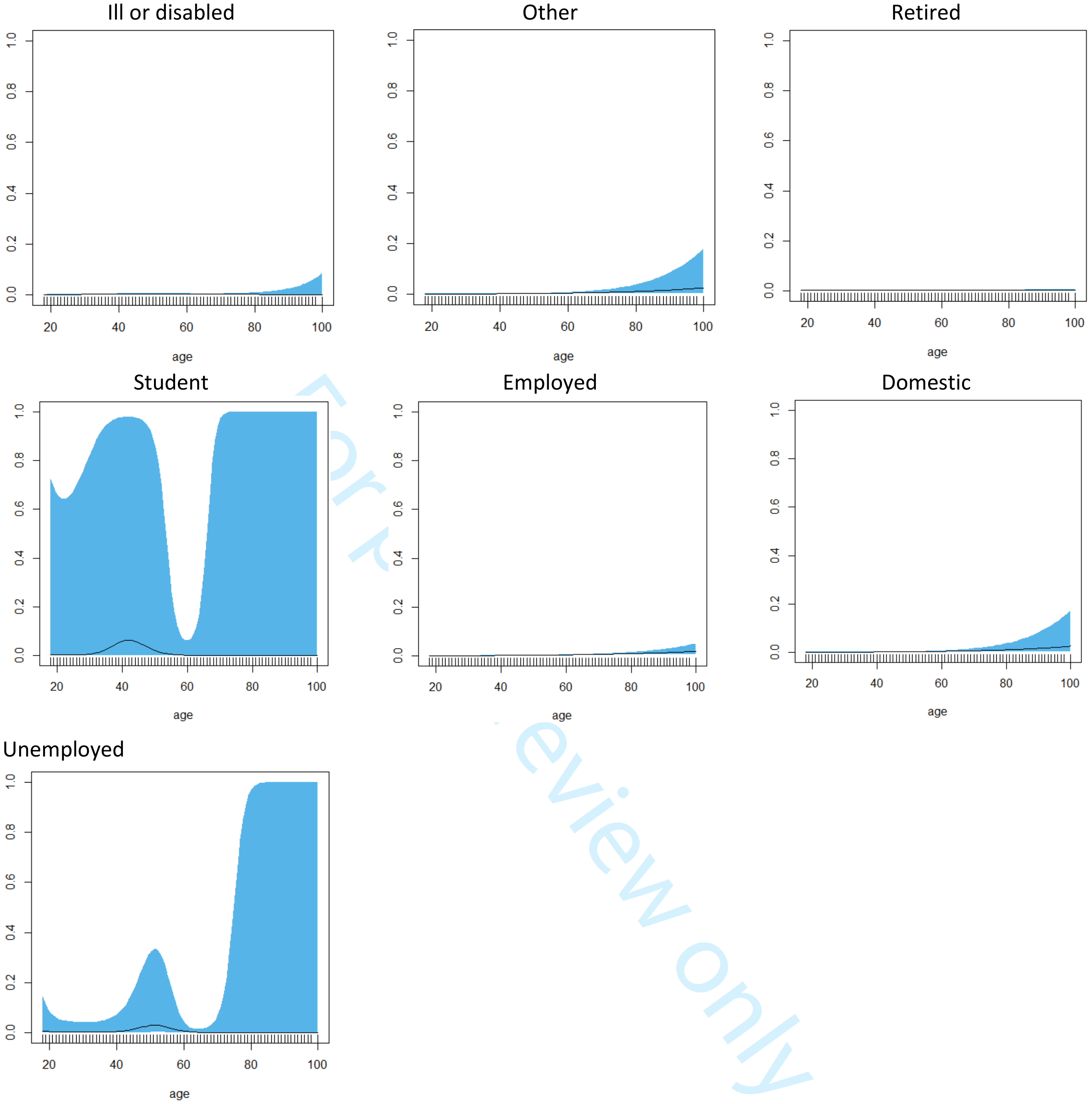

For peer review only

Figure SM6: Non-parametric estimation of the effect of age on likelihood of reported opioid use by income highest qualification in males

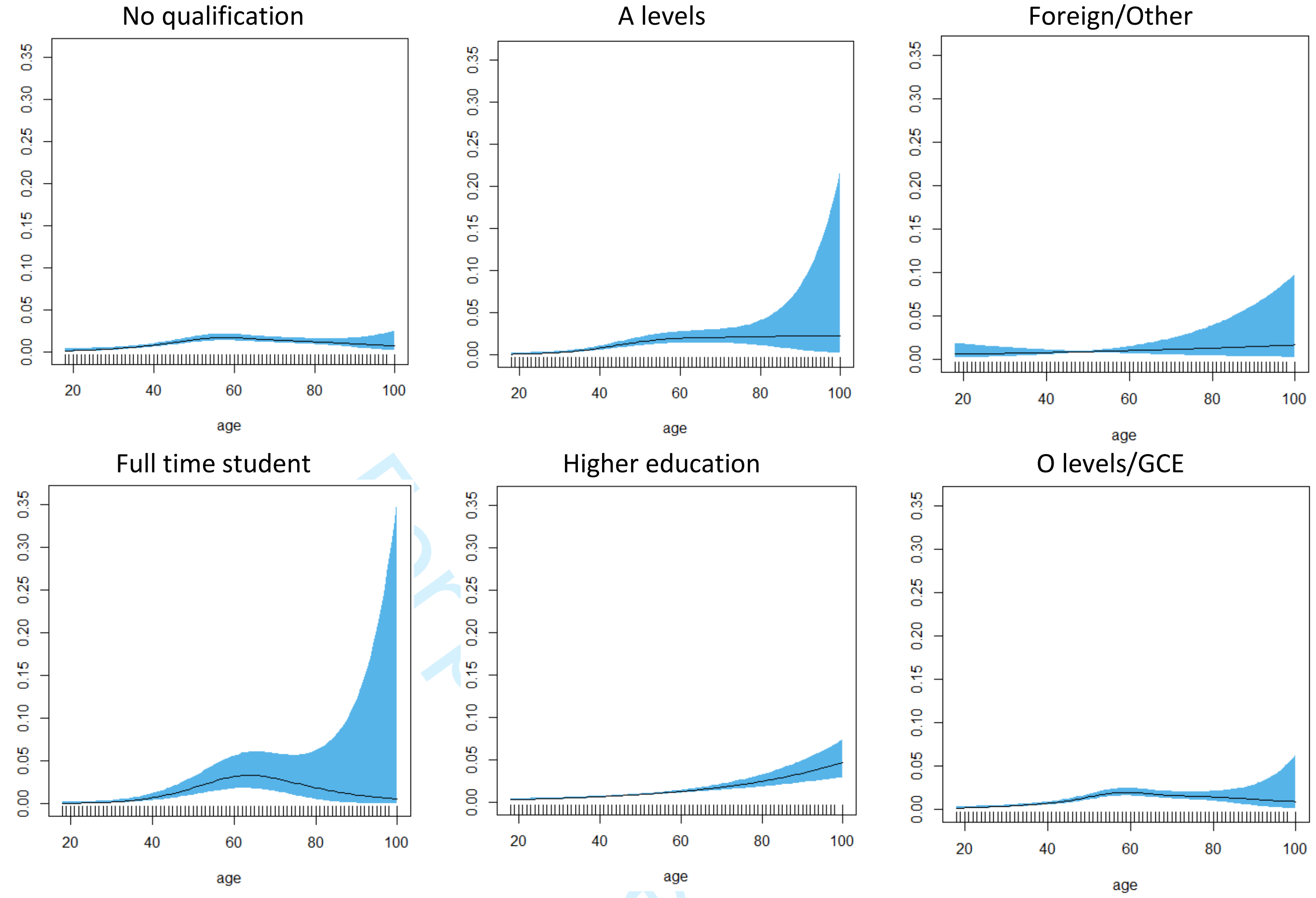

For peer review only

STROBE Statement—Checklist of items that should be included in reports of ***cohort studies***

	Item No	Recommendation	Page No
**Title and abstract**	1	(*a*) Indicate the study's design with a commonly used term in the title or the abstract	2
		(*b*) Provide in the abstract an informative and balanced summary of what was done and what was found	2
**Introduction**			
Background/rationale	2	Explain the scientific background and rationale for the investigation being reported	3
Objectives	3	State specific objectives, including any prespecified hypotheses	3-4
**Methods**			
Study design	4	Present key elements of study design early in the paper	4
Setting	5	Describe the setting, locations, and relevant dates, including periods of recruitment, exposure, follow-up, and data collection	4
Participants	6	(*a*) Give the eligibility criteria, and the sources and methods of selection of participants. Describe methods of follow-up	4
		(*b*) For matched studies, give matching criteria and number of exposed and unexposed	NA
Variables	7	Clearly define all outcomes, exposures, predictors, potential confounders, and effect modifiers. Give diagnostic criteria, if applicable	4
Data sources/ measurement	8*	For each variable of interest, give sources of data and details of methods of assessment (measurement). Describe comparability of assessment methods if there is more than one group	4
Bias	9	Describe any efforts to address potential sources of bias	4-5
Study size	10	Explain how the study size was arrived at	4 and 6
Quantitative variables	11	Explain how quantitative variables were handled in the analyses. If applicable, describe which groupings were chosen and why	4-5
Statistical methods	12	(*a*) Describe all statistical methods, including those used to control for confounding	5
		(*b*) Describe any methods used to examine subgroups and interactions	5
		(*c*) Explain how missing data were addressed	4
		(*d*) If applicable, explain how loss to follow-up was addressed	NA
		(*e*) Describe any sensitivity analyses	5
**Results**			
Participants	13*	(a) Report numbers of individuals at each stage of study—eg numbers potentially eligible, examined for eligibility, confirmed eligible, included in the study, completing follow-up, and analysed	5
		(b) Give reasons for non-participation at each stage	4-5
		(c) Consider use of a flow diagram	NA
Descriptive data	14*	(a) Give characteristics of study participants (eg demographic, clinical, social) and information on exposures and potential confounders	5-6
		(b) Indicate number of participants with missing data for each variable of interest	4-6
		(c) Summarise follow-up time (eg, average and total amount)	NA
Outcome data	15*	Report numbers of outcome events or summary measures over time	5

For peer review only - http://bmjopen.bmj.com/site/about/guidelines.xhtml

Main results	16	(*a*) Give unadjusted estimates and, if applicable, confounder-adjusted estimates and their precision (eg, 95% confidence interval). Make clear which confounders were adjusted for and why they were included	6-7
		(*b*) Report category boundaries when continuous variables were categorized	4
		(*c*) If relevant, consider translating estimates of relative risk into absolute risk for a meaningful time period	NA
Other analyses	17	Report other analyses done—eg analyses of subgroups and interactions, and sensitivity analyses	SM
**Discussion**			
Key results	18	Summarise key results with reference to study objectives	8-9
Limitations	19	Discuss limitations of the study, taking into account sources of potential bias or imprecision. Discuss both direction and magnitude of any potential bias	9
Interpretation	20	Give a cautious overall interpretation of results considering objectives, limitations, multiplicity of analyses, results from similar studies, and other relevant evidence	9
Generalisability	21	Discuss the generalisability (external validity) of the study results	9
**Other information**			
Funding	22	Give the source of funding and the role of the funders for the present study and, if applicable, for the original study on which the present article is based	10

*Give information separately for exposed and unexposed groups.

Note: An Explanation and Elaboration article discusses each checklist item and gives methodological background and published examples of transparent reporting. The STROBE checklist is best used in conjunction with this article (freely available on the Web sites of PLoS Medicine at http://www.plosmedicine.org/, Annals of Internal Medicine at http://www.annals.org/, and Epidemiology at http://www.epidem.com/). Information on the STROBE Initiative is available at http://www.strobe-statement.org.

For peer review only

For peer review only - http://bmjopen.bmj.com/site/about/guidelines.xhtml
